# Creating a brand. Genesis, transformation and diffusion of Qurayyah Painted Ware

**DOI:** 10.1371/journal.pone.0351296

**Published:** 2026-08-19

**Authors:** Marta Luciani, Pamela Fragnoli, William D. Gilstrap, Johannes H. Sterba, Doralice Klainscek, Joseph A. Greene, Stephen Bourke, Paul Donnelly

**Affiliations:** 1 University of Vienna, Department of Prehistoric and Historical Archaeology, Vienna, Austria; 2 Human Evolution and Archaeological Sciences (HEAS), University of Vienna, Austria; 3 Austrian Archaeological Institute (OeAI-OeAW), Unit for Archaeological Sciences, Vienna, Austria; 4 Massachusetts Institute of Technology, Center for Materials Research in Archaeology and Ethnology, Cambridge, Massachusetts, United States of America; 5 Technische Universität Wien, TRIGA Center Atominstitut, Center for Labelling and Isotope Production, Vienna, Austria; 6 Museum of the Ancient Near East, Harvard University, Cambridge, Massachusetts, United States of America; 7 Pella Excavations, NEAF, Archaeology, University of Sydney, Sydney, Australia; 8 Archaeology, University of Sydney, Sydney, Australia; University of Mazandaran, IRAN, ISLAMIC REPUBLIC OF

## Abstract

Based on a rigorous analytical and interdisciplinary study, this article offers the first investigation of the dynamics leading to the genesis of a brand. Elaborating beyond recent contributions on the phenomenon of ancient branding in past societies, we measure different parameters to understand when and how in the Bronze Age the trajectory leading to brand-making initiated. We investigate branding not through the analysis of added markers and/or packaging but through the development of commodities manufactured with cultural-specific production modes and unmistakable visual properties. This research offers the first integrated technological, petrographic and geochemical study of Qurayyah Painted Ware (**QPW**) analyzing dozens of stratified pottery samples from a pottery kiln in Qurayyah (NW Arabia), a site at the heart of a remarkable and long-lasting ceramic tradition. Our analyses reveal that this bichrome painted pottery (QPW)—locally invented in the late Middle Bronze Age (MBA)—developed over centuries into a location-based, technologically consistent and aesthetically distinctive corpus. Comparative data from Tell el-Kheleifeh and Pella (both Jordan) further confirm the ‘Qurayyah-ite’ origins of **QPW 1–2** and the later emergence of **QPW 4** as a widespread, choice export ware. We argue that the combination of technological continuity, visual identity, and wide regional dissemination makes QPW 4 an early example of brand-making in ceramic production. This study not only redefines the origins and diffusion of QPW but also offers a framework to understand how ancient objects mediated producer-consumer relationships across time and space.

## 1. Introduction

### 1.1. Developing a brand in the desert?

**Fig 1:**
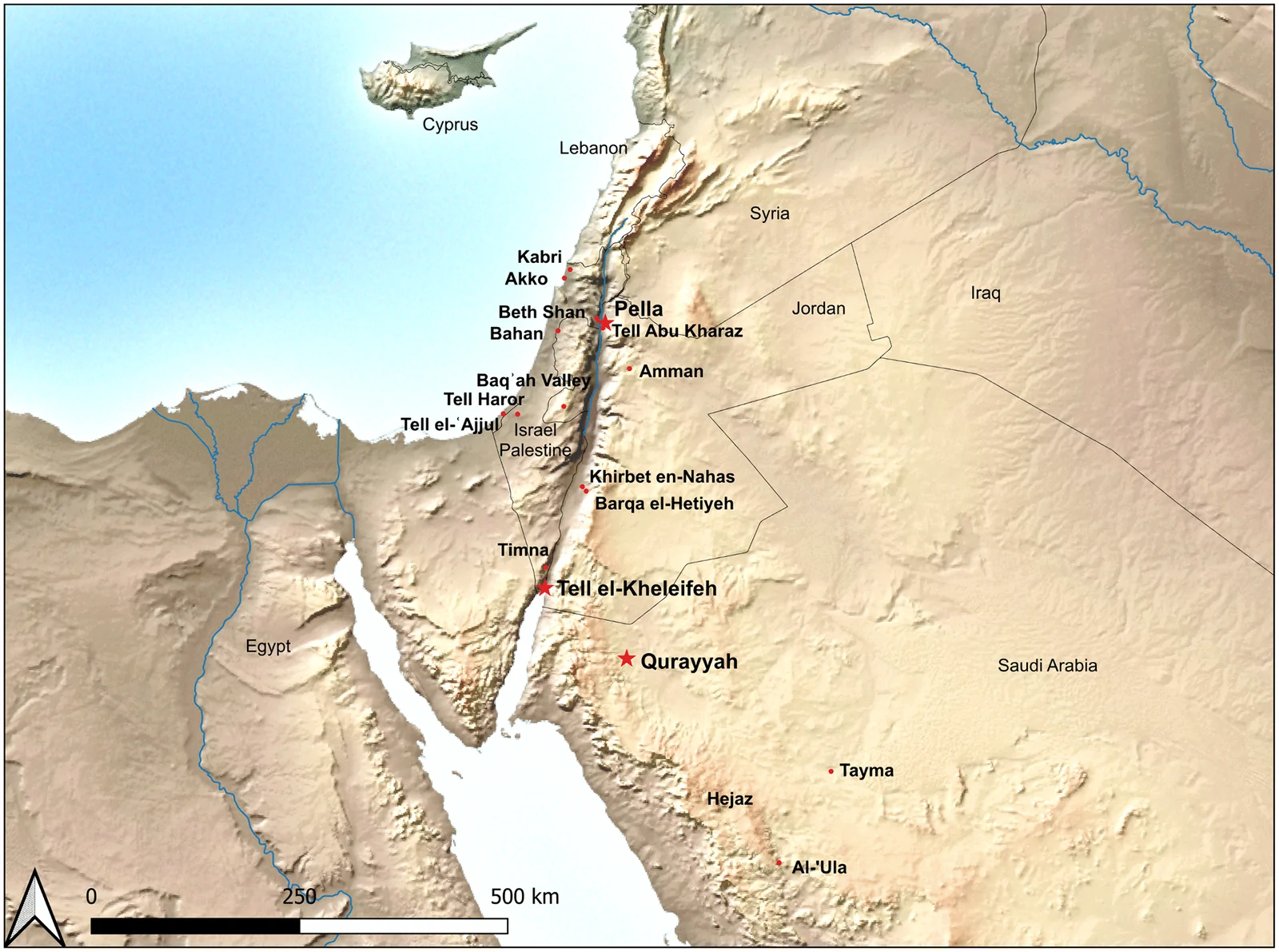
Regional map with the location of Qurayyah, Tell el-Kheleifeh, Pella, and the other sites mentioned in the text. Map created using QGIS; basemap data from Natural Earth (public domain, http://www.naturalearthdata.com/).

Until 10 years ago very little reliable information on the material culture of ancient North-West Arabia and its pottery was available. Thanks to current field work in the oases of Qurayyah [1–3] and Tayma [4] (Fig 1), it is now possible to shed light on technological and commercial networks in Arabia and the Greater Levant in the Bronze and Iron Ages. One major research question pertains to the formation and diffusion of a Hejazi painted ceramic found throughout the Southern Levant [5] and North Arabia, traditionally dubbed ‘Midianite Pottery’ [6] and later defined ‘Qurayyah Painted Ware’ (= **QPW**, [7]).

**Fig 2:**
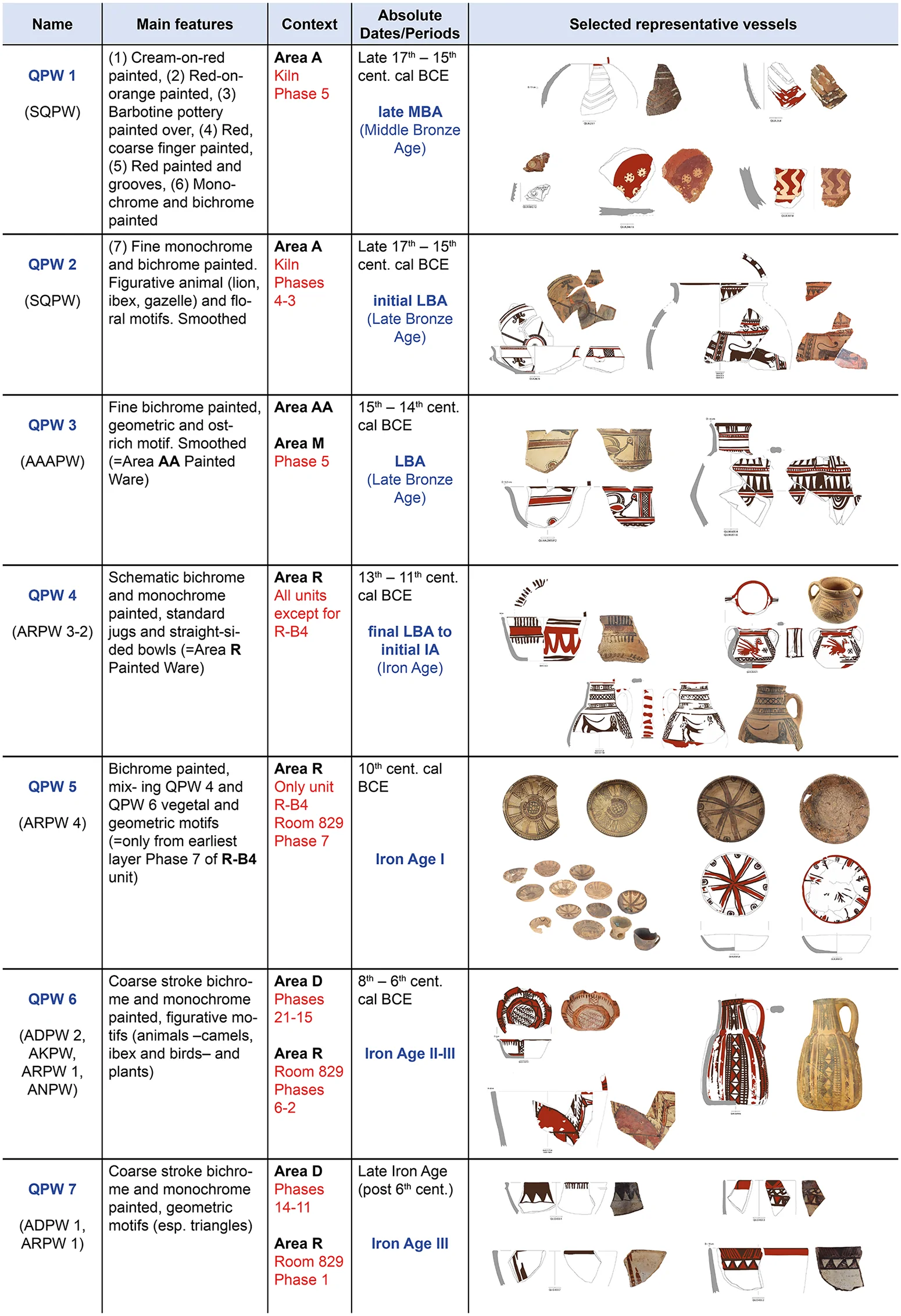
Concise table of attested QPW 1–7 assemblages, their findspots, absolute dates and period chronologies (Concept: M. Luciani; graphics: D. Klainscek). For details on ^14^C dates see S1 Graph.

Recent investigations in the frame of the Qurayyah Joint Archaeological Project [2] have corroborated the quintessential ‘Qurayyah-ite’ nature of this Hejazi pottery production [1,8], invented in the desert oasis of Qurayyah in the late MBA (Middle Bronze Age) to initial LBA (Late Bronze Age; see below QPW 1 and QPW 2 Fig 2) [2]. Notably, we have shown that QPW production consists not of one, but seven distinct, radiometrically dated (see S1 Graph) assemblages (**QPW 1**–**7**), developing throughout more than one millennium from the late MBA down to the IA III (Iron Age) in the 6^th^ cent. BCE and later (see below Fig 2). To reflect this, our new taxonomy adds a number to the old acronym QPW (i.e.,**QPW+Number** = QPW 1–7 [8]) to correctly classify and distinguish it from similar looking pottery previously and currently known as ‘**QPW (without number)**’ [5,9–11]. This renaming, based on having identified a ***sequence*** of productions, speaks to the fact that we understand the seven different manufactures evolving from the initial **QPW 1–2** as home-grown products. Moreover, we argue that not at the beginning but by the fourth iteration, i.e., **QPW 4**, this pottery manufacture, highly recognizable by the community at-large, was intentionally developed in Qurayyah as a brand.

Elaborating on preliminary studies [12], we present the macroscopic (morpho-typology; macrofabrics; technology), microscopic (petrography) and geo-chemical (NAA) study of 36 pottery samples of QPW 1–2, i.e., the earliest bichrome painted pottery production in Qurayyah. We aim to characterize it, understand its genesis [1] and later developments (**QPW 3**–**7**). Because the QPW 1–2 assemblages stem from the late MBA to initial LBA firing kiln in Area A in Qurayyah [2], they offer a reliable baseline for local bichrome painted pottery manufacture. While occasional technological [13], petrographic [14–15] or NAA [16] studies of single QPW vessels were carried out in the past, this is the first time all three methods are performed combined, on several dozen stratified samples stemming from deposits investigated through systematic, controlled excavations [2] (S1 Table).

To better frame our results, we added four samples from chronological periods/contexts at Qurayyah that are earlier and later than the kiln’s (two: Early Bronze Age [EBA] and two: final LBA/initial IA see Fig 2) and compared our evidence to two external sites: Tell el-Kheleifeh and Pella (both in Jordan). The latter sites provide us with less exhaustive but crucial data for evaluation. We present technology, petrography, and NAA of 9 QPW samples (S5 Table) from Tell el-Kheleifeh, Southern Jordan [17] and finally, to constrain our chemical data, we compare it with 200 NAA analysis of **CoW** (Chocolate on White) samples from Pella (S5 Table). We intend to characterize morphology, petrography and geochemical fingerprint of their respective manufactures, understand how QPW 1–7 evolved and identify regional production locations.

### 1.2. Identifying a brand in ancient pottery production

Pottery, much like other commodities found in prehistoric contexts, can be considered as situational entities that are enmeshed in social and cultural coding (e.g., discrete cultural expression [18]). The object, then, may be considered as the result of a production sequence guided by a “cultural and cognitive process” [19]. As such, we argue that both technological practices [20] and aesthetic effects [21–23] in the frame of the cultural specificity of design and color structures [24] as well as rules of corporate visual identity [22,23,25] are mechanisms of producer-consumer interaction leading to the creation of commodity/producer reputation [26]. When a homogenous and bounded entity defined by a standardized package of traits (as in, e.g., 2^nd^ cent. AD Lezoux sigillata [27]) is developed in a specific producing center (e.g., Aegean examples [28]) in a scale and distribution suggesting intentional trade [29], a process of brand-making is set in motion.

It is now understood that brands and proto-brands [18,23,30–32] existed well before modern economies. Past analyses have mostly focused on branding as transforming commodities into brands by adding a recognizable package or label, such as seal-impressed or inscribed clay sealings or tags [31,33] or marking the product with writing, signs or brand/trademarks [34]. However, in this contribution we analyze the process by which the commodity itself –decorated pottery vessels– was developed into a brand not by added symbols but on the virtue of its visual identity, material consistency, technological continuity, centralized production, export circulation, local imitation, intentional commercial scope, and cultural reputation (see below §6.2). While ***all*** these criteria are necessary for the definition of an ancient brand, a single center as product origin and the intentionality of the commercial scope distinguish it analytically from, e.g., widely traded ceramic wares with strong stylistic identity. The latter (e.g., Late Neolithic Halaf painted fine ware [35]) may exhibit remarkable stylistic coherence over large areas but are seldom standardized and their distribution reflects shared cultural traditions rather than the circulation of a product originating from a specific, single production centre. Here, we argue that after the in loco genesis [1] of Qurayyah’s earliest painted production (QPW 1–2), the development of five additional subsequent QPW 3–7 assemblages indicates the existence of a long-lasting technological tradition persevering in the desert oasis of Qurayyah (*contra* [13]) that with QPW 4 led to the creation of a brand. We investigate whether QPW 4 can be seen as a material instantiation of early branding, where aesthetic standardization, technological control, export packaged together in an intentional commercial enterprise created a recognizable and valued product, an active expression of identity.

## 2. Sites and contexts

### 2.1. Qurayyah

The walled river oasis of Qurayyah (28° 47’ 00” N and 36° 00’ 27” E), a megasite of over 300 hectares in extension, is being investigated by the Qurayyah Joint Archaeological Project (Ahmed M. Abualhassan – Heritage Commission of the Saudi Ministry of Culture and Marta Luciani – University of Vienna) since 2014 [2]. A permanent major settlement with monumental architecture since the late 4^th^ millennium BCE [36], it continued uninterrupted until the 1^st^ millennium CE. In different stratigraphically excavated areas, we could identify and date at least ten different pottery repertoires [36]. Starting with the late MBA, they were painted, mostly in two color chromatism, and are dubbed QPW 1–7 (Fig 2).

In order to establish a baseline of surely local ceramic manufacture, we investigated the discard material of a pottery firing kiln (excavation Area A). The double-chamber furnace consists of two superimposed units, a lower combustion chamber and an upper one divided by a pierced floor on which the vessels were stacked for firing and was used over three distinct phases [1] (S1 Appendix). We call the **Phase 5** pottery QPW 1 and the **Phases 4−3** production QPW 2 (Fig 2). The latter is characterized by a fine bichrome paint that in vessel shapes, decoration grammar and use of bichrome painting echoes ceramic manufactures that emerge in the late MBA around the Eastern Mediterranean, such as CoW [1].

Our dataset from the Area A pottery furnace includes one complete vessel and several thousand pottery sherds (3,209 alone are diagnostic fragments) showing traces of various stages of production: shaped and painted but not-yet-fired; fired but cracked, warped, partially overfired, vitrified, slag etc.

### 2.2. Tell el-Kheleifeh

The low mound of Tell el-Kheleifeh (29°32’49.03“N, 34°58’48.55”E) is located only half a kilometer from the present-day coastline of the eastern branch of the Red Sea, 130 km NW of Qurayyah. The half a dozen Hejazi(-looking) pottery fragments found in Tell el-Kheleifeh were initially called ‘Edomite’ [37], eventually ‘Midianite’ [37–39]. In a recent revision [17], a total of 14 sherds could be identified as being QPW. Here, QPW pottery is attested from the initial LBA down to the Iron Age II-III (Fig 2) indicating that the site’s connections to North Arabia were longer lived than hitherto hypothesized. However, there is a clear preponderance of material comparable to QPW 4, dated the final LBA/initial IA period (see §4.1 below and Fig 2).

### 2.3. Pella

The six-hectare site of Pella in Jordan (modern Tabaqat Fahl, 32°26’53.04“N-35°36’47.81”E) is located in the foothills of the east Jordan valley [40–42] approximately 400 km north of Qurayyah. In the late MBA, when Pella was a primary ‘gateway community’ [43], a distinctive decorated pottery assemblage, CoW [44], characterizes the painted pottery repertoire and is strongly reminiscent of contemporary QPW 2’s (7) Fine monochrome and bichrome painted (see S1 Table). One of our main research questions was establishing whether there were any connections between these painted manufactures attested from Jordan to North Arabia at the transition from the MBA to the LBA.

## 3. Methods

**Macroscopic analysis** primarily consisted of an encompassing morpho-typological and fabric classification of ceramic assemblages in order to select samples (36 QPW 1–2 plus an additional 4) for laboratory analyses and preliminary observation of manufacturing techniques (see S1 Table).

**Ceramic thin sections** were examined using the polarizing microscope LEICA DM 2700P and grouped based on textural, mineralogical, and technological features [45]. These groups represent specific modes and places of production [46], aiding in establishing directionality in product movement as well as structural links between producers and consumers.

**Neutron activation analysis** (**NAA**) determines elemental concentrations through nuclear processes [47]. Ceramic samples were analyzed following standard protocols at the Center for Labelling and Isotope Production in Vienna [48]. Elemental data were statistically evaluated using established approaches, including a modified Mahalanobis distance that accounts for measurement errors and between-sample dilution (best-relative fit factor [49]). All calculations were performed in R (Version 4.5.1 [50]). Resulting compositional groups and statistical outliers were compared with international ceramic compositional databases (e.g., Bonn, Demokritos, Missouri University Research Reactor (MURR), Oregon State University) using open repositories such as tDAR and compatible analytical standards [51–52], enabling provenance assessment through comparison with previously analyzed materials.

**Chemical and petrographic data** are complementary. Establishing a clearly defined production reference database with new analyses from Qurayyah, Pella, Tell el-Kheleifeh and in the future Tayma, Barqa el-Hetiye and other sites generates unprecedented insights into ceramic production, provenance, distribution, and patterns of consumption in this region over a lengthy timespan.

All necessary permits were obtained for the described study, which complied with all relevant regulations: permission to export and analyze the material from Qurayyah were granted by the head of the Saudi Commission for National Heritage (SCTH), now Heritage Commission of the Ministry of Culture of Saudi Arabia (Dr. Jasir el-Herbish, Dr. Abdullah al-Zahrani, Dr. Ajab Alotibi); permits for the material from Tell el-Kheleifeh were granted by Prof. Dr. Peter Der Manuelian, Director of the Harvard Semitic Museum (since 2020: HMANE) and Dr. Adam Aja. Permission to export and analyze the material from Pella was granted by Prof. Dr. Fawwaz al-Khraysheh, then Director-General of the Department of Antiquities, Jordan. Additional information regarding the ethical, cultural, and scientific considerations specific to inclusivity in global research is included in the Supporting Information (see S1 File)

## 4. Results of macroscopic investigations

### 4.1. Qurayyah

**Morpho-typological analysis and definition of the sample**: the QPW 1–2 assemblages constitute our baseline in the analysis of pottery produced on location. We chose 36 (S1 Table) vessel fragments (Q1 – Q28, Q30 – Q36, Q40) representative of the different phases of the kiln in Area A, in roughly equal amount from Phase 5 (= QPW 1 pottery: 17 fragments) and the later Phases 4−3 (= QPW 2 pottery: 19 fragments). Our sample (S1 Table) covers all functional/decoration classes identified: cooking pots, simple ware, two unfired specimens and vessels belonging to the following groups: (1) Cream-on-red painted, (3) Barbotine pottery sherds painted over, (4) Red, coarse finger painted, (5) Red or black paint and incisions or grooves, (6) Monochrome and bichrome painted, (7) Fine monochrome and bichrome painted (S1 Table). Based on their stratigraphic distribution, we understand class (1) Cream-on-red painted to be an exclusively QPW 1 production with (3), (4), (5) and (6) also prevalently belonging to QPW 1. The (7) Fine monochrome and bichrome painted class, while present in small numbers in the QPW 1 repertoire, more than doubles in the younger QPW 2 and can be considered its most characteristic group.

**Macrofabrics**: We have a total of 19 different macrofabric types attested in the Area A kiln, defined based on the type, color, size and density of mineral inclusions. In this initial sampling, we have included and analyzed at least one specimen of each macrofabric (S3 Table). In the older QPW 1 repertoire, we identified 13 different macrofabrics (in 17 samples) and only four of them (F02.01, F02.07, F02.08, F02.12) were used twice. In the following QPW 2, 13 different macrofabrics were used in 19 samples. Only one (F02.05) was used for 21% of the specimens and two further macrofabrics (F02.03 and F02.08) for 12%. The significant increment of use of single macrofabrics (especially F02.03 and F02.05) attests a recognizable trend towards reduction of the variety from the older (Phase 5 = QPW 1) to the younger phases of the kiln (Phases 4−3 = QPW 2). This assessment is further sustained by the comprehensive observations of over 200 specimens studied in depth for the final publication (Unearthing Qurayyah I) and preliminary evaluation of all over 3000 sherds found in Area A.

**Manufacturing**: By analyzing technology of the QPW 1–2 sample (QPW 1 Table 1; QPW 2 Table 2), we found evidence of hand-making, wheel-coiling and potentially wheel-throwing (S1 Table). In 97% of the sample (n = 34) the chosen method is wheel-coiling, where a coil-built roughout was shaped and subsequently placed on the potter’s wheel for refinement [53–54]. Preforming with RKE (rotational kinetic energy) is visible in the well smoothed surfaces bearing traces like parallel striations (Q21, Q23) or stretched topographic features (Q11). Some specimens show clear signs of their assembled structures, like undulated profiles (Q3, Q14, Q31) or irregular breaks extending on the surface (Q7). Others (Q8, Q9, Q10, see below Table 2) indicate a higher modification by RKE, resulting in thin-walled vessels with a strong orientation of inclusions. A more precise determination of the employment methods of RKE [55] will be developed in future studies. We can nevertheless state that in the QPW 1–2 assemblages, the process of fashioning was carried out meticulously, denoting a high degree of artisanship in mastering the technique.

**Table 1:** QPW 1 samples from Qurayyah.

Sample no.	Description (see also S1 Table for drawings and photos)	Petro Group	NAA
**Q1**	QPW 1 (4) Red, coarse finger painted ‖ Body sherd, closed shape	Qurayyah 3	loner
**Q6**	QPW 1 (4) Red, coarse finger painted ‖ Body sherd, neck of closed shape	Qurayyah 3	loner
**Q11**	QPW 1 (5) Red painted and grooves ‖ Body sherd, small jar/juglet	Qurayyah 2b	loner
**Q12**	QPW 1 (4) Red, coarse finger painted ‖ Base, open shape	Qurayyah 2a	loner
**Q13**	QPW 1 Simple ware ‖ Rim and body sherd, carinated bowl	Qurayyah 1b	loner
**Q14**	QPW 1 (7) Fine bichrome painted ‖ Rim and body sherd, bowl	Qurayyah 2b	loner
**Q18**	QPW 1 (1) Cream-on-red painted ‖ Base, open shape	Qurayyah 1b	V032
**Q19**	QPW 1 (1) Cream-on-red painted ‖ Body sherd, open shape	Qurayyah 1b	V032
**Q22**	QPW 1 (3) Barbotine pottery painted over ‖ Handle, closed shape(?)	Qurayyah 1b	loner
**Q23**	QPW 1 (6) monochrome painted ‖ Rim and body sherd, bowl	Qurayyah 1b	V033
**Q25**	QPW 1 (3) Barbotine pottery painted over ‖ Body sherd, closed shape	Qurayyah 1a	V032
**Q26**	QPW 1 Cooking pot ‖ Rim and body sherd, applied rope decoration	Qurayyah 1a	V032
**Q27**	QPW 1 Cooking pot ‖ Rim with ledge handle	Qurayyah 1b	#021
**Q31**	QPW 1 (7) Fine monochrome painted ‖ Complete shape, straight-sided bowl	Qurayyah 2b	V033
**Q33**	QPW 1 (4) Red, coarse finger painted ‖ Rim, juglet	Qurayyah 1b	V032
**Q36**	QPW 1 (7) Fine monochrome painted (unfired) ‖ Rim and body sherd, small beaker	Qurayyah 2a	loner
**Q40**	QPW 1 (4) Red, coarse finger painted ‖ Rim and body sherd, jug	Qurayyah 1c	V032

QPW 1 samples (late MBA) from the Phase 5 pottery kiln in Area A in Qurayyah

**Table 2:** QPW 2 samples from Qurayyah.

Sample no.	Description (see also S1 Table for drawings and photos)	Petro Group	NAA
**Q2**	QPW 2 Simple ware ‖ Base, closed shape	Qurayyah 2a	loner
**Q3**	QPW 2 Simple ware ‖ Base and body sherd, small beaker	Qurayyah 3	loner
**Q4**	QPW 2 (7) Fine bichrome painted ‖ Rim and body sherd, carinated bowl	Qurayyah 2a	loner
**Q5**	QPW 2 (7) Fine bichrome painted ‖ Rim and body sherd, small beaker	Qurayyah 2a	loner
**Q7**	QPW 2 (7) Fine bichrome painted ‖ Rim and body sherd, beaker	Qurayyah 2a	loner
**Q8**	QPW 2 (7) Fine bichrome painted ‖ Body sherd, neck of jar/jug	Qurayyah 2a	loner
**Q9**	QPW 2 (7) Fine bichrome painted ‖ Body sherd, globular jar	Qurayyah 2b	V033
**Q10**	QPW 2 (7) Fine bichrome painted ‖ Rim, carinated bowl	Qurayyah 2b	loner
**Q15**	QPW 2 (7) Fine bichrome painted ‖ Rim and body sherd, small beaker	Qurayyah 2a	loner
**Q16**	QPW 2 (4) Red, coarse painted ‖ Base, closed shape	Qurayyah 1d	loner
**Q17**	QPW 2 Simple ware ‖ Rim, large krater	Qurayyah 1c	loner
**Q20**	QPW 2 Cooking pot ‖ Rim and body sherd	Qurayyah 2a	loner
**Q21**	QPW 2 (7) Fine bichrome painted ‖ Rim, bowl	Qurayyah 1b	#021
**Q24**	QPW 2 Cooking pot ‖ Rim and grooved neck	Qurayyah 1a	V032
**Q28**	QPW 2 Cooking pot ‖ Rim with applied rope decoration	Qurayyah 1b	loner
**Q30**	QPW 2 Cooking pot ‖ Rim with applied rope decoration	Qurayyah 1a	loner
**Q32**	QPW 2 (3) Barbotine pottery painted over ‖ Rim and body, hole-mouth pot	Qurayyah 1c	V032
**Q34**	QPW 2 (7) Fine monochrome painted ‖ Rim, bowl	Qurayyah 1b	loner
**Q35**	QPW 2 Simple ware (unfired) ‖ Body sherd, closed shape	Qurayyah 1b	loner

QPW 2 samples (initial LBA) from the Phases 4−3 pottery kiln in Area A in Qurayyah

In order to better constrain our results, beyond the 36 specimens from the pottery furnace, we have sampled four additional pieces (S1 Table): two ***more ancient*** than the kiln, i.e., **Q37** and **Q39** (dated resp. EBA IV and EBA II) (Table 3). They are both hand-made and display clearly distinct macrofabrics if compared to QPW 1–2. The two samples ***more recent*** than the kiln, i.e., QPW 4 samples (**Q29** and **Q38** Table 4) are both wheel-coiled, as seen in QPW 1–2, and belong to the same macrofabric groups as the latter. However, the use of wheel-coiling in the overall QPW 4 assemblage is characterized by more accentuated undulations on the vessels’ profiles.

**Table 3:** EBA II-IV samples from Qurayyah.

Sample no.	Description (see also S1 Table for drawings and photos)	Petro Group	NAA
**Q37**	Barbotine, applied wavy lines and dots ‖ Body sherd, globular jar	Qurayyah loner	loner
**Q39**	Simple ware ‖ Body sherd, open shape	Qurayyah loner	loner

Early Bronze Age samples from Area B (Q37 – EBA IV) and Area M (Q39 – EBA II) in Qurayyah

**Table 4:** QPW 4 samples from Qurayyah.

Sample no.	Description (see also S1 Table for drawings and photos)	Petro Group	NAA
**Q29**	QPW 4 Bichrome painted ‖ Rim and body sherd, bowl	Qurayyah 1d	loner
**Q38**	QPW 4 Bichrome painted ‖ Rim, bowl	Qurayyah 1a	loner

QPW 4 samples (final LBA/initial IA) from Area A (Q29) and Area R (Q38) in Qurayyah

### 4.2. Tell el-Kheleifeh

Recently [17], an additional 8 Hejazi-looking fragments were identified beyond the original half dozen published by Glueck [37], for a total of 14 QPW specimens ([17]and S2 Table). Of these, only nine were present at the HMANE (ML005-ML013) and could undergo petrographic and NAA analyses (see below § 5.2 and 5.4). Wherever decoration is sufficiently well preserved, these QPW-like vessels may be classified typo-chronologically in different periods on the basis of the sequence now established in Qurayyah ([2,3,8] and Table 5):

**Table 5:** Analyzed samples from Tell el-Kheleifeh.

Sample no.	Description (see also S2 Table for drawings and photos)	Findspot in Tell el-Kheleifeh	Comparison with Qurayyah’s assemblages	Proposed date	Petro Group	NAA
**ML005**	Bichrome painted flat base	Room 50	= QPW 6	Iron Age II-III	Kheleifeh 1	loner
**ML007**	Bichrome painted neck of jug	Room 30	= QPW 4	Final LBA/initial IA	Kheleifeh 1	V032
**ML008**	Monochrome painted body sherd, closed shape	Debris North of Room 43	= QPW 6?	Iron Age II-III?	Kheleifeh 1v	#022
**ML009**	Monochrome painted rim of straight-sided bowl	Trench, square J 14, debris	= QPW 4	Final LBA/initial IA	Kheleifeh 1	V032
**ML010**	Monochrome painted rim of bowl	Trench, Square J 14, debris	= QPW 6	Iron Age II-III	Kheleifeh 1v	#022
**ML011**	Monochrome painted body sherd, closed shape		= QPW 3?	Late Bronze Age?	Kheleifeh 1	loner
**ML012**	Monochrome painted handle	Top of Mound between Room 50 and 80	= QPW 4	Final LBA/initial IA	Kheleifeh 1	loner
**ML013**	Bichrome painted base	[Trench] J 16	= QPW 4	Final LBA/initial IA	Kheleifeh 1	V032
**ML014**	Bichrome painted body sherd	Square “I” 15 at about 35 cm below surface. April 22 1940	= QPW 4?	Final LBA/initial IA?	Kheleifeh 1	loner

Samples from Tell el-Kheleifeh retrieved in the HMANE, dating to different periods and analyzed in this study.

Three aspects must be underlined: in Tell el-Kheleifeh, QPW represents the minority of the material found, i.e., does not look indigenous. However, its presence was protracted in time: it started in the initial LBA (ML003 = QPW 2 or QPW 3, see Fig 2 for chronology) and continued for another millennium until the Iron Age II-III (ML005, ML010 and possibly ML008 = QPW 6, Fig 2). The third point is that there is a clear preponderance of QPW 4 material (ML007, ML009, ML012-ML014), i.e., of the final LBA to initial IA, (Fig 2) when Qurayyah’s production emerged as a recognizable brand for export and is widely attested across the Southern Levant [5,9,10].

### 4.3. Pella

CoW ware is a consistent but small (2−3%) presence within MBA/LBA period ceramic assemblages at Pella, both on the main mound [44,56], and in the tomb-fields to the east [40] and south [41,42,57]. Relative chronological considerations would suggest CoW assemblages are confined to the MB III and LB I periods and fall within the absolute date range of 1600−1450 BCE [58,59]. Thus, CoW is attested for an identical chronological spectrum as Qurayyah’s QPW 1–2 and several aspects of shape (carinated bowls; jugs with painted shoulders), surface treatment (white/light slip) and decoration syntax (partitioned design) are also strongly evocative of the reciprocal assemblages [1,17].

P. Donnelly established clear definitional boundaries between CoW and White Slip Burnished Painted (WSBP), White Slip Burnished (WSB), and White Slip Painted (WSP) and initiated a large (171x, see S5 Table) NAA study of CoW and these potentially related fabrics [44]. CoW and CoW-related materials included samples from across the Southern Levant (Beth Shan, Tell Abu Kharaz, Bahan, Kabri, Akko, Baqʾah Valley, Amman, Tell Haror, and Tell el-ʿAjjul).

Considering the typological, distributional and chemical datasets together confirmed the initial surmise that CoW was a product of the central Jordan Valley. The most likely explanation for the appearance of CoW is that it arose as a local response to the blossoming of distinctive regional painted ceramics (both manufactured in situ and imported) in the later MBA ceramic assemblages of the Southern and Central Levant.

## 5. Results of microscopic investigations

### 5.1. Qurayyah’s petrography

The thin sections from Qurayyah were classified into three petrographic groups primarily based on the type, frequency and size of inclusions. The largest group―**Qurayyah 1** (see S2 Appendix for detailed descriptions and photos)―comprises 21 out of 40 samples (see above Tables 1 and 2), is dominated by angular argillaceous rocks (shales) and was subdivided into 4 subgroups (Qurayyah 1a-1d) with decreasing density of inclusions.

The second group―**Qurayyah 2** (see S3 Appendix)―includes 14 samples (see above Tables 1 and 2), characterized by dominant, well-sorted and angular grains of monocrystalline quartz. A few samples (Q8, Q10, Q9) stand out due to a pronounced orientation of inclusions and voids, likely indicating the use of rotative devices during the manufacturing process. Five samples (Q9, Q10, Q11, Q14, Q31) are distinguished by a higher proportion of shales and a lower content of sandstones, resulting in their classification into a distinct subgroup (Qurayyah 2b).

A third group―**Qurayyah 3** (see S4 Appendix)―consists solely of three samples Q1, Q3 and Q6 (see above Tables 1 and 2), which are almost devoid of sedimentary rocks and exhibit a finer texture with a mode of 0.25 mm and an incidence ranging from 3 to 5%.

Petrographic analysis of pottery surely produced in Qurayyah’s furnace was fundamental for establishing the initial QPW 1–2 in situ production baseline.

That the two QPW 4 samples Q29 and Q38, four centuries later, were still fashioned with the local kiln’s major petrogroup (Qurayyah 1) suggests that in the late MBA a trajectory started, which by the final LBA had become a long established tradition. Finally, the two samples **Q39** and **Q37** (EBA II and EBA IV and thus older than the 36 + 2 samples above) could not be classified into any groups and are loners (see S4 Appendix). Sample Q39 is distinguished by dominant calcareous sedimentary stones. Q37 is rich in shales, sandstones and quartz, but, unlike the samples of petrogroups Qurayyah 1 and 2, it is distinguished by a higher incidence of K-feldspars as well as the presence of plagioclases, brown hornblendes, epidotes and olivines.

The minerals and rocks present in the three petrographic groups (Qurayyah 1–3) align with the local geology, especially with the Silurian Qalibah group predominant in the northwestern region of the Tabuk province [60]. This group mainly consists of silty and micaceous claystones of various colors, alongside fine-grained sandstones. Our petrographic group Qurayyah 1 aligns with petrographic group 6 as identified by Smith and colleagues [15] for painted wares found in Khirbat en-Nahas (Jordan) and thus our findings support their interpretation of this petrogroup as import from Qurayyah [15]. Our and Smith et al.’s [15] petrographic groups match, in turn, with pottery from Timna analyzed by Rothenberg and Glass [14]. Conversely, the mineral composition of elder (i.e., EBA IV) loner Q37 does not align with local formations. Some of the minerals observed therein may be associated with Late Tertiary volcanic rocks cropping out approximately 20 km to the south of Tabuk, alongside sedimentary rocks of the Tayma formation. Calcareous rocks like those found in Q39 (dated EBA II) are typically located along the southwestern Arabian coast and thus far are not attested in the pottery produced locally in Qurayyah’s furnace. That petrographic characteristics of pottery from before the late MBA significantly differ from the ceramic produced in the kiln underscores once more the intentional and creative character of the change initiated with the production of QPW 1–2 in the local furnace in Qurayyah. Eventually, centuries later, this development evolved into a standardized tradition that resulted into the creation of a brand (QPW 4).

### 5.2. Tell el-Kheleifeh’s petrography

Petrographic analysis of the nine Tell el-Kheleifeh sherds present in the HMANE resulted in a single coherent petrographic group with a single nuanced variant. Seven of the nine samples comprise the fabric group **Kheleifeh 1** (see above, Table 5 and S5 Appendix). This fabric is composed of a mica-rich clay matrix with angular reddish argillaceous rock (Sedimentary Rock – Type 1). The groundmass contains monocrystalline quartz and alkali feldspar particles, and fragments of sedimentary rock (Type 2). A variant, **Kheleifeh 1v** (Table 5 and S5 Appendix), is similar in microstructure, but shows distinctively differing inclusions and textures. Both samples (ML008 and ML010) that form this variant are dated to the Iron Age II-III. Sample ML010 is oxidized only near the vessel margins, leaving a large dark core. The shallow oxidation can be attributed to a short period of time at the maximum firing temperature [61–62].

In terms of provenance, the mineralogical and petrological composition of both the Kheleifeh 1 and Kheleifeh 1v is compatible with the geological landscape of the Tabuk Formation on which the site of Qurayyah is located. The Tabuk area maintains exposed Ordovician sediments and sedimentary rocks in the Arabian Tabuk Formation and the Saq Formation series [63] and includes iron-rich, bedded shaly sandstones with variegated clast grain size ranging from sandy to silty [63]. These shaly-sandstone formations are acutely similar to Sedimentary Rock – Type 1 identified as a tempering agent in Kheleifeh 1. The Tabuk series also includes both greenish grey to grey, slightly micaceous siltstone and subordinate micaceous silty shale [63], similar to that observed as the tempering agent in Kheleifeh 1v.

### 5.3. Qurayyah’s NAA (S4 Table)

The resulting compositional data from the 40 analyzed samples produced two homogeneous chemical groups, V032 and V033. The larger, **V032**, consists of eight samples (Q18, Q19, Q24, Q25, Q26, Q32, Q33, Q40), the majority of which belong to the oldest layer of the kiln (Phase 5) assemblage, i.e., QPW 1 (see Tables 1 and 2), making this group the earliest baseline of local Qurayyah bichrome painted pottery manufacture.

A second chemical group, **V033**, comprises three samples (Q9, Q23 and Q31). Although they belong to distinct but related classes (QPW 1 (6) and QPW 1/2 (7)), they are all dark monochrome painted (Tables 1 and 2 and see also S2 Graph).

Moreover, two samples, Q21 and Q27, form a chemical pair (**#021**). All other samples are chemical loners, i.e., they could not yet be associated with any other samples in the dataset or in the databases used for comparison thus far. A comparative search of the ceramic composition databases described above failed to produce any relationship with any existing reference group.

### 5.4. Tell el-Kheleifeh’s NAA

Of the nine samples from Tell el-Kheleifeh analyzed, three final LBA/initial IA, QPW 4-like sherds (ML007, ML009 and ML013) clearly fit Qurayyah’s chemical group V032 (Table 5). Considering the latter’s local, ‘Qurayyah-ite’ nature, this match strongly suggests that these vessels had most likely been produced in the North Arabian oasis and exported north to Tell el-Kheleifeh. This is consistent with petrographic results since all samples are members of fabric group Kheleifeh 1.

Further analysis shows that the two samples from Kheleifeh 1v (ML008 and ML010) form a chemical pair (**#022**) that is discrete from any other chemical group in this study or within the known chemical databases. The remaining five samples are chemical loners. Elemental concentration data for all samples measured is available in the S4 Table.

### 5.5. Pella’s NAA

NAA raw data from CoW pottery from Pella (S5 Table) compared with the data from Qurayyah show that there is no overlap in the clay and elemental make-up between CoW and QPW 1–2 assemblages from Qurayyah (S4 Table). Notwithstanding visual parallels and being coeval, these two manufactures developed locally and independently from each other from earlier MBA traditions.

## 6. Discussion

### 6.1. Qurayyah’s, Tell el-Kheleifeh’s and Pella’s bichrome painted ceramic wares

The combination of morpho-stylistic, technological, petrographic and chemical analyses that follows outlines a detailed picture of raw material exploitation, recipe preparation and ceramic manufacture dynamics. The detailed study of the *chaînes opératories* of production processes and organization will be dealt with in future encompassing analyses.

When compared to the elemental data, it becomes evident that the potters of **Qurayyah** during the late MBA to initial LBA (see Fig 2) were producing a wide variety of QPW 1–2 pottery types with a limited range of raw materials. Statistical treatment of elemental concentrations indicates that the majority of the sherds, which make up petrogroup Qurayyah 1 correspond to both chemical groups V032 and V033 and to pair #021. Considering the petrographic subgrouping of Qurayyah 1, this much chemical variability makes sense. Both chemical groups, V032 and V033, discretely correlate and reflect the most recurrent compositions; furthermore, there is a petrographic match with the geological formations around the site. NAA results showing a coherent picture of clusters of chemical composition matching the major petrographic group reinforce the definition of the ‘Qurayyah-ite’ baseline of production. This tradition is still extant centuries later in the oasis (see below comparison with Tell el-Kheleifeh) suggesting that, when it developed, QPW 4 relied on a long-established practice and the role of Qurayyah as major production center.

The very homogeneous group (V032/Qurayyah 1) and two further chemical pairs/matches (V033 and #021) stem mostly from the oldest use of the kiln (Phase 5). The younger phases of use of the kiln (Phases 4−3) have a higher proportion of chemically unassigned sherds than Phase 5: 79% and 47% respectively. We interpret this as an increased variability either in the procurement and/or in the processing of raw materials.

From the morpho-stylistic perspective, in the oldest phase of the furnace, potters developed different decoration techniques as if they experimented with multiple aesthetic outcomes (see above Fig 2, QPW 1: types (1), (3), (4) and (6) see S1 Table). In the younger phases of the kiln, against increased variety in clay compositions, they achieved a more uniform decoration style (QPW 2, type (7) see S1 Table). This important development is visible also in the kiln’s structural history, developing from a downdraft furnace to an updraft furnace (Phases 4 and 3, see S1 Appendix). This indicates a remarkable change in firing strategy to be synchronous with the observed shift in procurement of raw materials and decoration between the older and younger phases of the furnace: potters were experimenting and creating a trajectory for the development of their local product.

Among the samples not found in the furnace, the eldest, EBA II simple ware Q39 and EBA IV Barbotine Pottery Q37, are outliers both petrographically and geochemically. The minerals and rocks contained in Q37suggest a possible origin from the south of Tabuk.

The youngest samples thus far analyzed, i.e., the QPW 4 specimen (Q29 and Q38), show that petrogroup Qurayyah 1 was still in use several hundred years after its initial development, i.e., Qurayyah potters persevered in the local raw materials and manufacturing tradition while introducing a higher degree of standardization leading to brand-making (see below §6.2).

Moving to **Tell el-Kheleifeh**, the vessel sherds exhibit homogeneous minero-petrographic associations, and heterogeneous trace element concentrations (44% loners and 2 groupings: V032 and #022). The samples petrographically align with the formations around Qurayyah, and three QPW 4-like samples datable to the final LBA/initial IA period (ML007, ML09 and ML013) belong to Qurayyah’s chemical group V032. The use of geologically compatible iron-rich argillaceous rock as temper in conjunction with the similarities in clay matrix and firing regimes suggests a technological link to the earlier fabrics found in the Qurayyah Bronze Age kiln (QPW 1–2). These findings suggest that the three QPW 4-like Tell el-Kheleifeh vessels attributed to Qurayyah’s chemical group V032 were imported from Qurayyah.

The two matches for Qurayyah’s chemical group V032 found in the Berkeley database (i.e., **QRY0024** and **QRY0026** [64]) are said to come from Qurayyah and thus add evidence to our identified local baseline. More importantly, also two sherds from the Hathor Shrine in Timna (Site 200), Israel (**TIM0029** and **TIM0034** [64]) match the Qurayyah geochemical group V032. Therefore, the best current explanation is that beyond Tell el-Kheleifeh, also Timna (Site 200) used vessels produced in Qurayyah. The narrow number of QPW 4 pieces proven to have been exported agrees well with this brand’s non-utilitarian, symbolic and iconic character and its unique and conspicuous visual identity.

The wide variety in clay procurement may make the identification of discrete and matching chemical groups a challenge. Because chemical compatibility, for now, is found in single occurrences in each site, we do not claim that all Hejazi-looking/QPW 4-like pottery in the Southern Levant was necessarily imported from Qurayyah. While this was Rothenberg and Glass’ opinion, based on their encompassing petrographic analysis [14]: “one workshop or a number of workshops in Qurayyah and its surroundings”, also confirmed by chemical analyses [65], others assert (unpublished evidence, [66]) that in the Southern Levant, pottery imported from this oasis was present alongside local production, such as in the Wadi Faynan smelting sites imitating Qurayyah’s QPW 4 brand.

In sum, ***in the late MBA/initial LBA*** (Fig 2), despite the overall visual similarities between Qurayyah’s QPW 2 and Pella’s CoW, analytical results clearly indicate these to be two distinct manufactures. Contrariwise, ***in the final LBA*** (Fig 2), the chemical signature of at least three Tell el-Kheleifeh QPW 4-like sherds and two from Timna is identical to the locally produced pottery baseline established in Qurayyah since the final MBA/initial LBA (QPW 1–2), and their petrography (also Khirbat en-Nahas’s [15]) and morpho-stylistic and technological features fully match Qurayyah’s contemporary QPW 4. It seems likely that the QPW 4 ceramic brand had also become a product for export.

### 6.2. Anatomy of an ancient brand

While different definitions of brand exist depending on the specific context/past society in which it is embedded, there is agreement on understanding a brand as “a mode of connectivity between producer and consumers” [67], both a conveyor of information (on origin and quality/authenticity) and conveyor of image/meaning (value, status, identity) [30].

Analytically, we propose to recognize the creation of a brand on ***eight*** necessary criteria (see below Table 6): (1) visual identity, (2) consistency of chosen raw material, which together with (3) continuity in technology led to standardization of procedures, i.e., a well-established, manufacturing tradition, (4) centralized production, bound to place-of-origin: e.g., ‘Qurayyah-ite’, (5) export circulation, instances of (6) local imitation (e.g., Wadi Faynan), (7) intentional commercial enterprise indicating production of commodities by ***destination*** [26] and finally (8) cultural reputation.

**Table 6:** Criteria defining a brand.

	Branding Element	QPW Evidence
1	Visual identity	Consistent bichrome motif and evolving style across centuries (QPW 2–4), recognizable across regions, mark of authenticity and identity. Decoration is part of product identity, not mere embellishment, because painted decoration is not on a small part of the assemblages (as in ‘fine wares’), but constitutes between 50%−90% of the whole repertoire.
2	Material consistency	Recurrent petrographic/geochemical signature from QPW 1–2 to QPW 4 pointing to a shared production locus
3	Technological continuity	Standardized paste preparation, shaping, firing, and finishing/painting procedures
4	Centralized production	Archaeologically documented kilns complex and production debris in the oasis of Qurayyah
5	Export circulation	Supra-regional distribution traceable to Qurayyah’s QPW 4 production hub (e.g., Tell el-Kheleifeh, Timna, Barqa el-Hetiye, Khirbat en-Nahas, likely Tayma, and Al-ʿUla)
6	Local imitation	Presence of stylistic copies outside the production area indicating recognition and emulation (Barqa el-Hetiye [66])
7	Intentional commercial scope	Scaled-up manufacturing indicating a developed and intentional commercial operation, including commodification-for-export
8	Cultural reputation	Found in hundreds of exemplars in elite burials, miniatures, symbolic and cultic vessels [8,23]; rules of corporate visual identity spilling over cross-craft to other media (e.g., stone masonry [22]) – imply aura and value beyond utilitarian

As to (1), because our approach understands vessel form and decoration style as formal structures, systems of visual communication [24], shaped both by conscious learning and intentional expression, we estimate visual identity to be one relevant element of branding. Ethnographic research tells us that “potters treat decorative styles as systems of knowledge” [24] that mark and signify social and cultural meanings and boundaries [24]. A standardized unique and conspicuous visual identity was achieved in the QPW 4 production [8,23].

Criteria (2) – (3) examined in this study proved consistency in choice of local raw materials as well as overall technology from paste preparation to finishing enduring over centuries, thus pointing to the establishment of an increasingly standardizing manufacturing tradition, from QPW 1–2 to QPW 4 and beyond. (4) Centralized production is proven by the presence in Qurayyah of a kilns-area continuing over a long time-span, with consistent evidence of QPW 4 by-products such as slag and overfired vessels [23]. (5) Proof of spatial dissemination of commodities created for export is given by the above data on petrography and chemical composition of QPW 4 vessels found in foreign regions matching ‘Qurayyah-ite’ vessels. (6) QPW 4 was imitated abroad [66], because perceived as desirable brand. (7) The hundreds of conspicuously decorated QPW 4 vessels suggest the pent-up scale and volume of production [8], an intentional commercial manufacture, beyond strictly local needs. (8) Cultural reputation and brand status were established not only by the multiple uses of QPW 4 beyond the utilitarian [8] but by the normative character of decoration rules spilling over cross-craft beyond the medium of pottery also to stone carving [22] and two-color chromatism painted on clay being used as ersatz in skeuomorphs of sumptuary goods [23] suggesting corporate visual identity.

## 7. Conclusions: branding through technological continuity, regional consistency and an intentional trajectory to standardization and trade

Through our study, we have shown that at the onset of the Bichrome Era [13] in the late MBA in the Eastern Mediterranean and Arabia (at least two) independent ceramic manufactures―**CoW** in Pella (Jordan) and **QPW 1**–**2** in Qurayyah (North Arabia)―developed. While they intentionally resembled each other and featured communalities with other contemporary productions in the region (e.g., Cypriot and Levantine Bichrome Painted Pottery, Egyptian New Kingdom painted pottery and possibly even the earlier Cypriot WS I [1]), they remained autonomous. This finding proves interconnectedness, inter-regional communication and awareness of one another, coupled with a distinctly ***idiosyncratic*** development of each own’s material culture. The often-invoked phenomena of imitation or hybridity are not at play in this period.

At this time, in Qurayyah’s pottery furnace new fabrics and recipes were developed for **QPW 1**–**2** using local geological formations to establish a distinct, homegrown ‘Qurayyah-ite’ repertoire. Because decorative styles, i.e., similarities between QPW and other late MBA manufactures, could have easily been transmitted horizontally [68], identifying through the present study that the QPW 1–2 production was indigenous was paramount in clearly classifying this manufacture as original and grassroot.

The continuation of painted pottery production (**QPW 3**, see Fig 2 above), in the centuries after the initial creation of QPW 1–2, indicates the establishment of a real potting tradition, ever evolving, but clearly rooted in the desert environment of the oasis. Moreover, 3–400 years after the invention of QPW 1–2, in the final LBA, the continued evolution of the local manufacturing tradition established in the Qurayyah hub led to the creation of the standardized bichrome painted series called QPW 4 (formerly known as ‘Midianite Pottery’ or, more recently, QPW without number, see above §1.1). We can clearly recognize the genetic relationship both in raw materials, technology, iconographic themes and style between QPW 1–2, QPW 3 and QPW 4 and can thus be sure that shapes and painted motifs were the development of the own original ware, not an import from Aegean prototypes (*contra* [5]).

Qurayyah’s **QPW 4** (see Fig 2 above) was not only exported abroad―to Tell el-Kheleifeh and Timna (see above § 6.1), Khirbat en-Nahas [15] and possibly to other North Arabian oases such as Taymaʾ [69] and Al-ʿUla [70]―but also cherished as local burial gift in interments of elite-professionals(?) [8,22] and as symbolic representation in miniature vessel [8]. Ceramic fabrics and chemical compositions are maintained in Qurayyah from QPW 1–2 to QPW 4, as is the practice of bichrome painting―although motifs are constantly modified and finishing technology may become less accurate. That QPW painted pottery production tradition was entrenched in the desert oasis of Qurayyah is proven by the continuation―with repeated modifications and evolutions [3]―of a bichrome painted manufacture with consistent raw materials even beyond QPW 4, throughout the entire Iron Age (**QPW 5–7**) down to the 4^th^ century BCE before the appearance of Nabatean painted wares.

From the onset of the QPW 1–2 pottery manufacture in the late MBA/initial LBA, we understand this creation as an intentional, bottom-up and successful experiment in kickstarting in a desert oasis of North Arabia a production hub for a unique but pan-regionally familiar pottery manufacture. The discontinuity in shapes, decoration and fabrics with preceding local EBA IV productions and the ***entanglement*** of the new production with the other late Middle Bronze Age repertoires of the Bichrome Era in the Greater Levant and Eastern Mediterranean [1] vouch for intentionality. The home-grown character and enduring ***location*** of the firing kilns inside the bounded confines of the settlement [71] speak of its local, ‘Qurayyah-ite’ origin and identity for both producers and outsiders.

It is uncertain whether the brand that became QPW 4 was the intention of the QPW 1–2 potters from the start. However, the aligned and yet idiosyncratic character of QPW 1–2 indicates that Qurayyah artisans were not imitating others [1 and 3], and by intentionally creating a clearly recognizable ceramic ware of their own, set in motion the trajectory for the later brand-making development.

By the time the QPW 4 manufacture was established, the sheer quantity of standardized vessels produced in the local kilns of the Qurayyah hub [8] and the cross-craft compliance to the norms of QPW 4 established visual identity in commodities other than pottery vessels such as stone carving, jewelry and miniatures [22–23]. Together with export abroad, they all indicate a streamlined commercial organization of a production with high reputational value: a brand was created.

## Supporting information

S1 Table Drawings, striking image, photos and macroscopic description of each analyzed pottery sample from Qurayyah. (PDF)

S2 Table Drawings, photos and macroscopic description of each analyzed pottery sample from Tell el-Kheleifeh. (PDF)

S3 Table Catalogue of mentioned macrofabrics from Qurayyah. (PDF)

S1 Appendix Plans and sections of the pottery kiln (Phases 5, 4 and 3) excavated in Area A in Qurayyah. (PDF)

S1 Graph Modelled and calibrated ^14^C dates of organic samples from Qurayyah. (PDF)

S2 Appendix Detailed descriptions and micrographs of petrogroup Qurayyah 1. (PDF)

S3 Appendix Detailed descriptions and micrographs of petrogroup Qurayyah 2. (PDF)

S4 Appendix Detailed descriptions and micrographs of petrogroup Qurayyah 3 and loners from Qurayyah. (PDF)

S5 Appendix Detailed descriptions and micrographs of petrogroups Kheleifeh 1 and Kheleifeh 1v. (PDF)

S4 Table Raw data from NAA analysis on samples from Qurayyah and Tell el-Kheleifeh. (ZIP)

S2 Graph Element by element comparison of chemical groups V032 and V033. (PDF)

S5 Table Raw data from NAA analysis on pottery from Pella. (ZIP)

S1 File Questionnaire on inclusivity in global research. Completed version of the questionnaire on inclusivity in global research.
(PDF)
