# Supplementary material for: Creating a brand. Genesis, transformation and diffusion of Qurayyah Painted Ware
Source: PLoS One. 2026 Aug 19;21(8):e0351296. doi: 10.1371/journal.pone.0351296 (PMC13489407; doi:10.1371/journal.pone.0351296)
Supplement: Supplementary file 1 [file pone.0351296.s001.pdf]

CREATING A BRAND.
GENESIS, TRANSFORMATION AND DIFFUSION OF QURAYYAH PAINTED WARE

Luciani M. – Fragnoli P. – Gilstrap W.D. – Sterba J.H. – Klainscek D.– Greene J.A. – Bourke S. – Donnelly P.

Corresponding author: Marta Luciani, University of Vienna, marta.luciani@univie.ac.at

…….

SUPPORTING INFORMATION 1

Drawings, photos and macroscopic description of each analyzed pottery sample from Qurayyah

All drawings and photos: rights holder Marta Luciani

Sam-ple no.	Sherd no.	Drawing	Photo	Fabric no.	Fabric colour	Firing	Surface out	Surface in	Surface colour out	Surface colour in	Pro-duction	Definition
Q1	QU.A.298.27	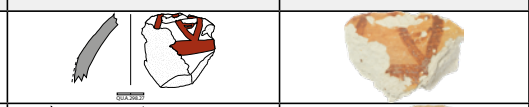		F02.01	7.5YR 8/1	H	sl, sm	s-sl, sm	5YR 5/6	7.5YR 8/2	W-C?	QPW 1 (4)
Q2	QU.A.300.9	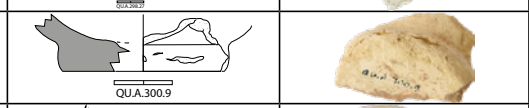		F02.01	2.5Y 8/4	H	no sl, sm	no sl, sm	2.5Y 8/4	2.5Y 8/4	W-C	QPW 2 (sw)
Q3	QU.A.287.1	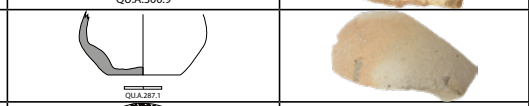		F02.02	10YR 8/1	H	s-sl, sm	s-sl, sm	2.5Y 8/3	10YR 8/2	W-C	QPW 2 (sw)
Q4	QU.A.29.1	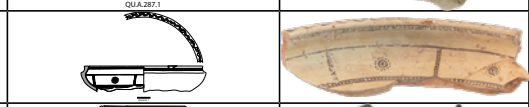		F02.03	2.5YR 7/6	H	sl, sm	sl, sm	10YR 8/4	10YR 8/4	W-C	QPW 2 (7)
Q5	QU.A.32.8	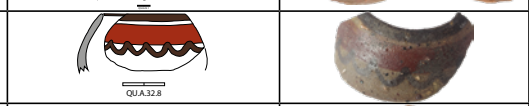		F02.03	2.5YR 4/1	H	s-sl, sm	s-sl, sm	7.5YR 4/1	7.5YR 4/1	W-C	QPW 2 (7)
Q6	QU.A.298.25	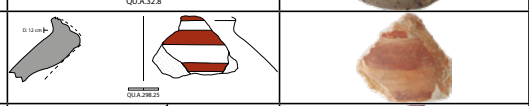		F02.04	7.5YR 8/2	H	sl, sm	/	5YR 7/6	/	W-C?	QPW 1 (4)
Q7	QU.A.286.2	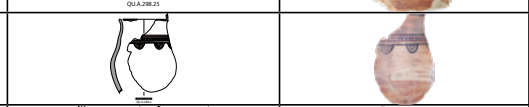		F02.05	5YR 6/6	H	sl, sm	sl, sm	10YR 8/4	5YR 6/6	W-C	QPW 2 (7)
Q8	QU.A.30.7	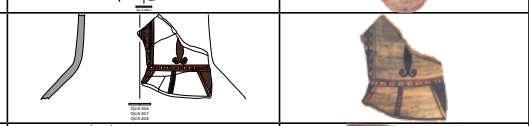		F02.05	5YR 6/6-<br>7.5YR 3/1	NH	sl, sm	sl, sm	10YR 8/4	10YR 8/4	W-C	QPW 2 (7)
Q9	QU.A.30.12	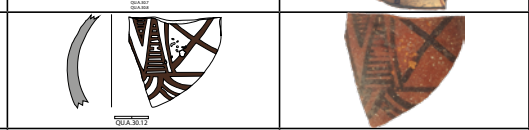		F02.05	2.5YR 5/6-<br>GLEY2 5/10B	NH	sl, sm	sl, sm	2.5YR 4/4	2.5YR 4/8	W-C	QPW 2 (7)
Q10	QU.A.286.244	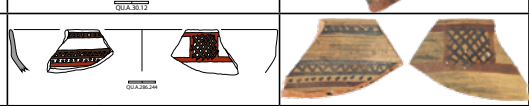		F02.05	2.5YR 6/6	H	sl, sm	sl, sm	10YR 7/6	10YR 5/4	W-C	QPW 2 (7)
Q11	QU.A.502.22	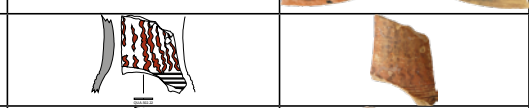		F02.07	10YR 8/4	H	sl, sm	sl, sm	2.5YR 6/6	5YR 6/8	W-C	QPW 1 (5)
Q12	QU.A.502.48	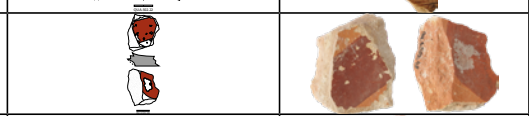		F02.07	10YR 8/4	H	sl, sm	sl, sm	2.5YR 6/8	2.5YR 6/8	W-C?	QPW 1 (4)
Q13	QU.A.502.42	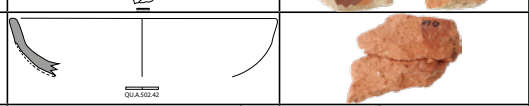		F02.08	2.5YR 6/6	H	sl, sm	sl, sm	10R 4/8	10R 4/8	W-T?	QPW 1 (sw)
Q14	QU.A.36.25	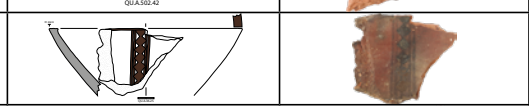		F02.08	2.5YR 6/6	H	s-sl, sm	sl, sm	2.5YR 4/2	2.5YR 4/3	W-C	QPW 1 (7)
Q15	QU.A.4.21	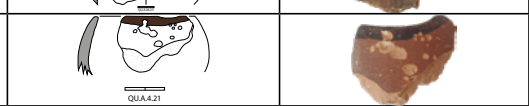		F02.08	5YR 7/6	H	sl, sm	s-sl, sm	2.5YR 4/4	2.5YR 5/6	W-C	QPW 2 (7)
Q16	QU.A.31.4	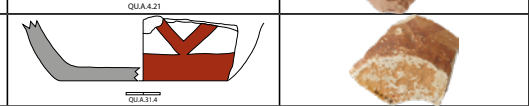		F02.08	10YR 8/4	H	sl, sm	sl, sm	2.5YR 6/6	2.5YR 6/6	W-C	QPW 2 (4)
Q17	QU.A.1.3	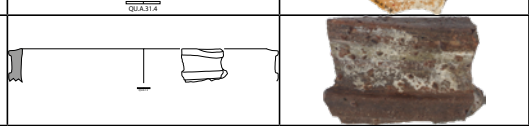		F02.09	2.5R 5/6-<br>GLEY2 4/10B	NH	sl, sm	sl, sm	5YR 3/1	2.5Y 4/1	W-C?	QPW 2 (sw)
Q18	QU.A.298.13	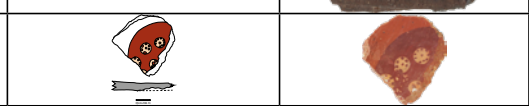		F02.10	5YR 7/6	H	no sl, sm	sl, sm	5YR 6/2	10R 5/6	W-C	QPW 1 (1)
Q19	QU.A.507.8	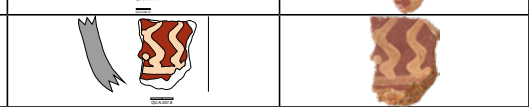		F02.11	2.5YR 4/6	H	sl, sm	sl, sm	2.5YR 5/3	10R 3/4	W-C	QPW 1 (1)
Q20	QU.A.7.1	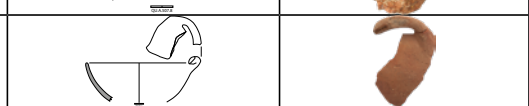		F02.12	2.5YR 4/4	H	no sl, sm	sl, sm	2.5YR 5/3	2.5 YR 4/6	W-C	QPW 2 (cp)
Q21	QU.A.4.7	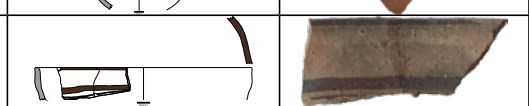		F02.12	10YR 4/1	NH	no sl, sm	sl, sm	10YR 3/1	7.5YR 4/2	W-C	QPW 2 (7)
Q22	QU.A.34.13	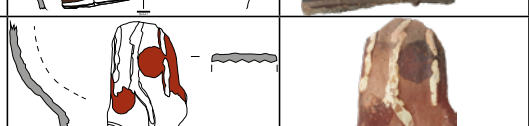		F02.12	2.5YR 6/6	H	s-sl, sm	/	2.5YR 5/6 -<br>2.5YR 5/3	/	HM<br>(handle)	QPW 1 (3)
Q23	QU.A.36.8	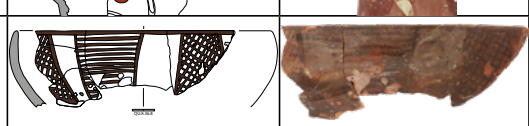		F02.12	2.5YR 4/4-<br>10YR 4/1	NH	sl, sm	sl, sm	2.5YR 5/3	2.5YR 5/2	W-C	QPW 1 (6)
Q24	QU.A.10.8	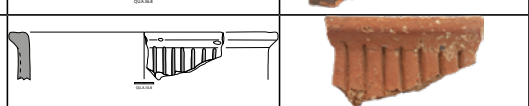		F02.13	10YR 8/6	H	sl, sm	sl, sm	2.5YR 6/6	5YR 5/6	W-C?	QPW 2 (cp)
Q25	QU.A.33.1	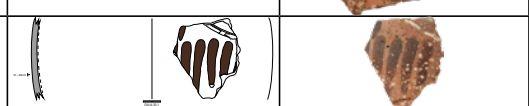		F02.13	2.5YR 6/6	H	sl, sm	/	2.5YR 6/6	/	W-C?	QPW 1 (3)
Q26	QU.A.22.8	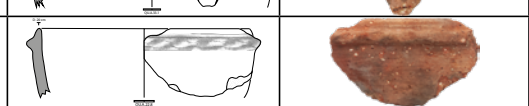		F02.14	2.5YR 5/4	H	sl, sm	sl, sm	2.5YR 4/3	2.5YR 4/4	W-C	QPW 1 (cp)
Q27	QU.A.22.7	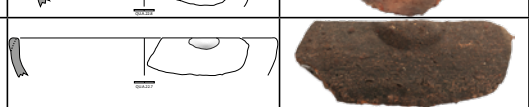		F02.15	7.5YR 5/1	NH	sl, sm	sl, sm	5YR 3/1	7.5YR 4/1	W-C	QPW 1 (cp)
Q28	QU.A.30.64	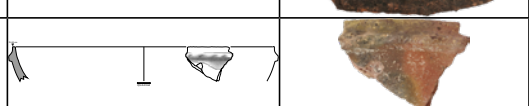		F02.16	2.5YR 5/4	H	no sl, sm	no sl, sm	2.5YR 5/4	2.5 YR 5/6	W-C	QPW 2 (cp)
Q29	QU.A.21.1	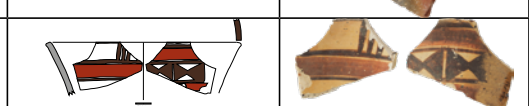		F02.17	2.5Y 8/2	H	sl, sm	sl, sm	10YR 8/4	10YR 7/6	W-C	QPW 4
Q30	QU.A.30.22	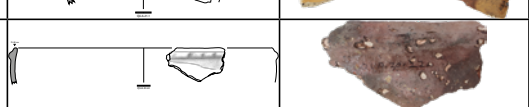		F02.18	5YR 4/2	H	sl, sm	sl, sm	10R 5/3	2.5YR 4/4	W-C	QPW 2 (cp)
Q31	QU.A.507.4	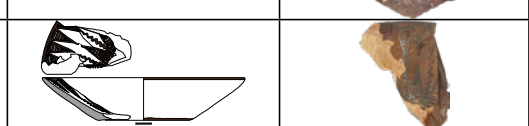		F02.19	7.5YR 7/6-<br>7.5YR 4/1	NH	sl, sm	sl, sm	5YR 5/2	5YR 4/3	W-C	QPW 1 (7)
Q32	QU.A.29.2	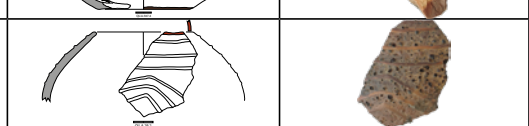		F02.20	7.5YR 5/1	H	sl, un.	sl, un.	2.5YR 5/3-<br>7.5YR 7/3	2.5YR 5/4	W-C	QPW 1* (3)
Q33	QU.A.637.8	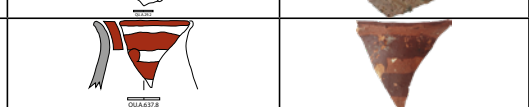		F02.21	10YR 8/6	H	sl, sm	sl, sm	2.5YR 5/4	2.5YR 7/6	W-C	QPW 1 (4)
Q34	QU.A.286.69	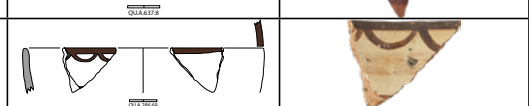		F02.21	7.5YR 6/6	H	sl, sm	sl, sm	10YR 7/3	10YR 8/4	W-C	QPW 2 (7)
Q35	QU.A.286/32.F.1	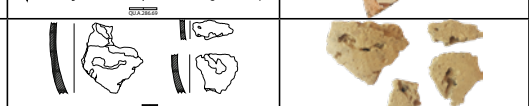		F02.15	10YR 7/4	/	no sl, sm	no sl, sm	10YR 7/4	10YR 7/4	W-C	QPW 2 (sw)
Q36	QU.A.505.F.1	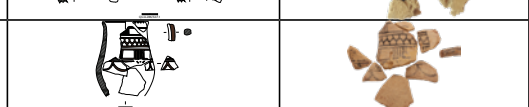		F02.01	10YR 8/4	/	sl?, sm	sl?, sm	10YR 8/4	10YR 8/4	W-C	QPW 1 (7)
Q37	QU.B.61.3	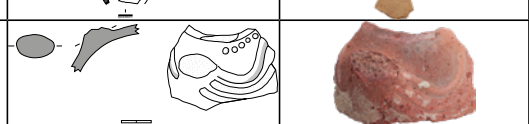		F01.05	10R 4/4	H	sl, sm	no sl,<br>non-sm	10R 5/4	10R 6/3	HM	EBA IV –<br>Barbotine
Q38	QU.R.1302.16	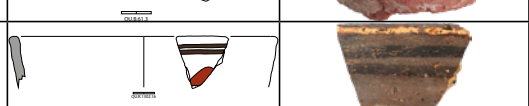		F02.09	7.5YR 7/4	H	sl, sm	sl, sm	5YR 5/2	2.5YR 5/1	W-C	QPW 4
Q39	QU.M.857.2	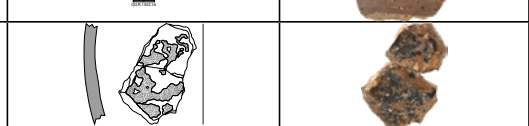		F00.01	7.5YR 6/4-<br>10YR 5/1	NH	sl, sm	sl, non-sm	10R 5/2	5YR 5/6	HM	EBA II (sw)
Q40	QU.A.637.4	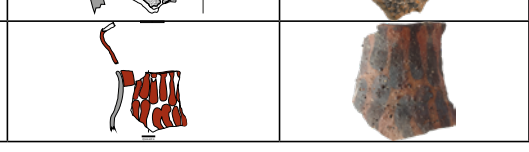		F02.20	7.5YR 5/1	H	sl, un.	sl, un.	2.5YR 5/3-<br>2.5YR 4/4	2.5YR 5/3	W-C	QPW 1 (4)

In order to avoid uncertainties in classification and for maximum transparency and follow-up, we commit to always publish photos and drawings of front and back of each single vessel/fragment (including surface finds, example below QU.S.9099.1, graphic scale 2cm) examined for technology and sampled for petrographic and geochemical analyses.

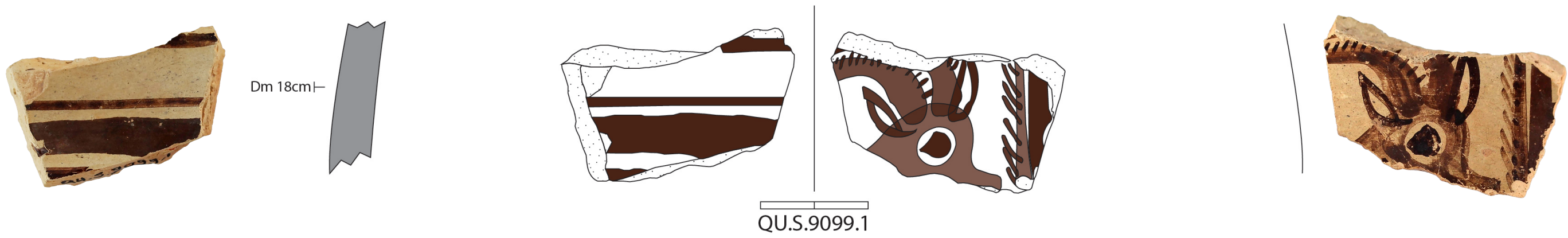

Our observations on technology are systematic but preliminary, limited to a macroscopic assessment of firing, manufacturing technique and surface finishing. We plan to expand on this subject in the future.

In the QPW 4 assemblage, too, we have documented a percentage of potentially wheel-thrown vessels similar (4%) to the one in the QPW 1-2 repertoire (Klainscek D. (2024) Two Pottery Assemblages from Area R, Qurayyah: a Pre-liminary Characterization of the Final Late Bronze Age to Initial Iron Age Ceramics, MA Thesis, University of Vienna).

Abbreviation key

Firing:	H	›	homogeneous
	NH	›	non-homogeneous
Surface:	sl	›	slip
	s-sl	›	self-slip
	no sl	›	no slip
	sm	›	smoothed
	non-sm	›	non-smoothed
	un.	›	untreated
Production:	HM	›	hand-made
	W-C	›	wheel-coiled
	W-T	›	wheel-thrown
Definition:	EBA	›	Early Bronze Age
	LBA	›	Late Bronze Age
	IA	›	Iron Age
	QPW	›	Qurayyah Painted Ware (See Fig. 2)
	(sw)	›	simple ware
	(cp)	›	cooking pot
	QPW 1 (1)	›	cream-on-red painted
	QPW 1 (3)	›	barbotine pottery sherds painted over
	QPW 1 (4)	›	red, coarse finger-painted
	QPW 1 (5)	›	red or black paint and incisions or grooves
	QPW 1 (6)	›	monochrome and bichrome painted
	QPW 1/2 (7)	›	fine (bichrome/monochrome) painted

*Although found in Phase 4-3, these fragments can be typologically assigne to assemblage QPW 1
