# Supplementary material for: Creating a brand. Genesis, transformation and diffusion of Qurayyah Painted Ware
Source: PLoS One. 2026 Aug 19;21(8):e0351296. doi: 10.1371/journal.pone.0351296 (PMC13489407; doi:10.1371/journal.pone.0351296)
Supplement: Supplementary file 2 [file pone.0351296.s002.pdf]

CREATING A BRAND.
GENESIS, TRANSFORMATION AND DIFFUSION OF QURAYYAH PAINTED WARE

Luciani M. – Fragnoli P. – Gilstrap W.D. – Sterba J.H. – Klainscek D.– Greene J.A. – Bourke S. – Donnelly P.

Corresponding author: Marta Luciani, University of Vienna, marta.luciani@univie.ac.at

……….

SUPPORTING INFORMATION 2

Drawings, photos and macroscopic description of each analyzed pottery sample from Tell el-Kheleifeh

ML001 to ML011 drawings and photos: rights holder Marta Luciani
ML012 to ML014 drawings: rights holder Marta Luciani
ML012 to ML014 photos: rights holder Joseph A. Greene

Sample no.	Sherd no.	Drawing	Photo	Petro - gr.	Fabric colour	Firing	Surface out	Surface in	Surface colour out	Surface colour in	Pro-duction	Proposed date
ML001	248	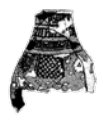	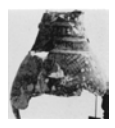									Final LBA – Initial IA ≈ QPW 4
ML002	3016	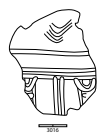	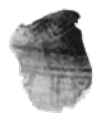									Final LBA – Initial IA ≈ QPW 4
ML003	7079	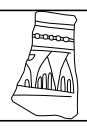	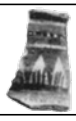									Initial LBA ≈ QPW 2/3
ML004	10,082	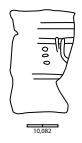	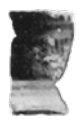									Final LBA – Initial IA ≈ QPW 4
ML005	10,083	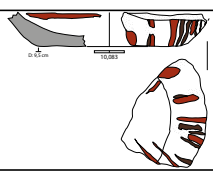	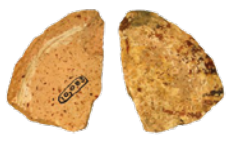	Khe-leifeh 1	7.5YR 6/2	H	sl	sl	10YR 7/4	7.5YR 6/2	W-C?	Mid-IA ≈ QPW 6
ML 006	Whereabouts unknown											
ML007	10,057	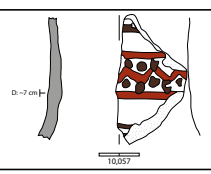	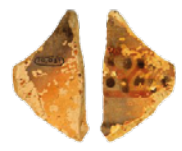	Khe-leifeh 1	10YR 7/3	H	sl	sl	10R 5/1	7.5YR 6/6	W-C?	Final LBA – Initial IA ≈ QPW 4
ML008	10,093	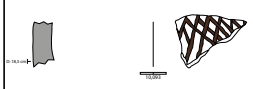	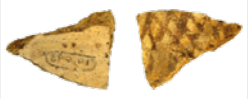	Khe-leifeh 1v	10YR 6/3	H	sl	sl	10YR 6/3	10YR 6/4	W-C?	Mid-IA? ≈ QPW 6?
ML009	11,016 (a)	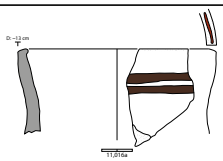	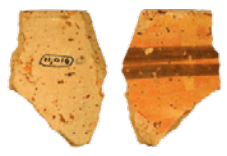	Khe-leifeh 1	10YR 7/4	H	sl	sl	7.5YR 6/6	10YR 7/4	W-C?	QPW 4
ML010	11,016 (b)	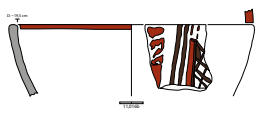	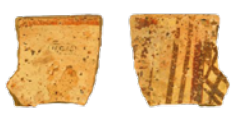	Khe-leifeh 1v	10YR 7/4	H	sl	sl	10YR 7/4	10YR 7/3	W-C?	Mid-IA ≈ QPW 6
ML011	no ID	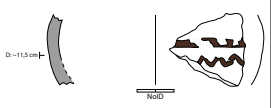	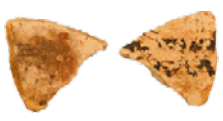	Khe-leifeh 1	5YR 8/2	H	sl	sl	5YR 8/3	5YR 5/4	W-C?	LBA? ≈ QPW 3?
ML012	8016	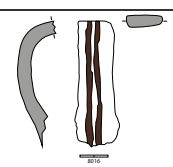	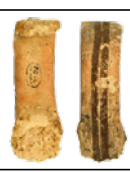	Khe-leifeh 1	10YR 7/3	H	sl	sl	10YR 7/4	7.5YR 7/4	W-C?	QPW 4
ML013	11,047	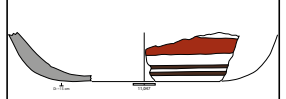	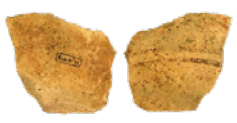	Khe-leifeh 1	10YR 7/3	H	no sl	no sl	10YR 8/4	10YR 7/4	W-C?	Final LBA – Initial IA ≈ QPW 4
ML014	11,094	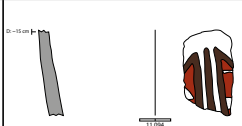	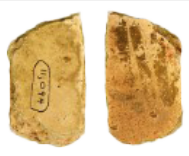	Khe-leifeh 1	10YR 6/4	H	sl	sl	10YR 6/4	10YR 7/4	W-C?	Final LBA – Initial IA? ≈ QPW 4?

Abbreviation key

Firing:	H	›	homogeneous
	NH	›	non-homogeneous
Surface:	sl	›	slip
	s-sl	›	self-slip
	no sl	›	no slip
	sm	›	smoothed
	non-sm	›	non-smoothed
	un.	›	untreated
Produc-tion:	HM	›	hand-made
	W-C	›	wheel-coiled
	W-T	›	wheel-thrown
Defini-tion:	EBA	›	Early Bronze Age
	LBA	›	Late Bronze Age
	IA	›	Iron Age
	QPW	›	Qurayyah Painted Ware (See Fig. 2)
	(sw)	›	simple ware
	(cp)	›	cooking pot

Defini-tion:	QPW 1 (1)	›	cream-on-red painted
	QPW 1 (3)	›	barbotine pottery sherds painted over
	QPW 1 (4)	›	red, coarse finger-painted
	QPW 1 (5)	›	red or black paint and incisions or grooves
	QPW 2 (7)	›	fine (bichrome/monochrome) painted

In order to avoid uncertainties in classification and for maximum transparency and follow-up, we commit to always publish photos and drawings of front and back of each single vessel/fragment examined for technology and sampled for petrographic and geochemical analyses.

Specimens ML001-ML004 were not in the holdings of the Harvard Semitic Museum (now HMANE) and could thus neither be thoroughly checked nor be sampled for analysis. However, shape and motifs on ML001-ML002 (comparable with QPW 4), ML003 (comparable with QPW 2 or 3) are clear either from Glueck's (1967: Glueck N. Some Edomite pottery from Tell el- Kheleifeh. BASOR. 1967; 188: 8-38. https://doi.org/10.2307/1356246) drawing, photo or both. ML004's photo is barely intelligible but the accurate description points to a comparison with QPW 4.

M. Luciani: The existence of a plurality of QPW assemblages with different chronological span has only become apparent after my original study of this material at the then Harvard Semitic Museum (spring 2016). Therefore, in Luciani 2018 (Luciani M. Pottery from the "Midianite Heartland"? On Tell Kheleifeh and Qurayyah Painted Ware New Evidence from the Harvard Semitic Museum. In: Nehmé L, Al-Jallad A, editors. To the Madbar and Back Again: Studies in the Languages, Archaeology, and Cultures of Arabia dedicated to Michael CA Macdonald. Leiden: Brill; 2018. pp. 392-438), I did not comment on the different chronologies of Tell el-Kheleifeh's QPW sherds.
