# Supplementary material for: Creating a brand. Genesis, transformation and diffusion of Qurayyah Painted Ware
Source: PLoS One. 2026 Aug 19;21(8):e0351296. doi: 10.1371/journal.pone.0351296 (PMC13489407; doi:10.1371/journal.pone.0351296)
Supplement: Supplementary file 3 [file pone.0351296.s003.pdf]

# CREATING A BRAND.
# GENESIS, TRANSFORMATION AND DIFFUSION OF QURAYYAH PAINTED WARE

Luciani M. – Fragnoli P. – Gilstrap W.D. – Sterba J.H. – Klainscek D.– Greene J.A. – Bourke S. – Donnelly P.

Corresponding author: Marta Luciani, University of Vienna, marta.luciani@univie.ac.at

……………………………………………………………………………………………………………………………………………………………………….

## SUPPORTING INFORMATION 3

Macrofabrics catalogue from Qurayyah

All drawings and photos: rights holder Marta Luciani

| Macro-fabric* | Catalogue sherd no. | Clay | Main inclusions | Fabric colour | Firing | Surface out | Surface in | Surface colour out | Surface colour in | Produc-tion** | Fabric photo |
|---|---|---|---|---|---|---|---|---|---|---|---|
| F00.01 | QU.M.857.2 | Homogenous, non-porous | Grey, medium, moderate<br>White, small, rare | 7.5YR 6/4 (light brown) to 10YR 5/1 (grey) | NH | sl, sm | sl, non-sm | 10R 5/2 (weak red) | 5YR 5/6 (yellowish red) | HM | 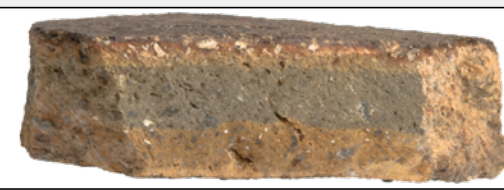 |
| F01.05 | QU.B.61.3 | Homogenous, porous | White, medium, frequent,<br>Red, medium, frequent<br>Yellow, small, moderate<br>Quartz, small moderate | 10R 4/4 (weak red) | H | sl, sm | no sl, non-sm | 10R 5/4 (weak red) | 10R 6/3 (pale red) | HM | 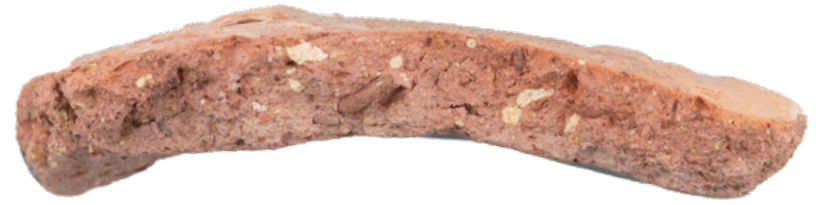 |
| | | | | | | | | | | | |
| F02.01 | QU.A.298.27 | Homogenous, compact | Red, small, rare | 7.5YR 8/1 (white) | H | sl, sm | s-sl, sm | 5YR 5/6 (reddish yellow) | 7.5YR 8/2 (pinkish white) | W-C? | 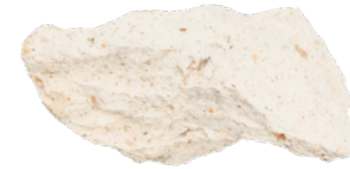 |
| F02.02 | QU.A.287.1 | Homogenous, non-porous | Red, small, rare | 10YR 8/1 (white) | H | s-sl, sm | s-sl, sm | 2.5Y 8/3 (pale yellow) | 10YR 8/2 (very pale brown) | W-C | 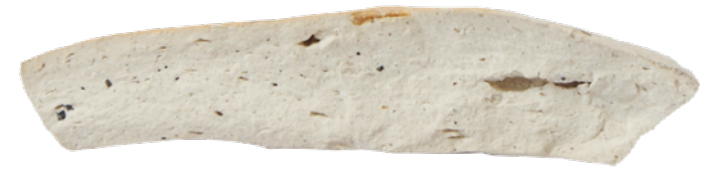 |
| F02.03 | QU.S.9001.81 | Homogenous, non-porous | Black, small, rare | 5YR 6/6 (reddish yellow) | H | sl, sm | sl, sm | 10YR 8/3 (very pale brown) | 10YR 8/2 (very pale brown) | W-C? | 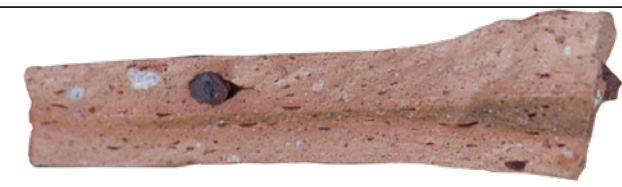 |
| F02.04 | QU.S.9001.91 | Homogenous, non-porous | Red, medium, rare | 5YR 8/2 (pinkish white) | H | s-sl, sm | s-sl, non-sm | 7.5YR 8/3 (pink) | 7.5YR 8/2 (pinkish white) | W-C | 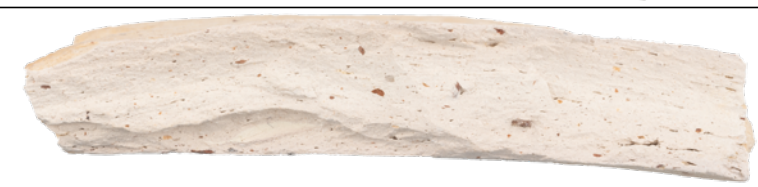 |
| F02.05 | QU.S.9005.1 | Homogenous, compact | White, medium, rare,<br>White, small, moderate | 7.5YR 5/3 (brown) | H | sl, sm | sl, sm | 7.5YR 5/1 (grey) | 7.5YR 4/1 (dark grey) | W-C? | 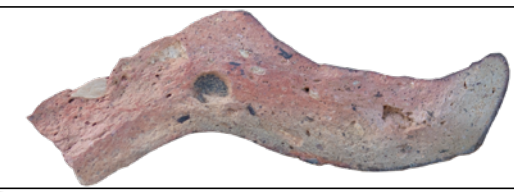 |
| F02.07 | QU.S.9001.66 | Homogenous, compact | Red, small, frequent | 7.5YR 8/3 (pink) | H | sl, sm | sl, sm | 5YR 7/8 (reddish yellow) | 10YR 8/4 (very pale brown) | W-C | 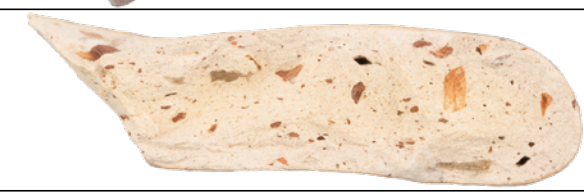 |
| F02.08 | QU.S.9001. 39 | Homogenous, non-porous | Red, small frequent<br>White, small, rare | 10YR 7/6 (yellow) | H | sl, sm | sl, sm | 10YR 5/3 (brown) | 7.5YR 5.2 (brown) | W-C | 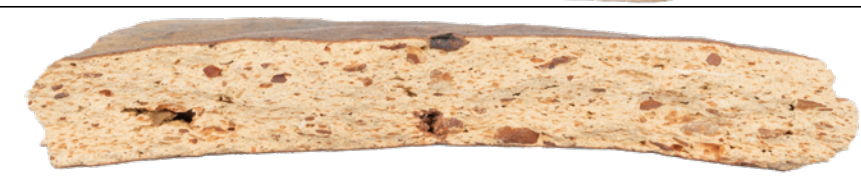 |
| F02.09 | QU.S.9003.26 | Homogenous, slightly porous | Black, medium, frequent<br>Red, medium, rare to moderate | 10YR 8/2 (very pale brown) | H | sl, sm | sl, sm | 10YR 8/4 (very pale brown) | 10YR 8/6 (yellow) | W-C | 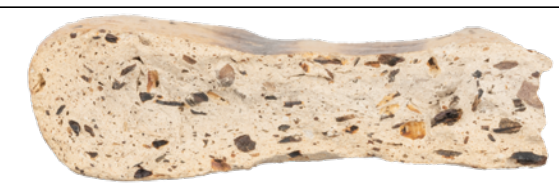 |
| F02.10 | QU.S.9001.85 | Homogenous, slightly porous | Red, medium, frequent<br>White, medium, very frequent | 2.5YR 6/4 (light reddish brown) | H | sl, sm | s-sl, sm | 5YR 5/4 (reddish brown) | 5YR 4/1 (dark grey) | W-C | 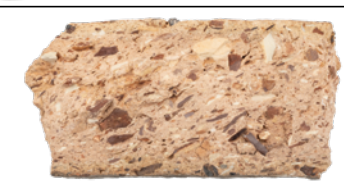 |
| F02.11 | QU.S.9001.6 | Homogenous, slightly porous | Black, medium, frequent<br>Red, medium, frequent | 7.5YR 5/8 (strong brown) | H | sl, sm | sl, sm | 5YR 5/8 (yellowish red) | 5YR 5/6 (red) | W-C | 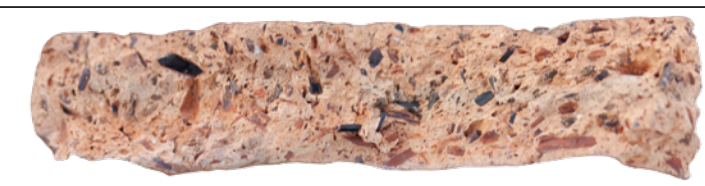 |
| F02.12 | QU.A.7.1 | Homogenous, non-porous | White, medium, moderate<br>Black/grey, small, moderate | 2.5YR 4/4 (reddish brown) | H | no sl, sm | sl, sm | 2.5YR 5/3 (reddish brown) | 2.5 YR 4/6 (red) | W-C | 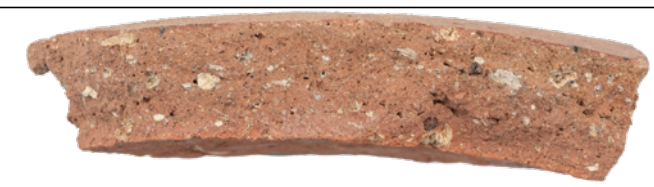 |
| F02.13 | QU.K.702.86 | Homogenous, slightly porous | White, medium frequent<br>Red, medium, rare<br>Black, medium, rare | 10YR 8/6 (yellow) | H | sl, sm | sl, sm | 10YR 6/6 (brownish yellow) | 10YR 7/4 (very pale brown) | W-C? | 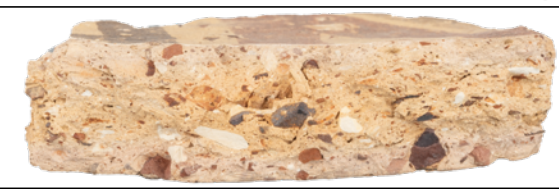 |

| Macro-fabric* | Catalogue sherd no. | Clay | Main inclusions | Fabric colour | Firing | Surface out | Surface in | Surface colour out | Surface colour in | Production** | Fabric photo |
|---|---|---|---|---|---|---|---|---|---|---|---|
| F02.14 | QU.A.22.8 | Homogenous, compact | White, medium, frequent<br>Black, medium, frequent<br>Red, medium, rare | 2.5YR 5/4 (reddish brown) | H | sl, sm | sl, sm | 2.5YR 4/3 (reddish brown) | 2.5YR 4/4 (reddish brown) | W-C | 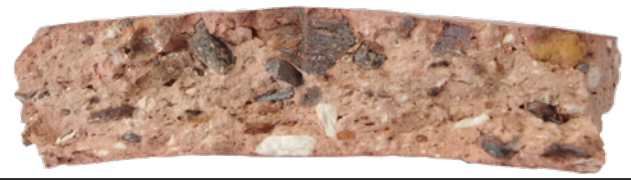 |
| F02.15 | QU.A.22.7 | Homogenous, non-porous | Black, small, frequent<br>White, medium, moderate<br>Red medium, rare | 7.5YR 5/1 (grey) | NH | sl, sm | sl, sm | 5YR 3/1 (very dark grey) | 7.5YR 4/1 (dark grey) | W-C | 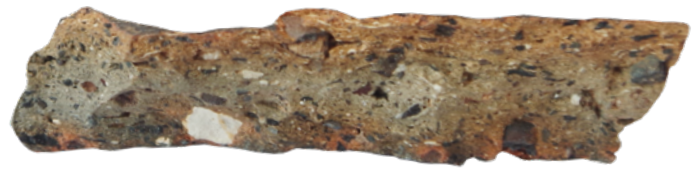 |
| F02.16 | QU.S.9025.1 | Homogenous, compact | Red, medium, frequent<br>White, medium, moderate<br>Black, small, rare | 10R 7/4 (pale red) | H | no sl, sm | no sl, sm | 5YR 5/4 (reddish brown) | 5YR 6/4 (reddish brown) | W-T? | 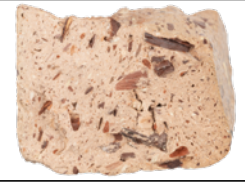 |
| F02.17 | QU.K.702.103 + QU.K.702.123 | Homogenous, non-porous | Red, small, moderate<br>Black, small, moderate<br>White, small, rare | 5YR 6/6 (reddish yellow) | H | sl, sm | sl, sm | 7.5YR 7/4 (pink) | 10YR 8/4 (very pale brown) | W-C? | 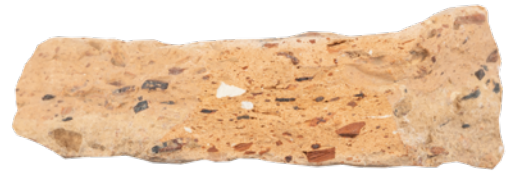 |
| F02.18 | QU.A.505/36.7 | Homogenous, compact | White, medium, very frequent<br>Red, medium, rare<br>Black, small, rare<br>Grey, small, moderate | 2.5YR 6/3 (light reddish brown) | H | sl, sm | sl, sm | 2.5YR 4/3 (reddish brown) | 2.5YR 4/4 (reddish brown) | W-C | 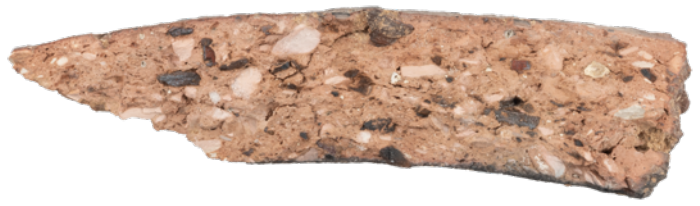 |
| F02.19 | QU.A.647.33 | Homogenous, compact | White, small, moderate<br>Black, small, moderate<br>Red, small, rare | 2.5YR 6/8 (light red) | H | sl, sm | sl, sm | 10R 4/6 (red) | 10R 4/6 (red) | W-C | 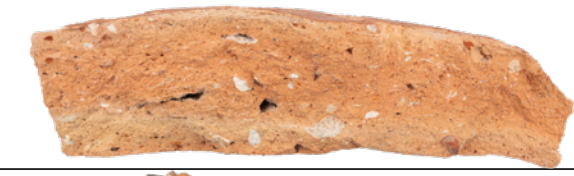 |
| F02.20 | QU.A.23.1 | Homogenous, compact | Black, medium/big, very frequent<br>White, medium, moderate | 7.5YR 5/1 (grey) | H | sl, coarse | sl, coarse | 2.5YR 4/2 (weak red) | 5YR 3/2 (dark reddish brown) | W-C | 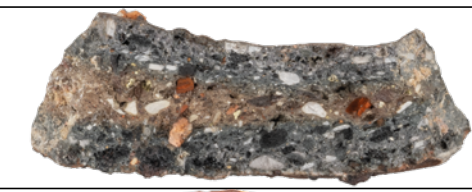 |
| F02.21 | QU.A.637.8 | Homogenous, non-porous | Black, medium, frequent<br>White, medium, frequent | 10YR 8/6 (yellow) | H | sl, sm | sl, sm | 2.5YR 5/4 (reddish brown) | 2.5YR 7/6 (light red) | W-C | 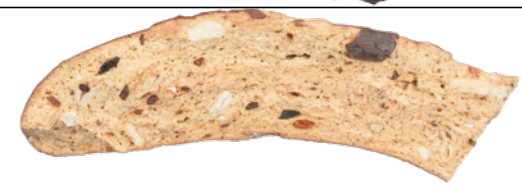 |

*Macrofabrics have been labeled with progressive numbers starting from F02.01 for finest to F02.21 for coarsest (except F02.06 and F02.17 which are Iron Age and final LBA macrofabrics).

**More comprehensive reconstructions of the entire *chaîne opératoire* are planned for future research.

**Abbreviation key**

| | | | |
|---|---|---|---|
| *Firing:* | H | › | homogeneous |
| | NH | › | non-homogeneous |
| *Surface:* | sl | › | slip |
| | s-sl | › | self-slip |
| | no sl | › | no slip |
| | sm | › | smoothed |
| | non-sm | › | non-smoothed |
| | un. | › | untreated |
| *Production:* | HM | › | hand-made |
| | W-C | › | wheel-coiled |
| | W-T | › | wheel-thrown |

**Fabric composition criteria**

| | | | |
|---|---|---|---|
| *Clay:* | Homogeneity | › | homogeneous or non-homogeneous |
| | Porosity | › | compact (<2%), non-porous (2%-5%), slightly porous (5%-10%), porous (>10%) |
| *Main inclusions:* | Colour | › | mainly red, black, white |
| | Size | › | small (<1mm), medium (1-2mm), large (>2mm) |
| | Amount | › | rare (<2%), moderate (2%-5%), frequent (5%-10%), very frequent (>10%) |
