# Supplementary material for: Creating a brand. Genesis, transformation and diffusion of Qurayyah Painted Ware
Source: PLoS One. 2026 Aug 19;21(8):e0351296. doi: 10.1371/journal.pone.0351296 (PMC13489407; doi:10.1371/journal.pone.0351296)
Supplement: Supplementary file 4 [file pone.0351296.s004.pdf]

CREATING A BRAND.
GENESIS, TRANSFORMATION AND DIFFUSION OF QURAYYAH PAINTED WARE

Luciani M. – Fragnoli P. – Gilstrap W.D. – Sterba J.H. – Klainscek D.– Greene J.A. – Bourke S. – Donnelly P.

Corresponding author: Marta Luciani, University of Vienna, marta.luciani@univie.ac.at

SUPPORTING INFORMATION 4

Plans and sections of the pottery kiln (Phases 5, 4 and 3) excavated in Area A in Qurayyah

As visible below, in each of the three phases the construction was modified, going from a downdraft furnace with external chimney (Phase 5) to an inner re-plastering, which sealed the rear chimney (Phase 4) to a final refurbishing through the addition of an internal mudbrick pillar to sustain the weight of the pierced floor bearing the vessels (Phase 3). In both of the last two (4 and 3) phases, the furnace seems to have functioned as updraft kiln.

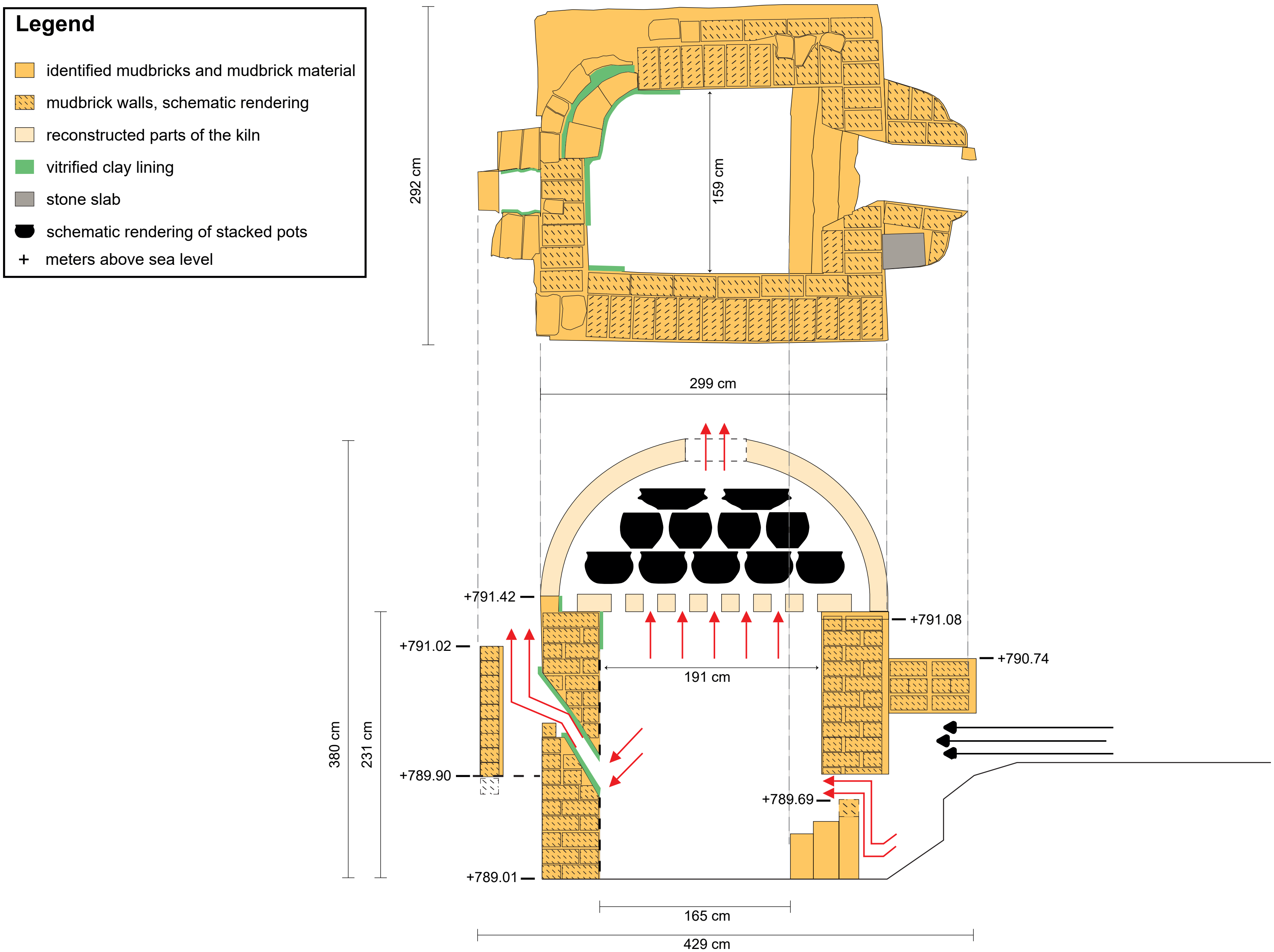

Qurayyah

Area A, Phase 5

Joint Saudi-Arabian - Austrian Archaeological Project

Plan and section of downdraft kiln

Scale: 1:50
Concept: M. Luciani
QGIS: F. Spitaler
Illustrator: D. Klainscek

0 1 2 m N

Local Coordinate System - based on UTM 37 North WGS 84: Base = N 3177557.80; E 198080.79
Altitude (A)MSL
2025/05/08

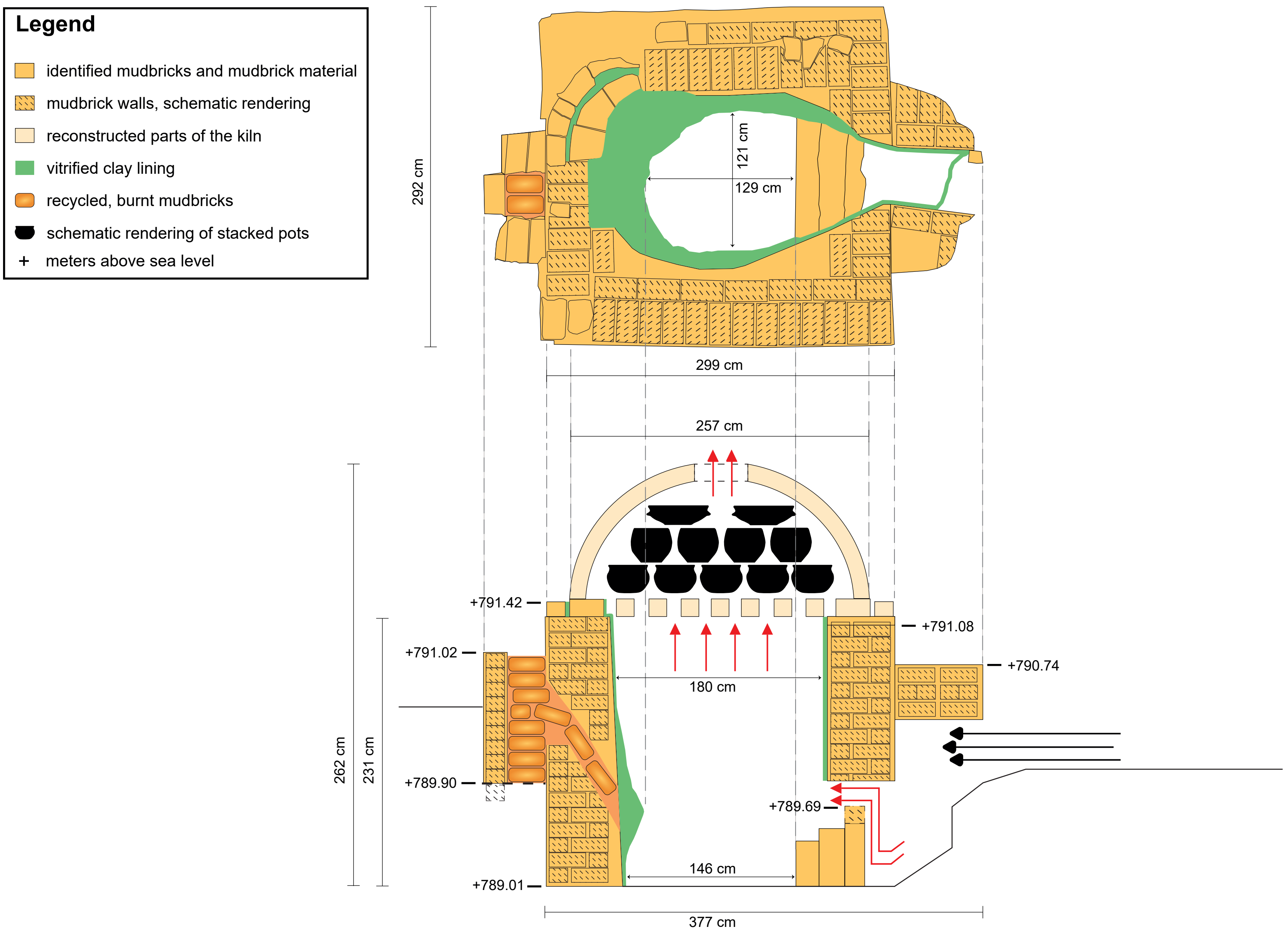

Qurayyah

Area A, Phase 4

Joint Saudi-Arabian - Austrian Archaeological Project

Plan and section of updraft kiln

Scale: 1:50
Concept: M. Luciani
QGIS: F. Spitaler
Illustrator: D. Klainsceck

0 1 2 m

N

Local Coordinate System - based on UTM 37 North WGS 84: Base = N 3177557.80; E 198080.79
Altitude (A)MSL
2025/05/08

Legend

- identified mudbricks and mudbrick material
- mudbrick walls, schematic rendering
- reconstructed parts of the kiln
- vitrified clay lining
- recycled, burnt mudbricks
- schematic rendering of stacked pots
- ash layer
- \+ meters above sea level

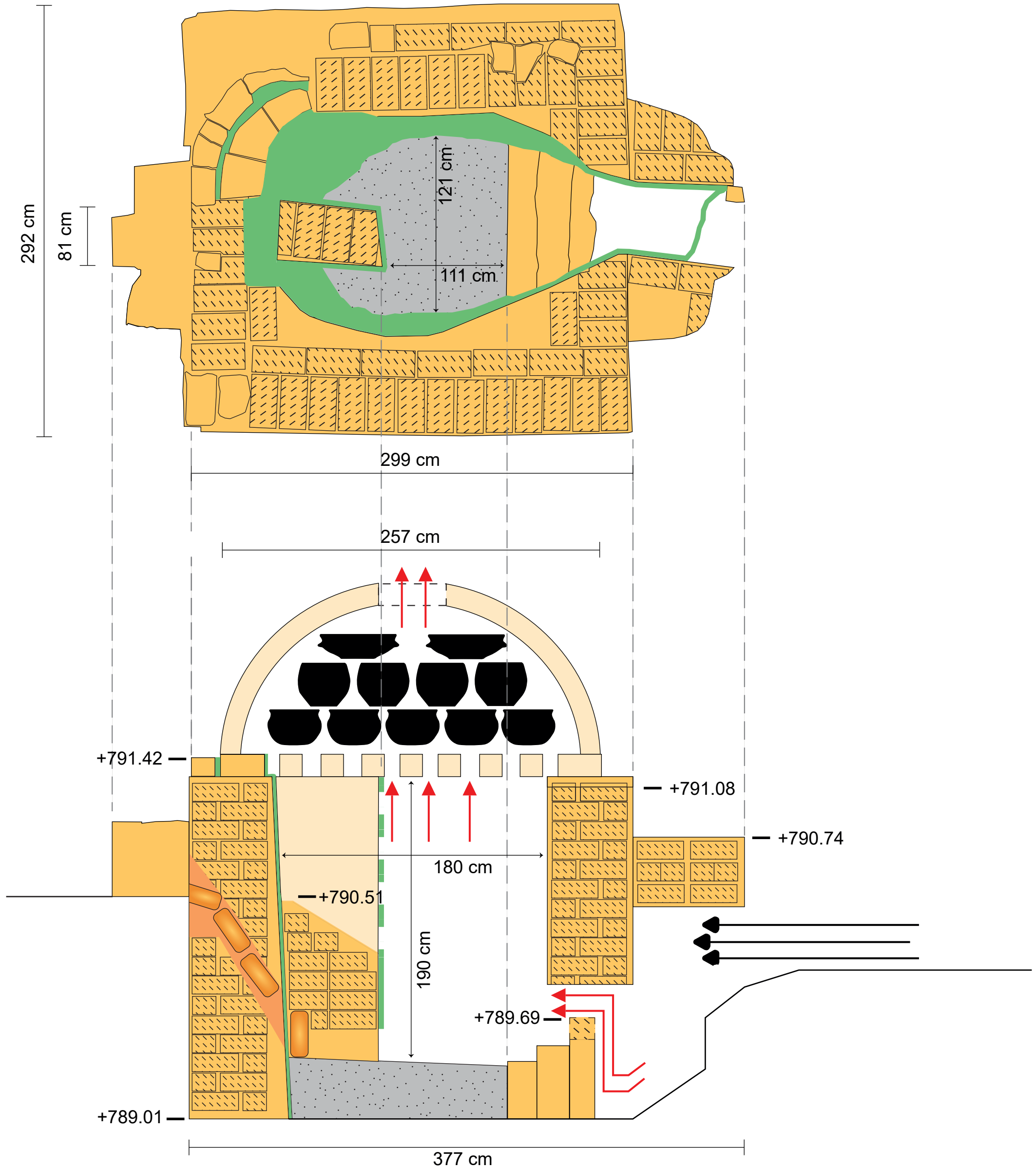

Qurayyah

Area A, Phase 3

Joint Saudi-Arabian - Austrian Archaeological Project

universität wien

هيئة التراث
Heritage Commission

Plan and section of updraft kiln

Scale: 1:50
Concept: M. Luciani
QGIS: F. Spitaler
Illustrator: D. Klainscek

0 1 2 m
N

Local Coordinate System -
based on UTM 37 North WGS 84: Base = N 3177557.80; E 198080.79
Altitude (A)MSL
2025/05/08
