# Supplementary material for: Creating a brand. Genesis, transformation and diffusion of Qurayyah Painted Ware
Source: PLoS One. 2026 Aug 19;21(8):e0351296. doi: 10.1371/journal.pone.0351296 (PMC13489407; doi:10.1371/journal.pone.0351296)
Supplement: Supplementary file 5 [file pone.0351296.s005.pdf]

CREATING A BRAND.
GENESIS, TRANSFORMATION AND DIFFUSION OF QURAYYAH PAINTED WARE

Luciani M. – Fragnoli P. – Gilstrap W.D. – Sterba J.H. – Klainscek D.– Greene J.A. – Bourke S. – Donnelly P.

Corresponding author: Marta Luciani, University of Vienna, marta.luciani@univie.ac.at

....

SUPPORTING INFORMATION 5

Modelled and calibrated ^{14}C dates of organic samples from Qurayyah

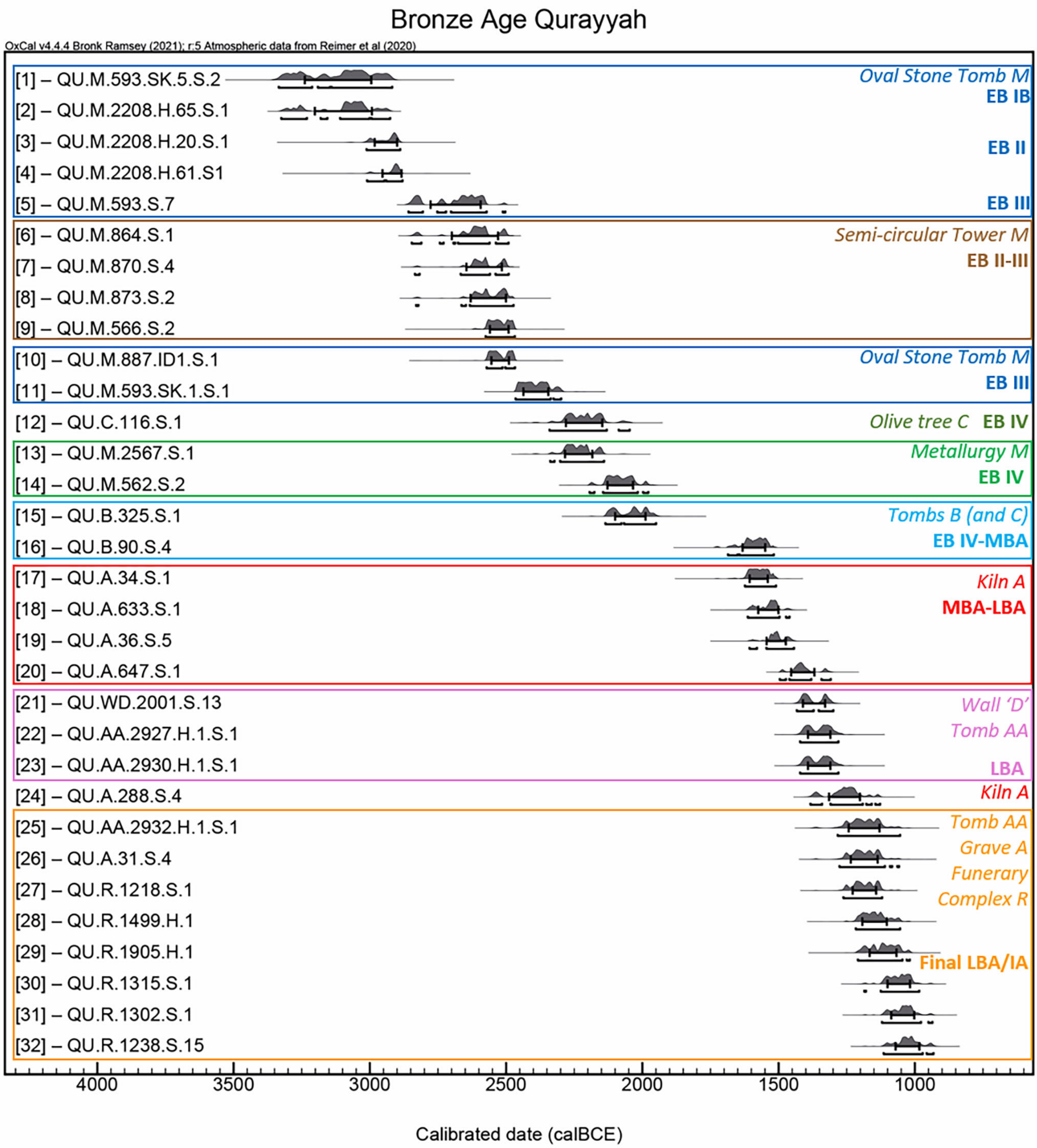

The 14th century cal BCE date (sample no. [24]) obtained from the latest deposits of the kiln, needs most likely to be considered intrusive and stemming from the later, secondary use of the kiln as burial for 20 mostly newborn children (sample no. [26], see Luciani M, Binder M, Alsaud AS. Life and living conditions in north-west Arabia during the Bronze Age: first results from the bioarchaeological work at Qurayyah. Proceedings of the Seminar for Arabian Studies 48, Oxford, 2018. p. 187).
