# Supplementary material for: Creating a brand. Genesis, transformation and diffusion of Qurayyah Painted Ware
Source: PLoS One. 2026 Aug 19;21(8):e0351296. doi: 10.1371/journal.pone.0351296 (PMC13489407; doi:10.1371/journal.pone.0351296)
Supplement: Supplementary file 6 [file pone.0351296.s006.pdf]

CREATING A BRAND.
GENESIS, TRANSFORMATION AND DIFFUSION OF QURAYYAH PAINTED WARE

Luciani M. – Fragnoli P. – Gilstrap W.D. – Sterba J.H. – Klainscek D.– Greene J.A. – Bourke S. – Donnelly P.

Corresponding author: Marta Luciani, University of Vienna, marta.luciani@univie.ac.at

…….

SUPPORTING INFORMATION 6

Detailed descriptions and micrographs of petrogroup Qurayyah 1

All drawings and photos: rights holder Marta Luciani

Petrogroups Description - QURAYYAH

Qurayyah 1a

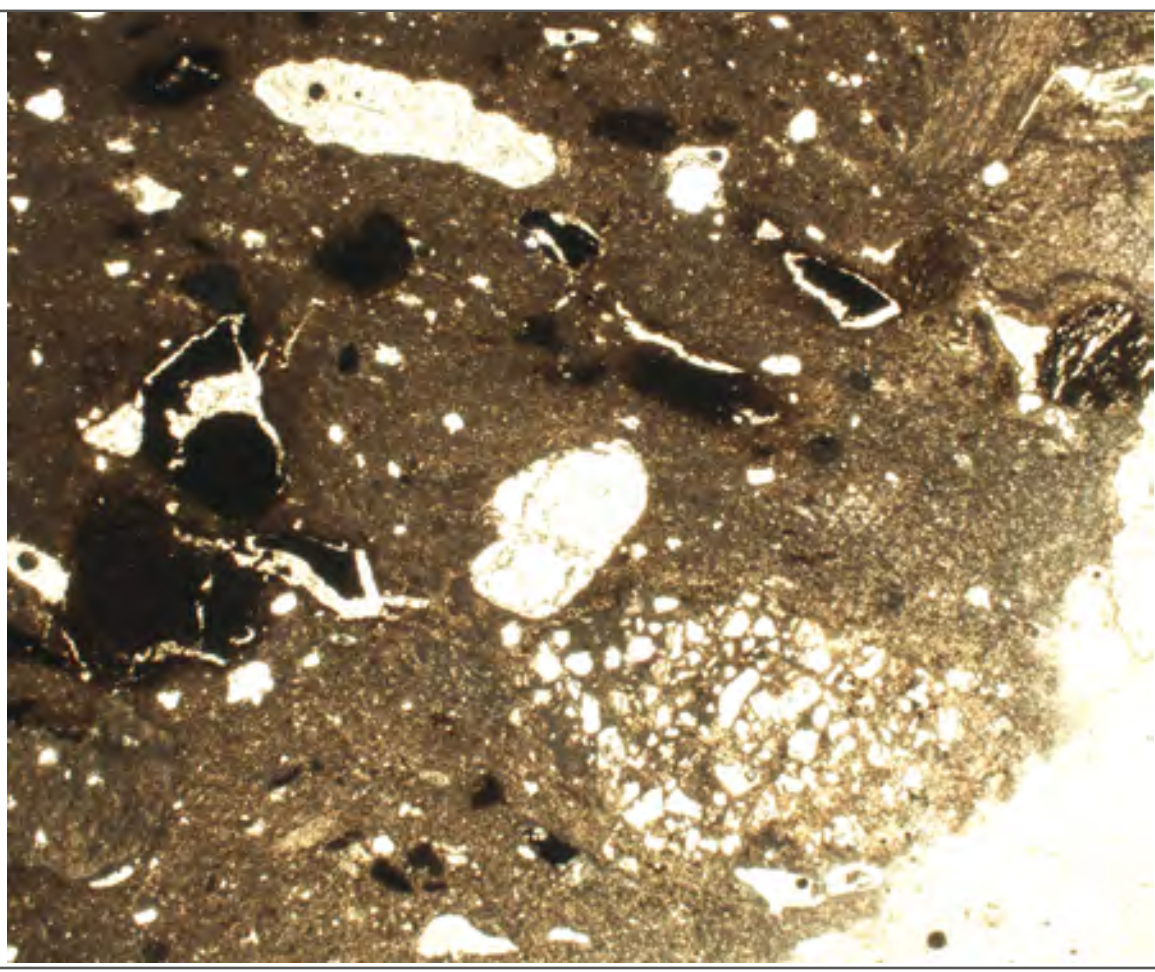
Sample **Q26**: PPL, x2.5, field of view 6.54mm

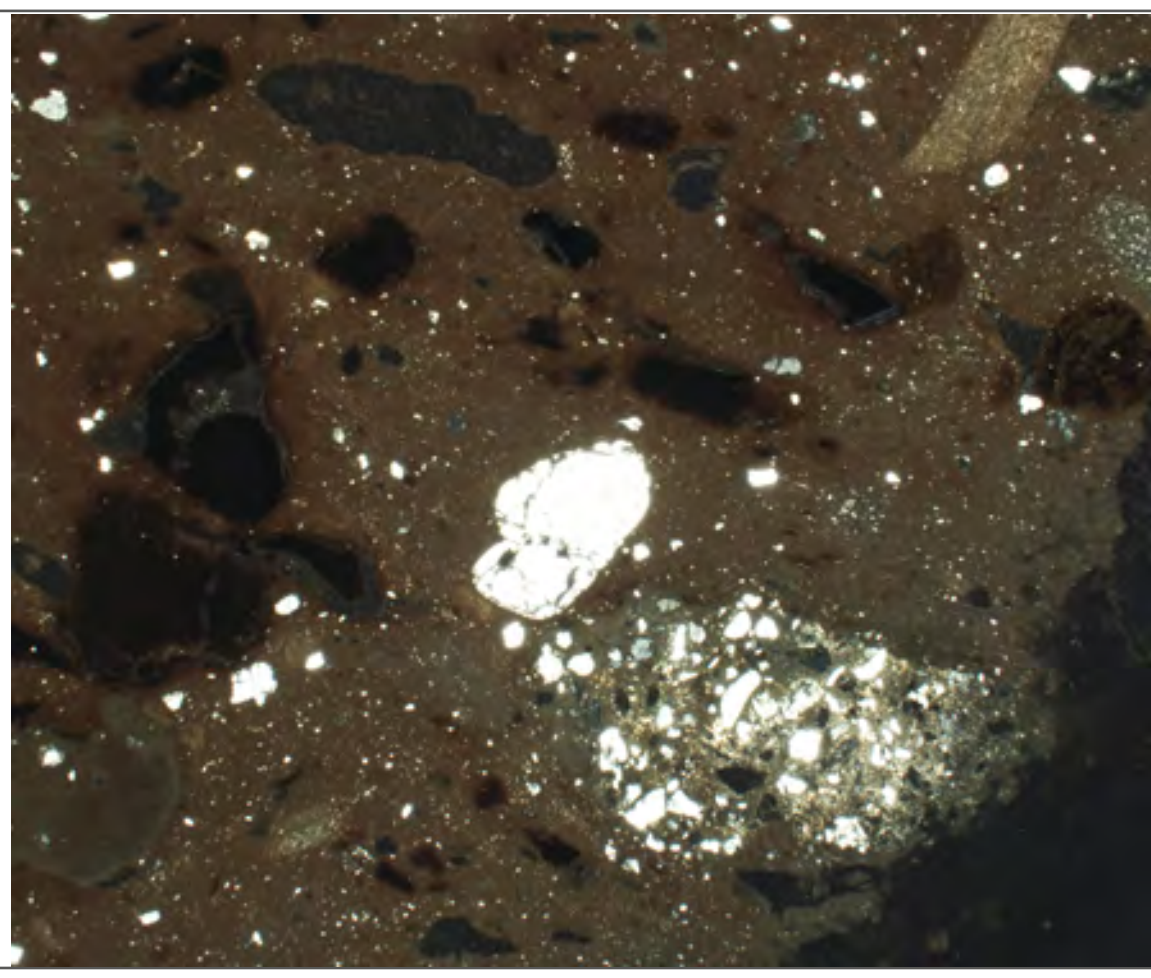
Sample **Q26**: XP, x2.5, field of view 6.54mm

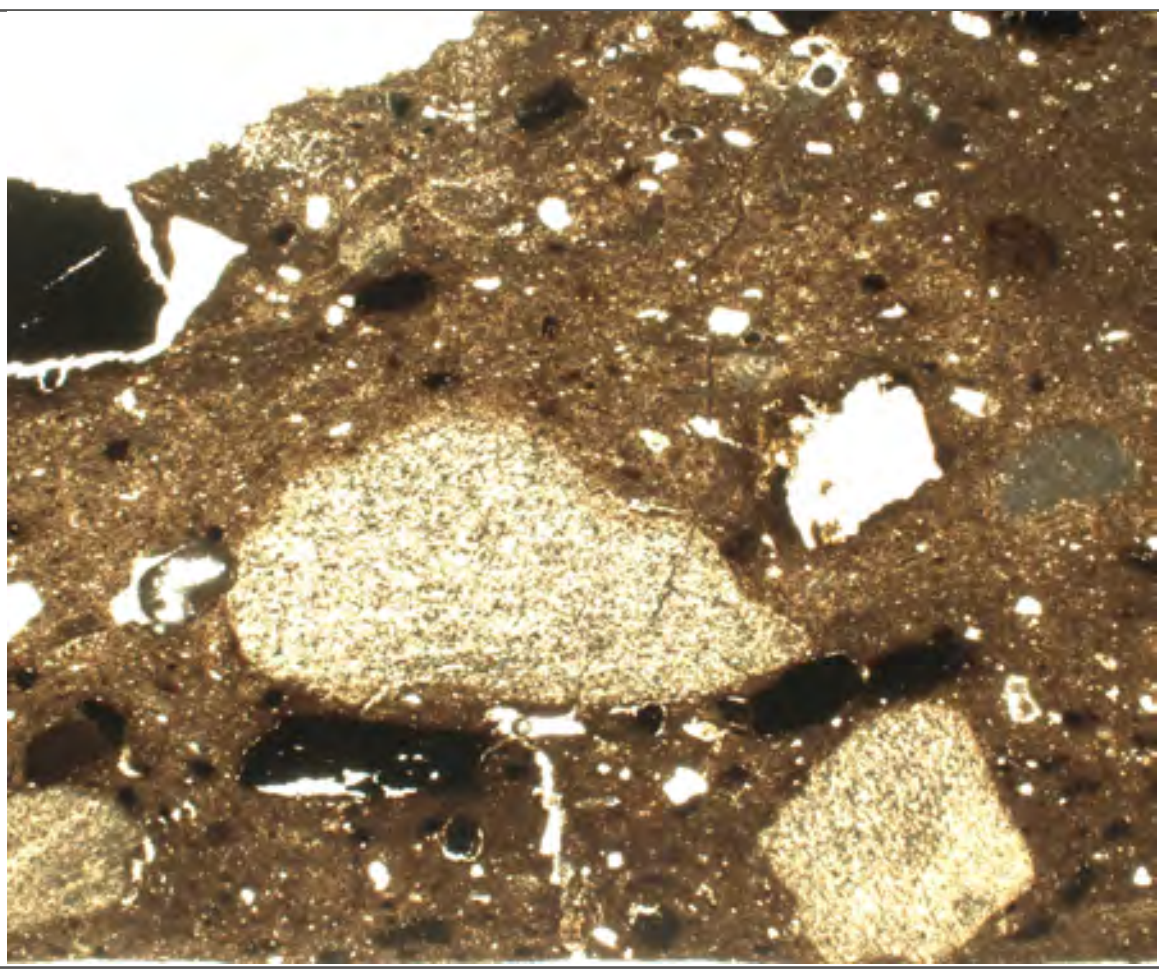
Sample **Q26**: PPL, x2.5, field of view 6.54mm

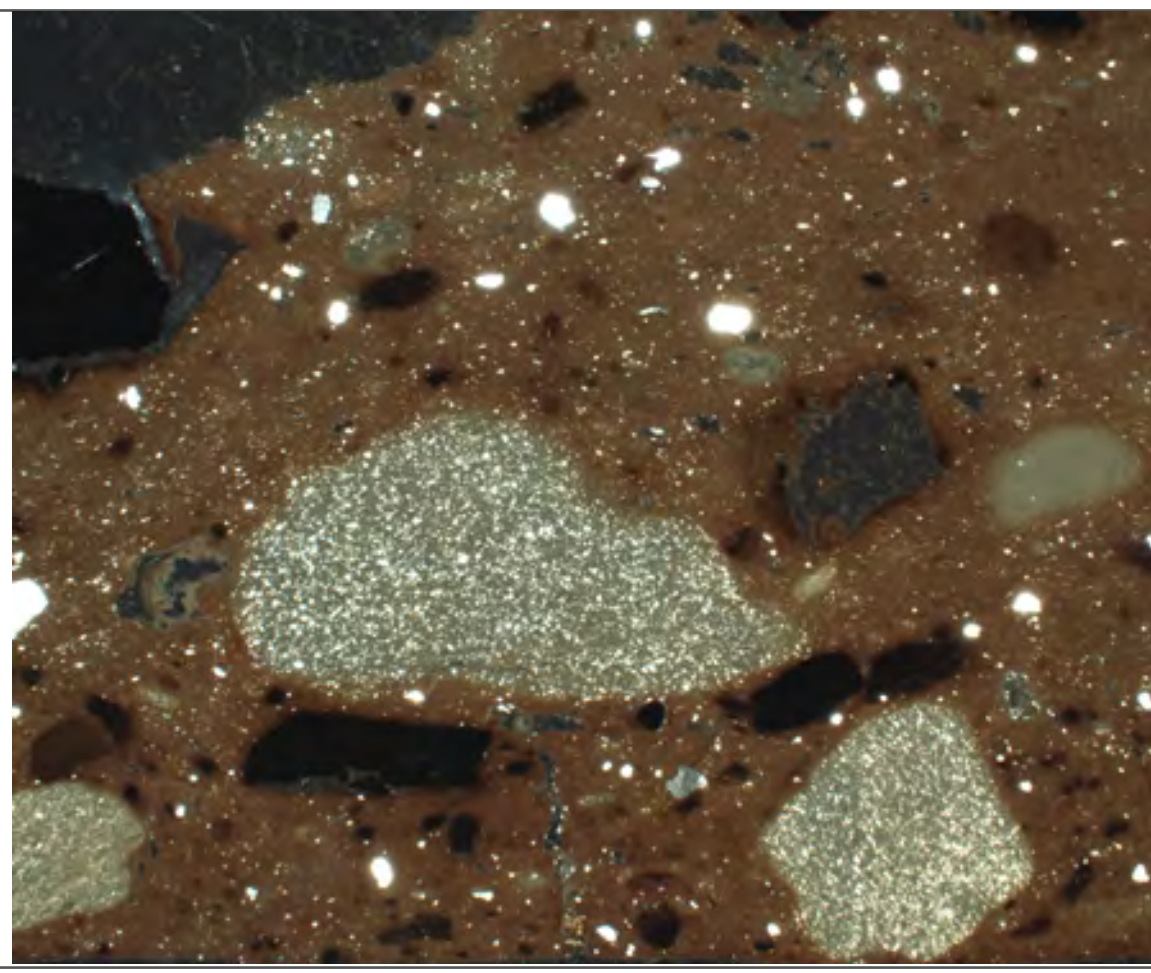
Sample **Q26**: XP, x2.5, field of view 6.54mm

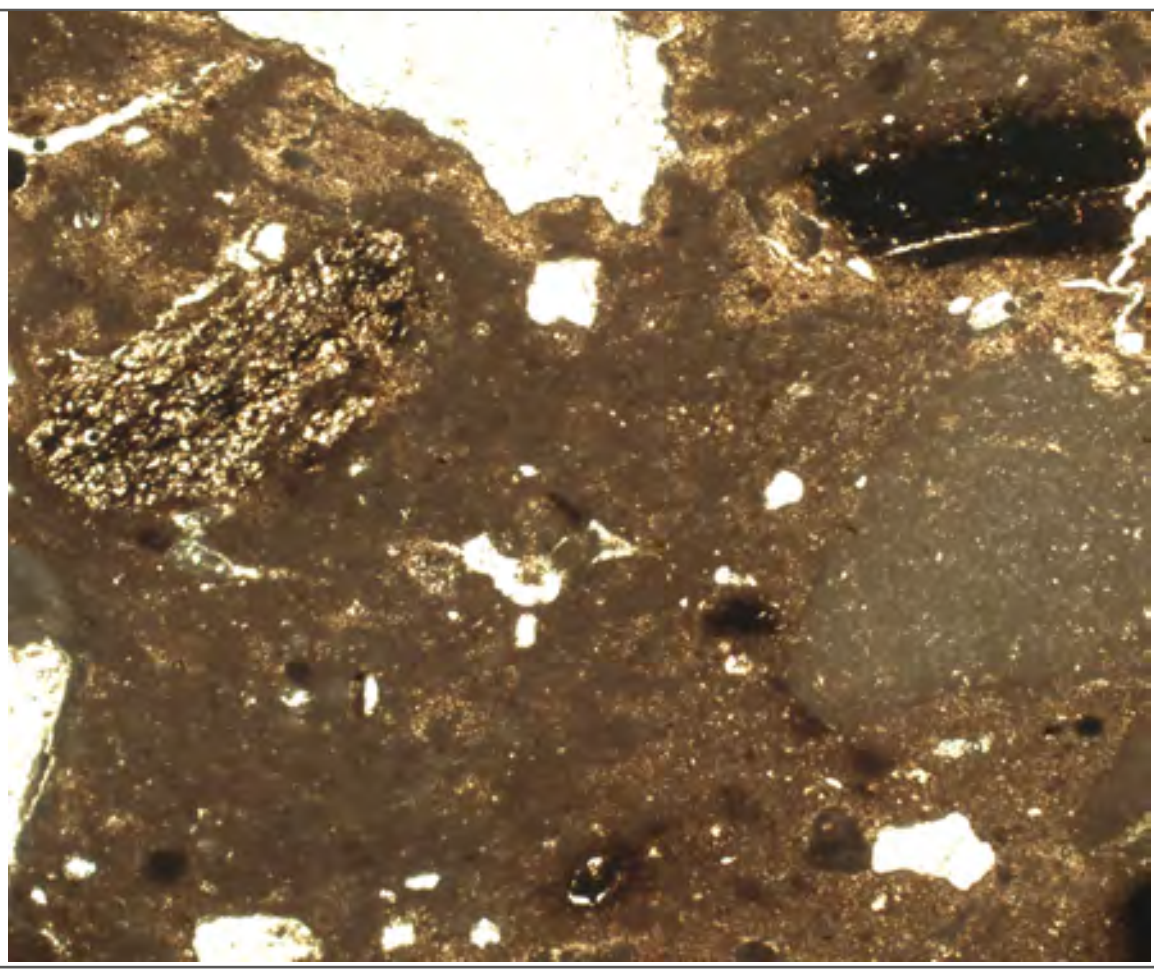
Sample **Q26**: PPL, x5, field of view 3.39mm

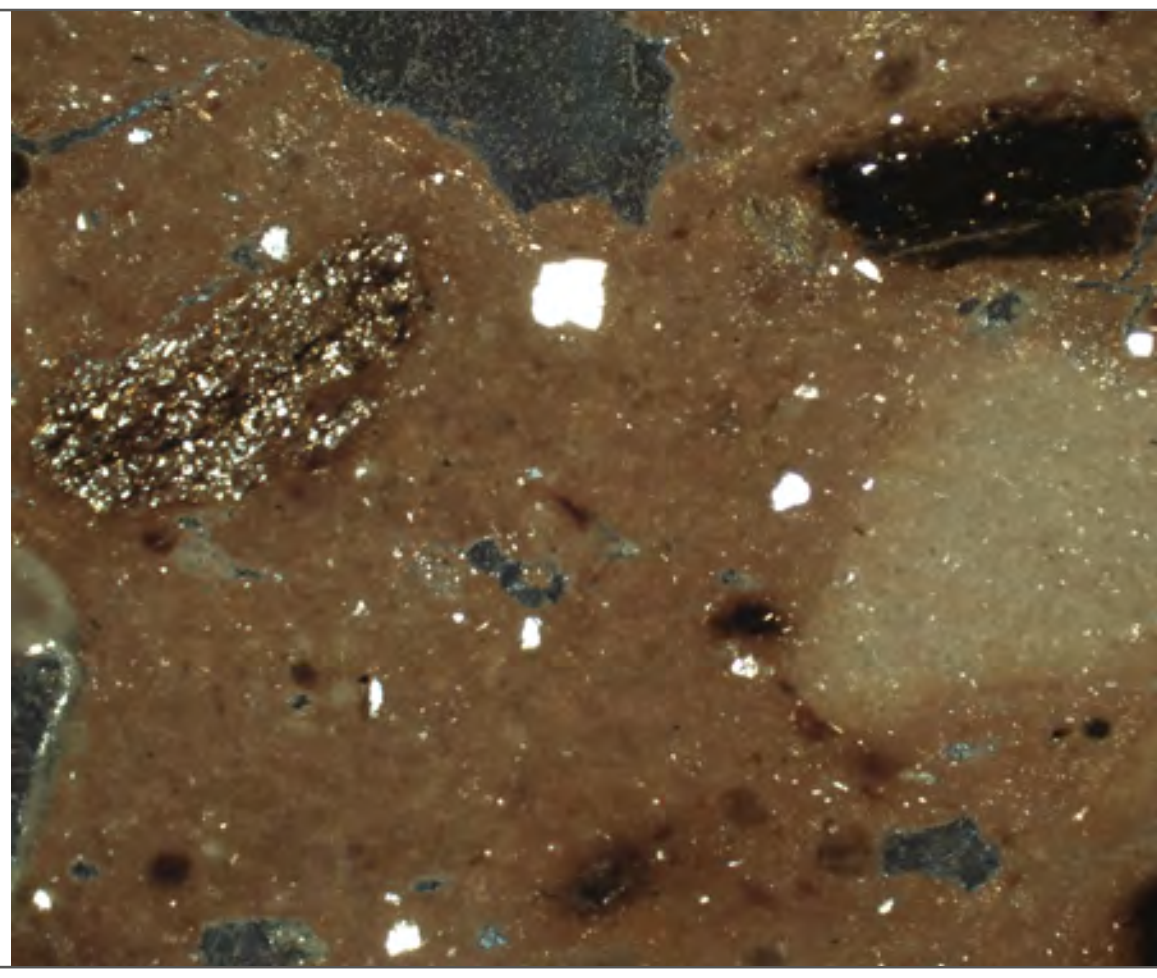
Sample **Q26**: PPL, x5, field of view 3.39mm

Inclusions
Inclusions reach maximum frequencies and sizes of respectively 35% and 5mm, show elongated and angular shapes, are close to double-spaced, slightly to randomly oriented, and feature bi- to polymodal grain-size distributions.

Coarse Fraction
The coarse fraction represents 80% of the total inclusions and shows sizes comprised between 1 and 5mm.

Predominant	›	Shales, elongate, subangular to angular, 1-5mm, mode: 2-3mm
Very few	›	Siltstones, elongate to equant, subrounded to subangular, 1.2-3.36mm, mode: 1.5mm
Very rare to absent	›	Sandstone, elongate, subrounded-subangular, 2.9mm

Fine Frac ion
The fine fraction accounts for 20% with sizes ranging between 0.08 and 1mm.

Predominant	›	Quartz
Predominant to frequent	›	Fe-Oxides
Few	›	Shales
Rare to absent	›	Mica muscovite

Matrix
Matrix accounts for 60%, is non-calcareous and homogeneous in texture and color. Colors XP include golden, yellowish brown and pale to dark reddish-brown. Under PPL colors range from pale greenish-yellowish-brown to dark reddish-brown. The optical activity is generally inactive, with vitrified areas, except for Q38 that features a grano-striated b-fabric texture.

Voids
The porosity accounts for 5% and is primarily composed of meso- to macro vughs and planar voids of elongated and angular shapes. The smallest porosity (micro to meso vughs) might feature equant and subrounded shapes. Voids are slightly to randomly oriented.

Petrogroups Description - QURAYYAH

Qurayyah 1b

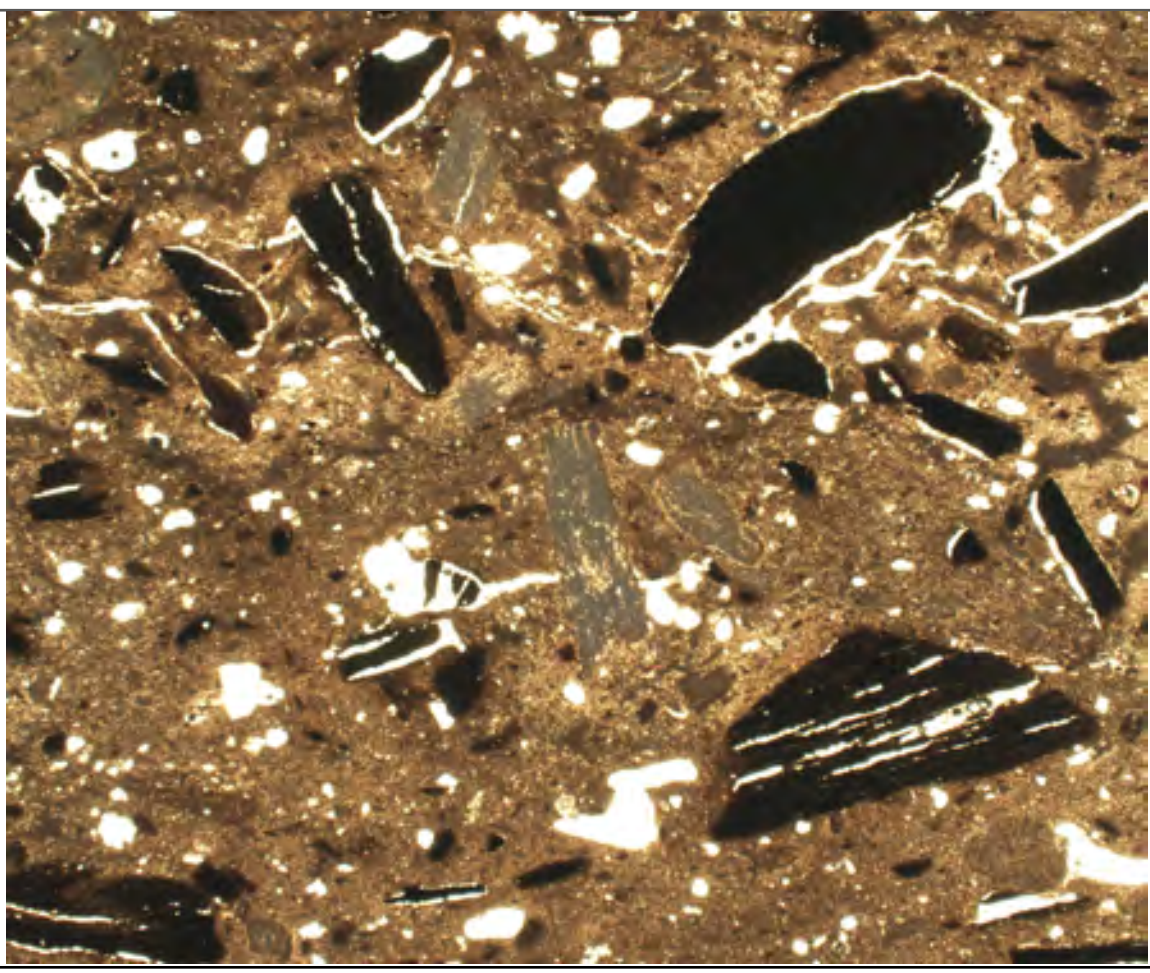

Sample **Q19**: PPL, x2.5, field of view 6.54mm

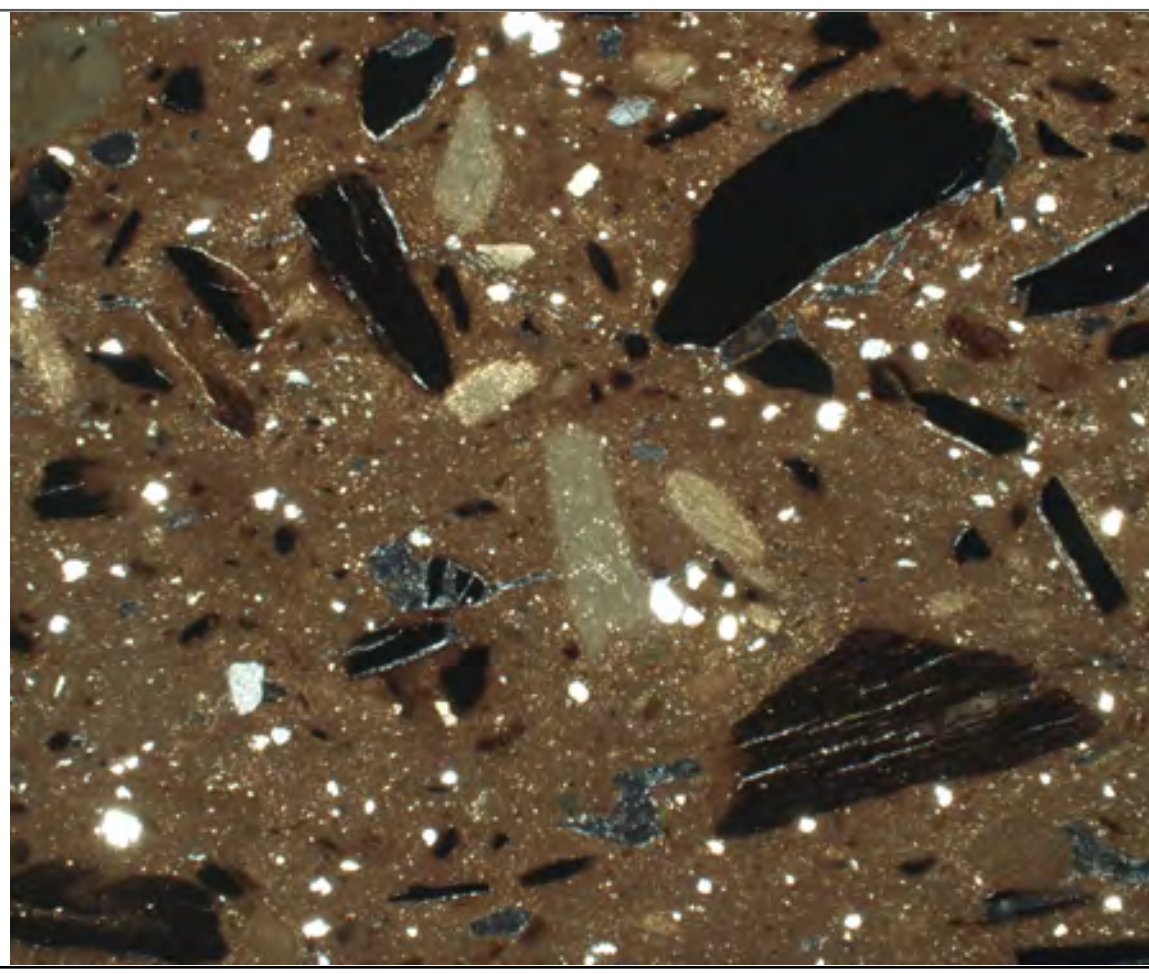

Sample **Q19**: XP, x2.5, field of view 6.54mm

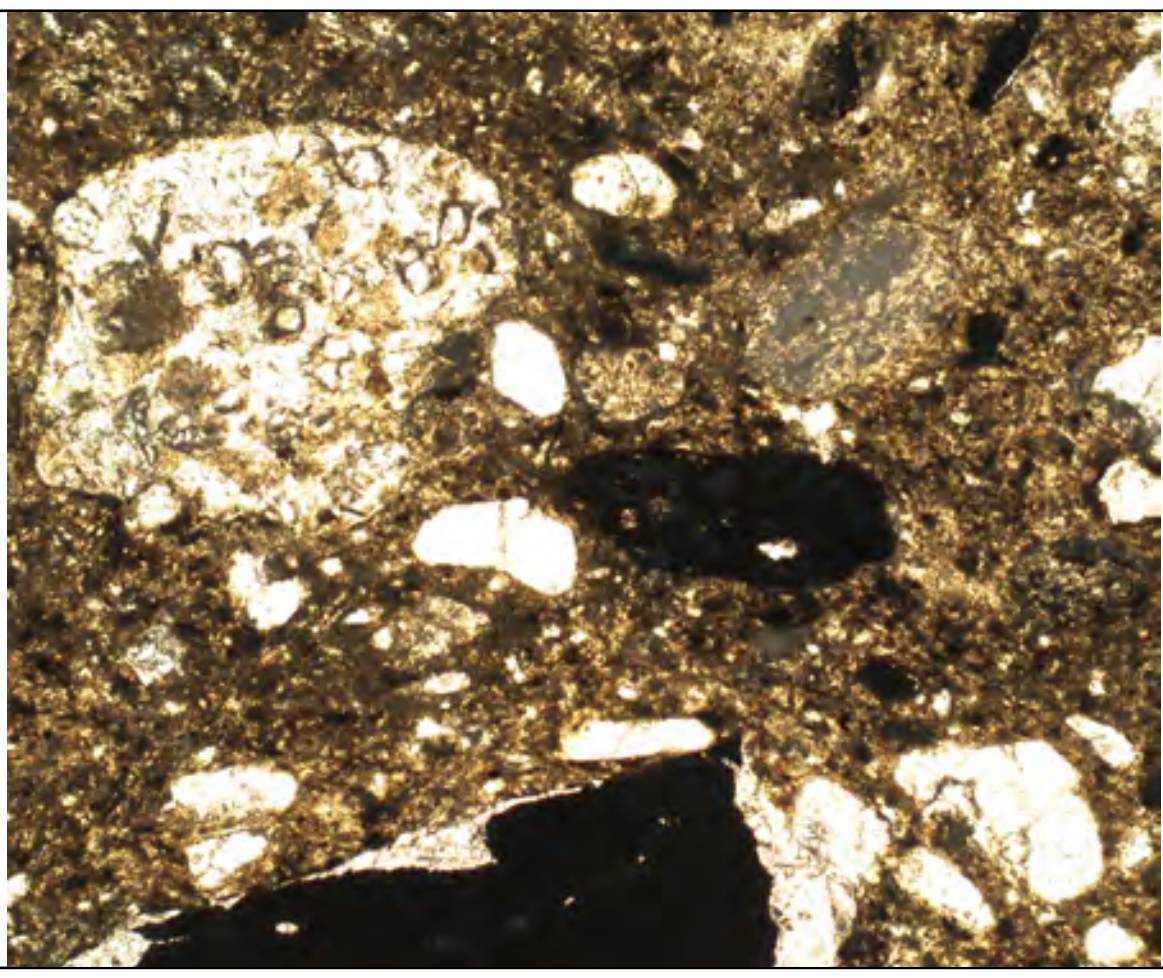

Sample **Q21**: PPL, x10, field of view 1.69mm

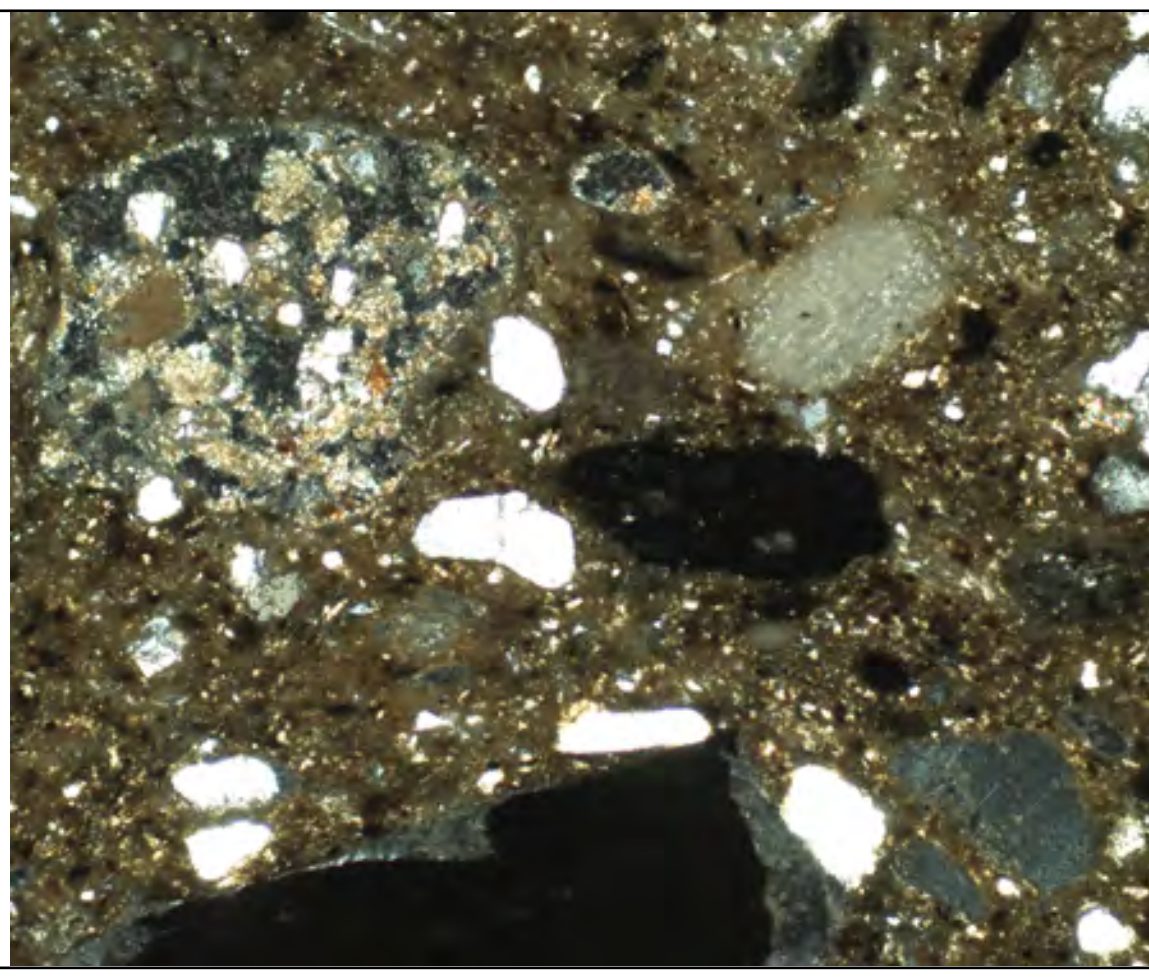

Sample **Q21**: XP, x10, field of view 1.69mm

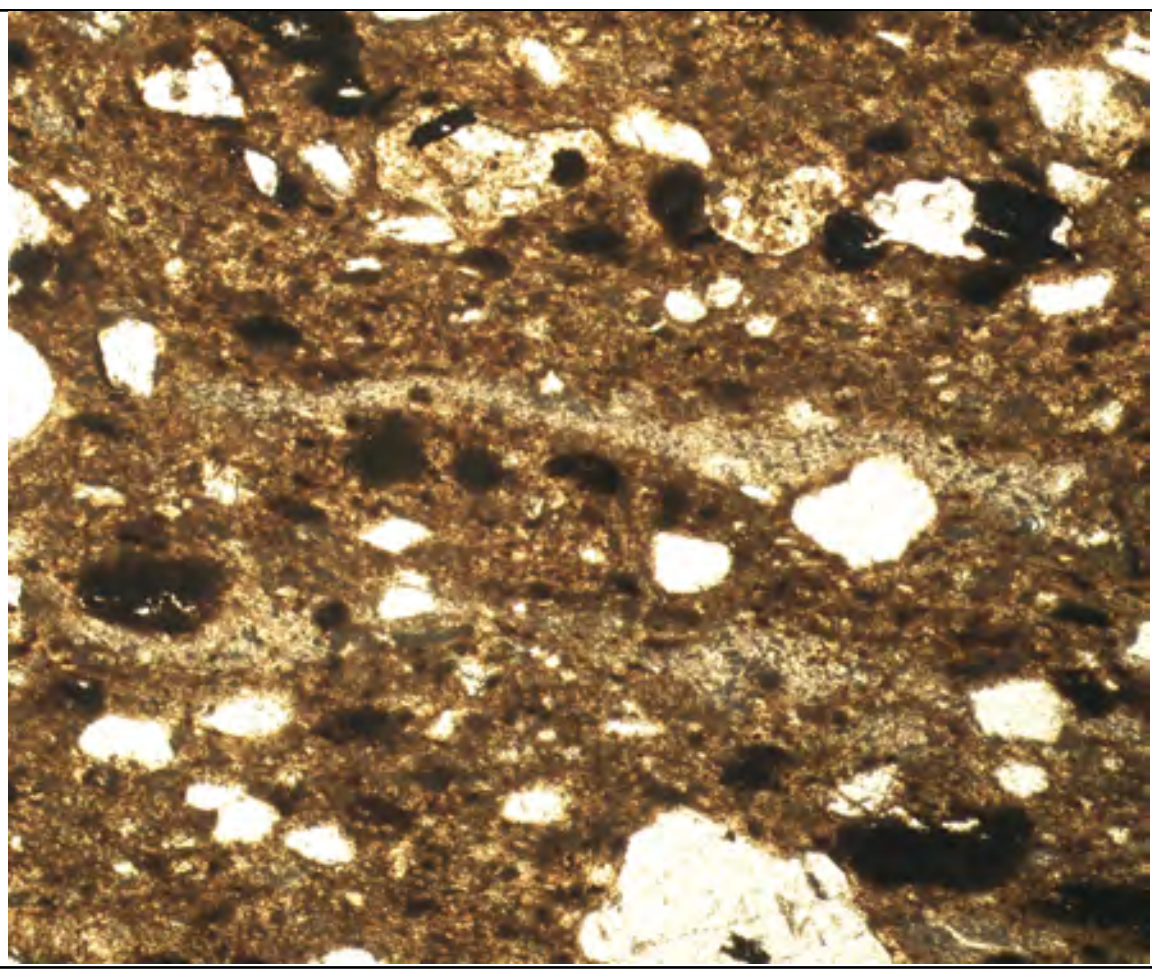

Sample **Q34**: PPL, x10, field of view 1.69mm

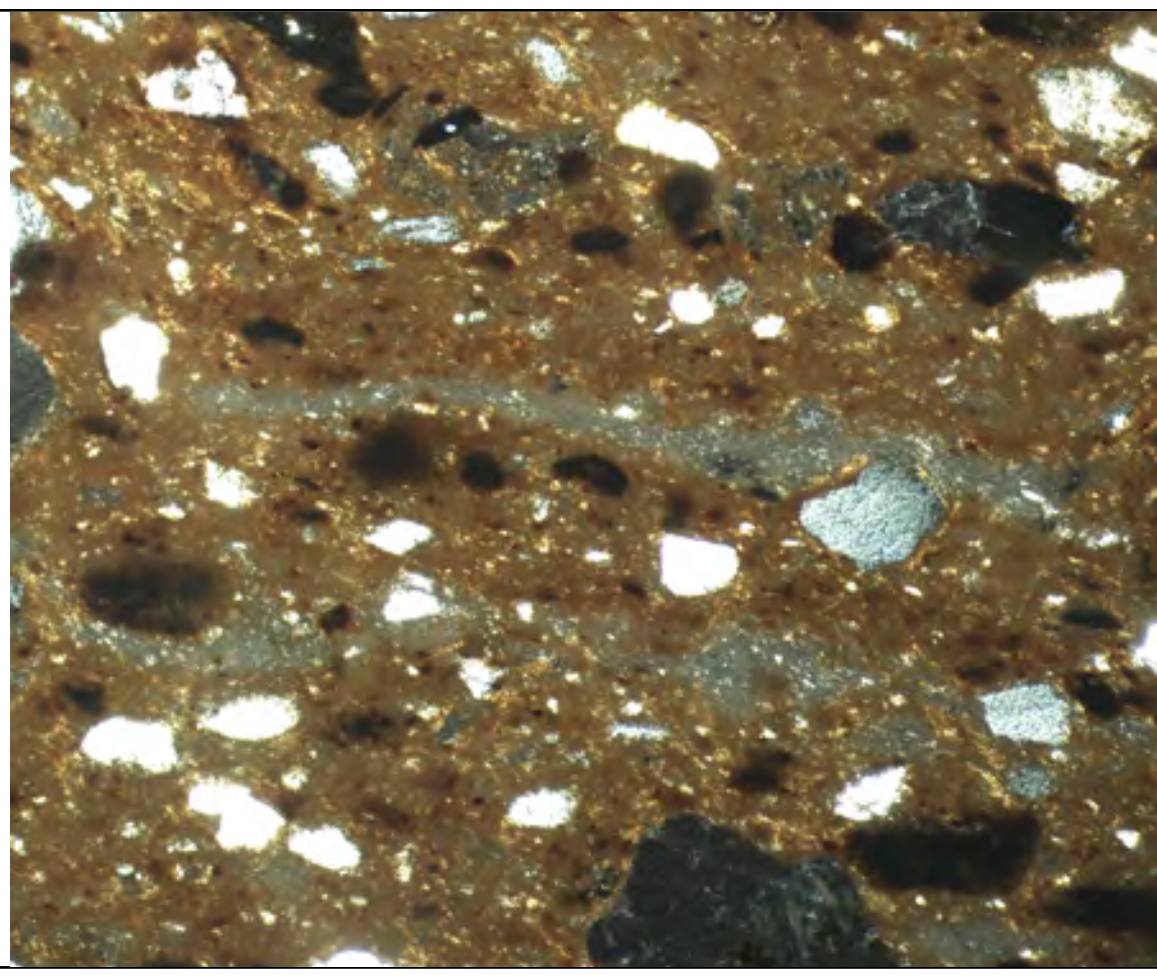

Sample **Q34**: XP, x10, field of view 1.69mm

Inclusions
Inclusions reach maximum frequencies and sizes of respectively 25% and 6.4mm, show elongated and angular shapes, are close to open-spaced, slightly to randomly oriented, and feature bi- to polymodal grain-size distributions.

Coarse Fraction
The coarse fraction represents 60% of the total inclusions and shows sizes comprised between 1 and 6.4mm.

Predominant	›	Shales, elongate, angular, 1-6.4mm, mode: 1-2mm
Very few to absent	›	Siltstones, elongate to equant, subrounded to subangular, 1.6-2.8mm, mode: 1-2mm
Very rare to absent	›	Sandstones, equant to elongate, subangular, 1-1.2mm, mode: 1mm

Fine Fraction
The fine fraction accounts for 40% with sizes ranging between 0.08 and 1mm.

Predominant	›	Quartz
Frequent to few	›	Fe-Oxides
Few	›	Shales
Very few	›	Sandstones
Rare to absent	›	Mica muscovite

Matrix
Same as Qurayyah 1a, but is siltier and accounts for 70%.

Voids
Same as Qurayyah 1a.

Petrogroups Description - QURAYYAH

Qurayyah 1c

<table>
<tr><td>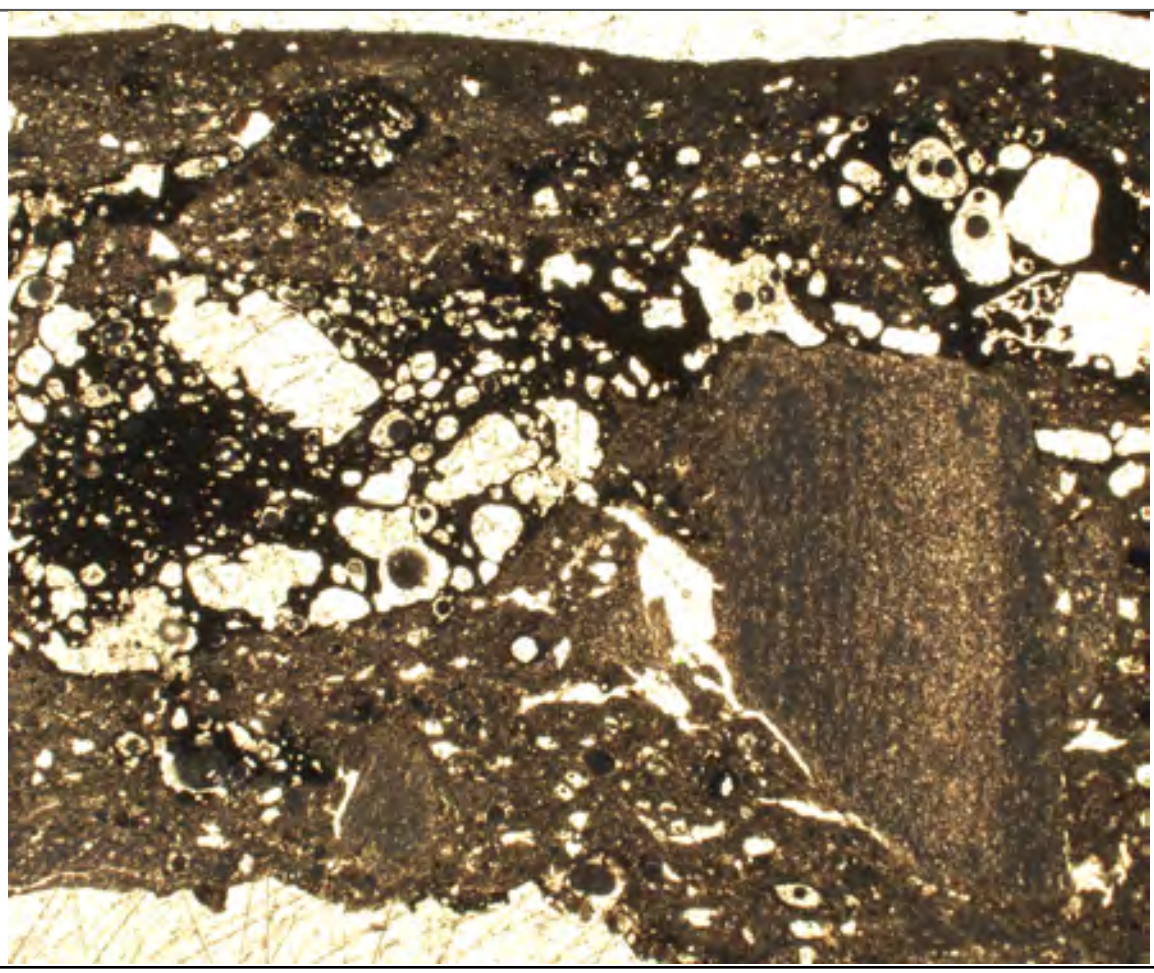</td><td>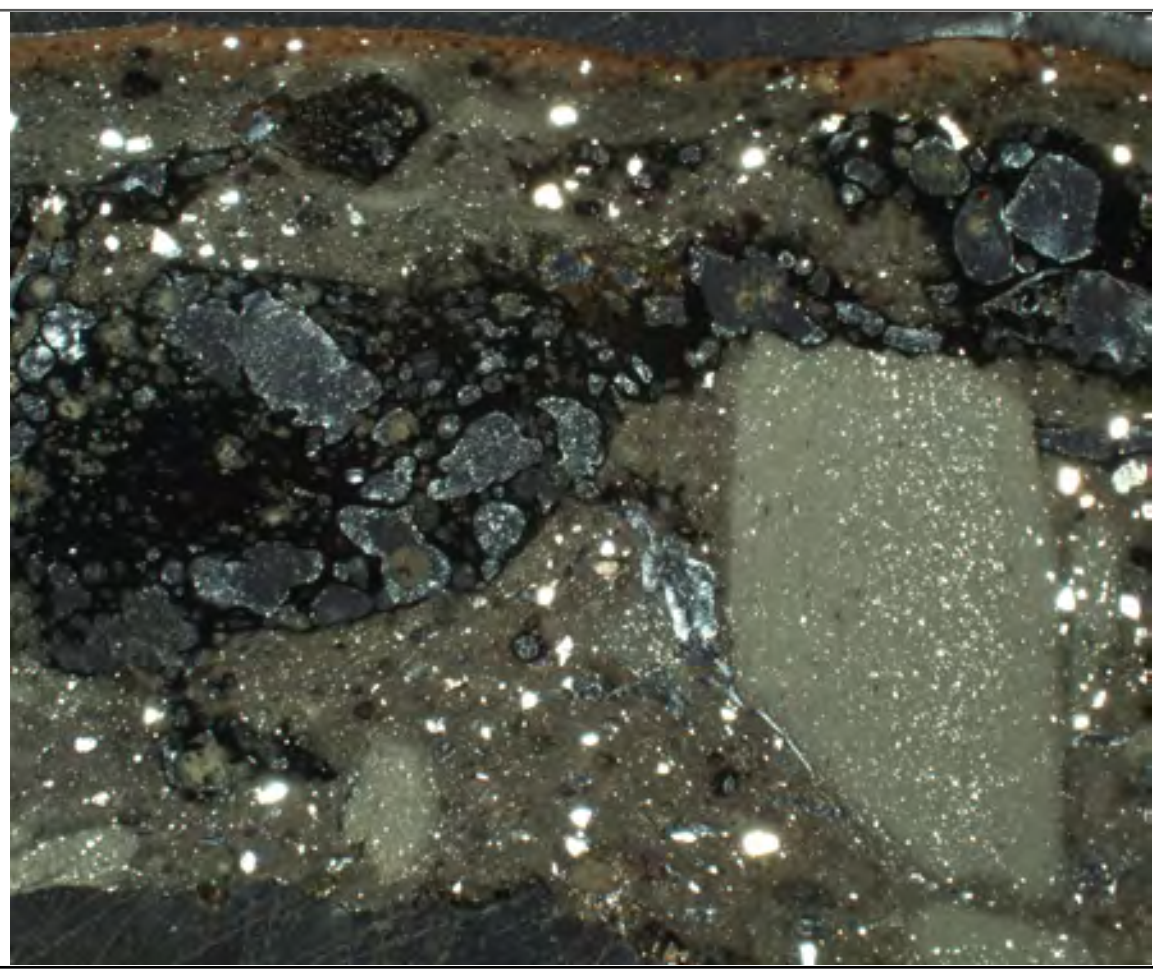</td></tr>
<tr><td>Sample Q40: PPL, x2.5, field of view 6.54mm</td><td>Sample Q40: XP, x2.5, field of view 6.54mm</td></tr>
<tr><td>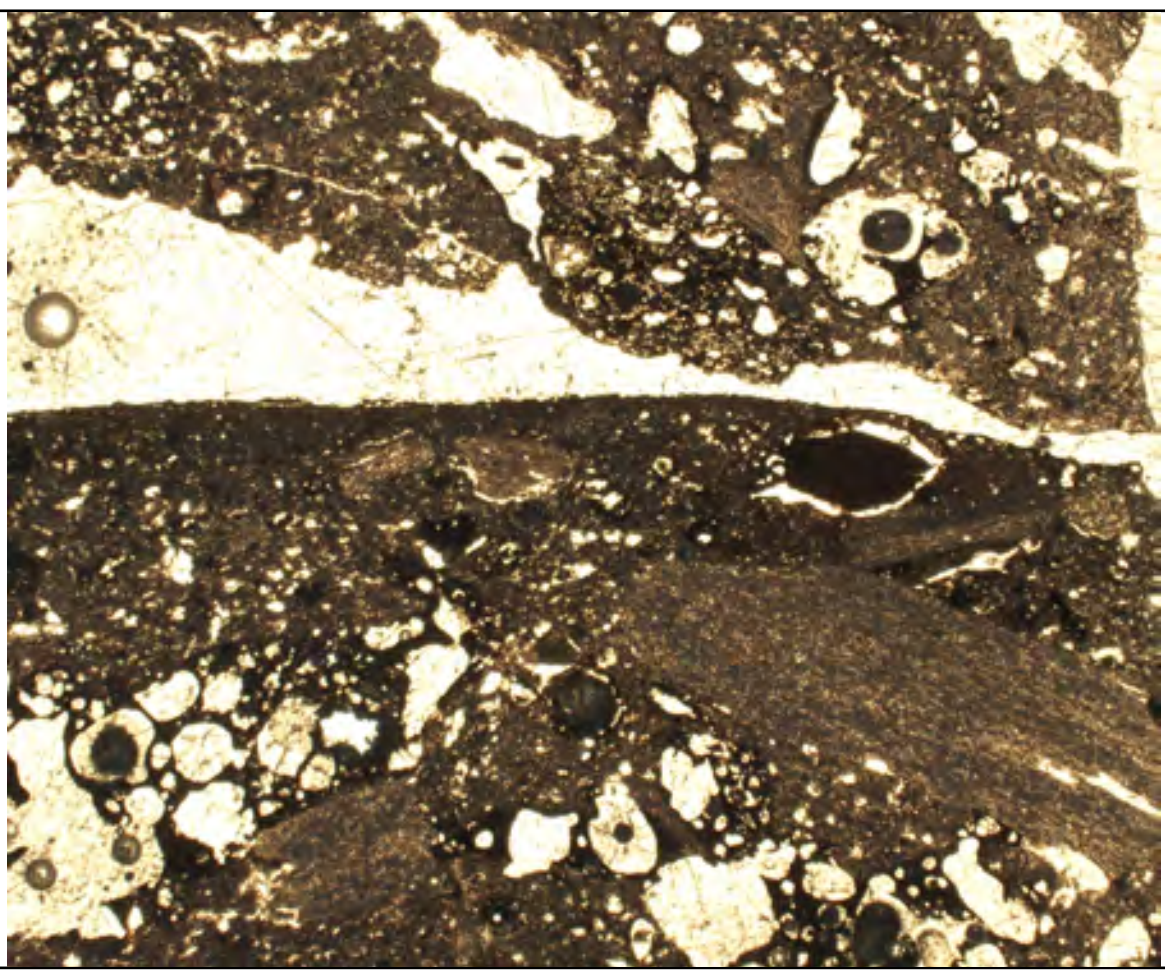</td><td>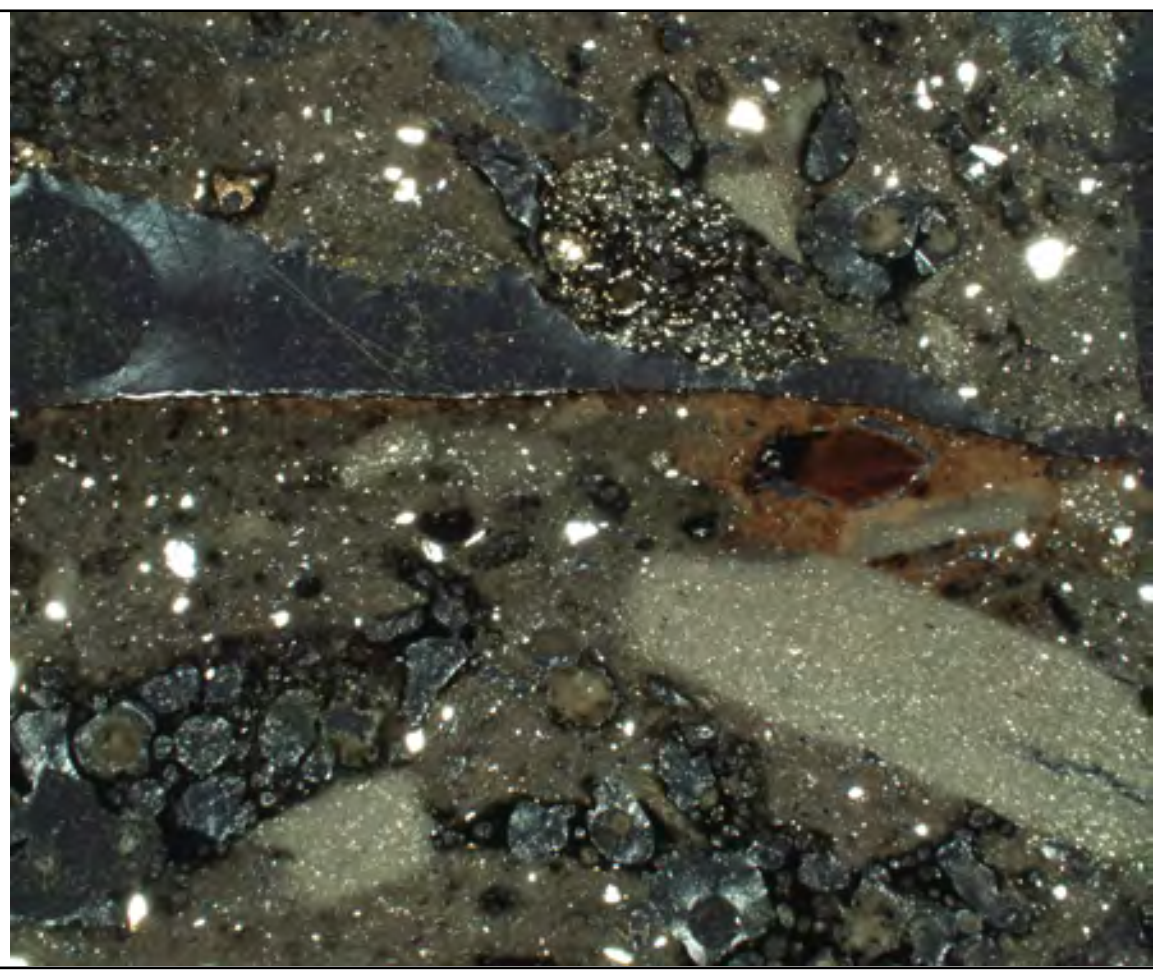</td></tr>
<tr><td>Sample Q40: PPL, x2.5, field of view 6.54mm</td><td>Sample Q40: XP, x2.5, field of view 6.54mm</td></tr>
<tr><td>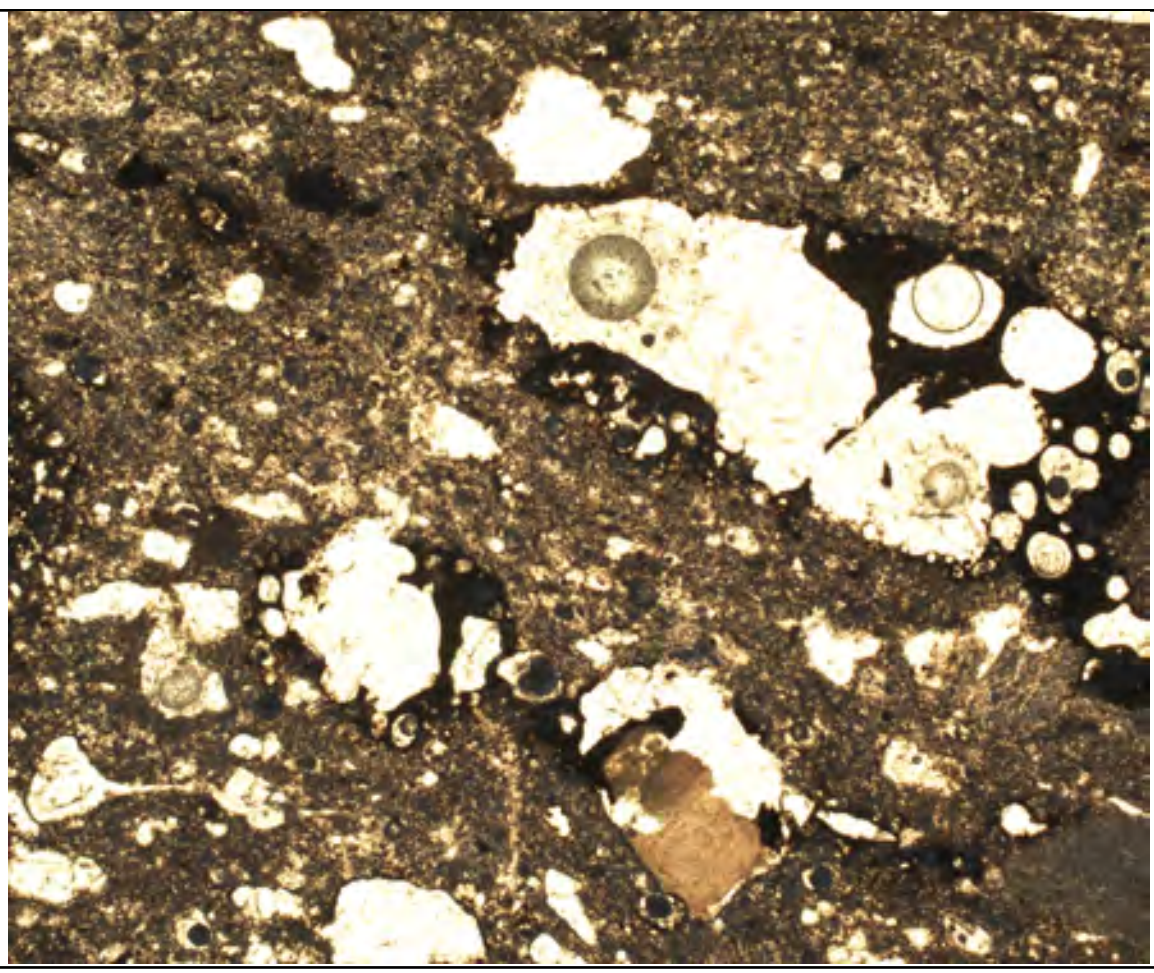</td><td>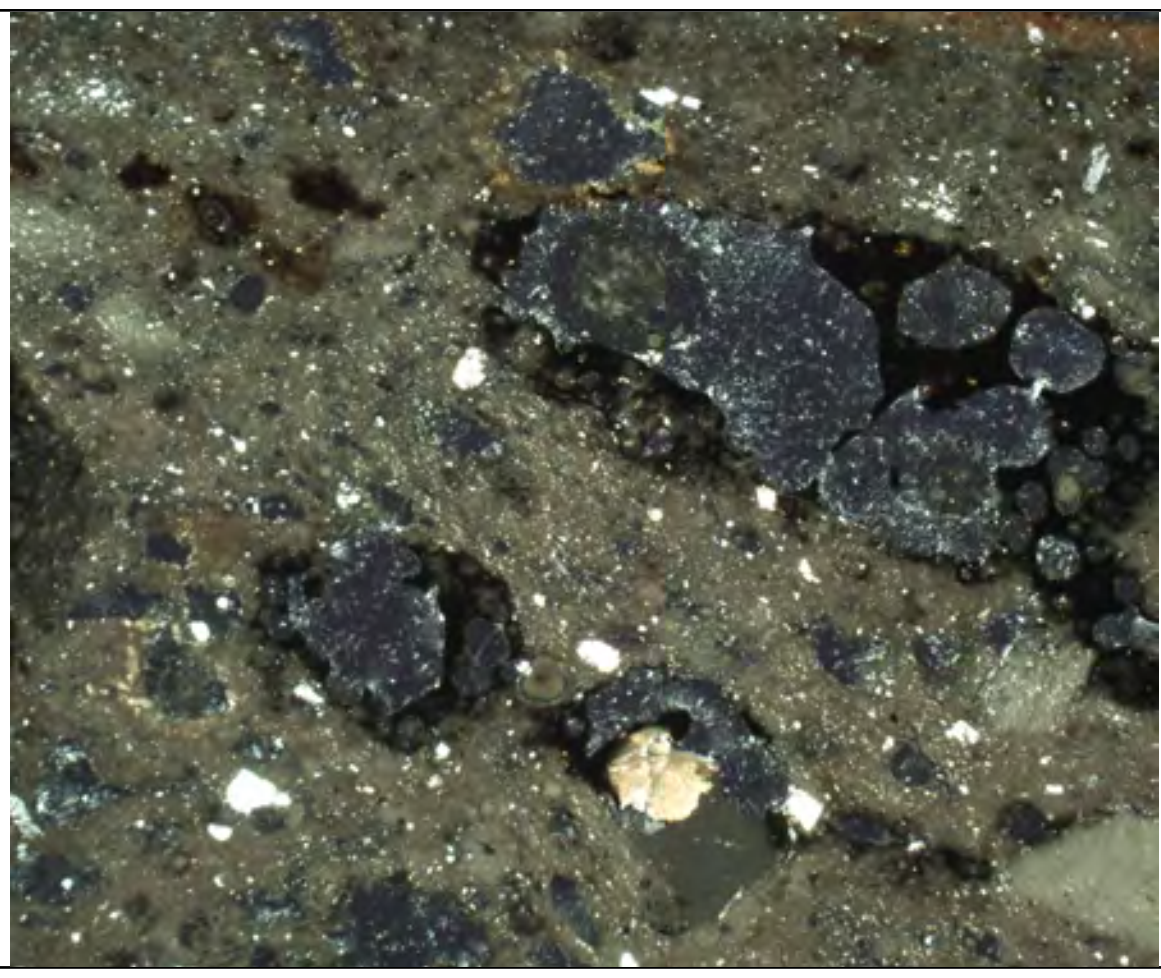</td></tr>
<tr><td>Sample Q40: PPL, x5, field of view 3.39mm</td><td>Sample Q40: XP, x5, field of view 3.39mm</td></tr>
</table>

Inclusions
Inclusions reach maximum frequencies and sizes of respectively 15-20% and 2.8mm, show elongated and angular shapes, are close to open-spaced, randomly oriented, and feature bimodal grain-size distributions.

Coarse Fraction
The coarse fraction represents 50% of the total inclusions and shows sizes comprised and 0.6 and 2.8mm.

Predominant	›	Shales, elongate, angular, 0.6-2.8mm, mode: 1-1.5mm
Very few	›	Siltstones, elongate, subangular, 1.2-2.25mm, mode: 1-2mm
Rare to absent	›	Sandstones, elongate to equant, subangular, mode: 1-1.4mm

Fine Fraction
The fine fraction accounts for 50% with sizes ranging between 0.02 and 0.6mm.

Predominant	›	Quartz
Frequent	›	Shales
	›	Oxides
Few to very rare	›	Mica muscovite
Very few to absent	›	Sandstones

Matrix
The matrix accounts for 70%, is non-calcareous and heterogeneous in color, with large dark grey to black cores comprised between thin reddish-brown margins (XP). The sandwich structure is less evident under PPL. The matrix is optically inactive except for Q27 (grano-striated b fabric), which is also distinguished by a siltier and micaceous matrix.

Voids
The porosity reaches up to 10% and is primarily composed of elongated and randomly oriented meso- to macro vughs.

Qurayyah 1d

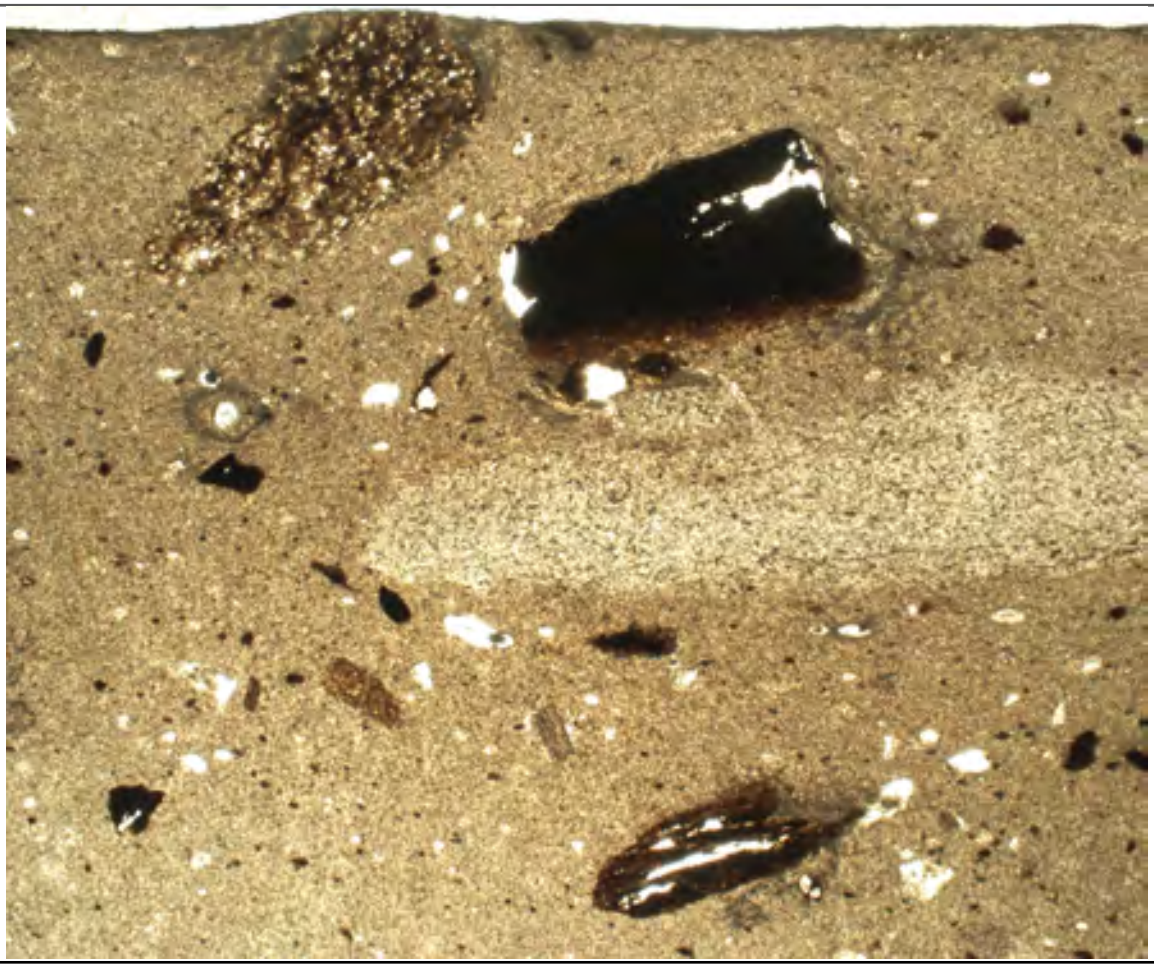	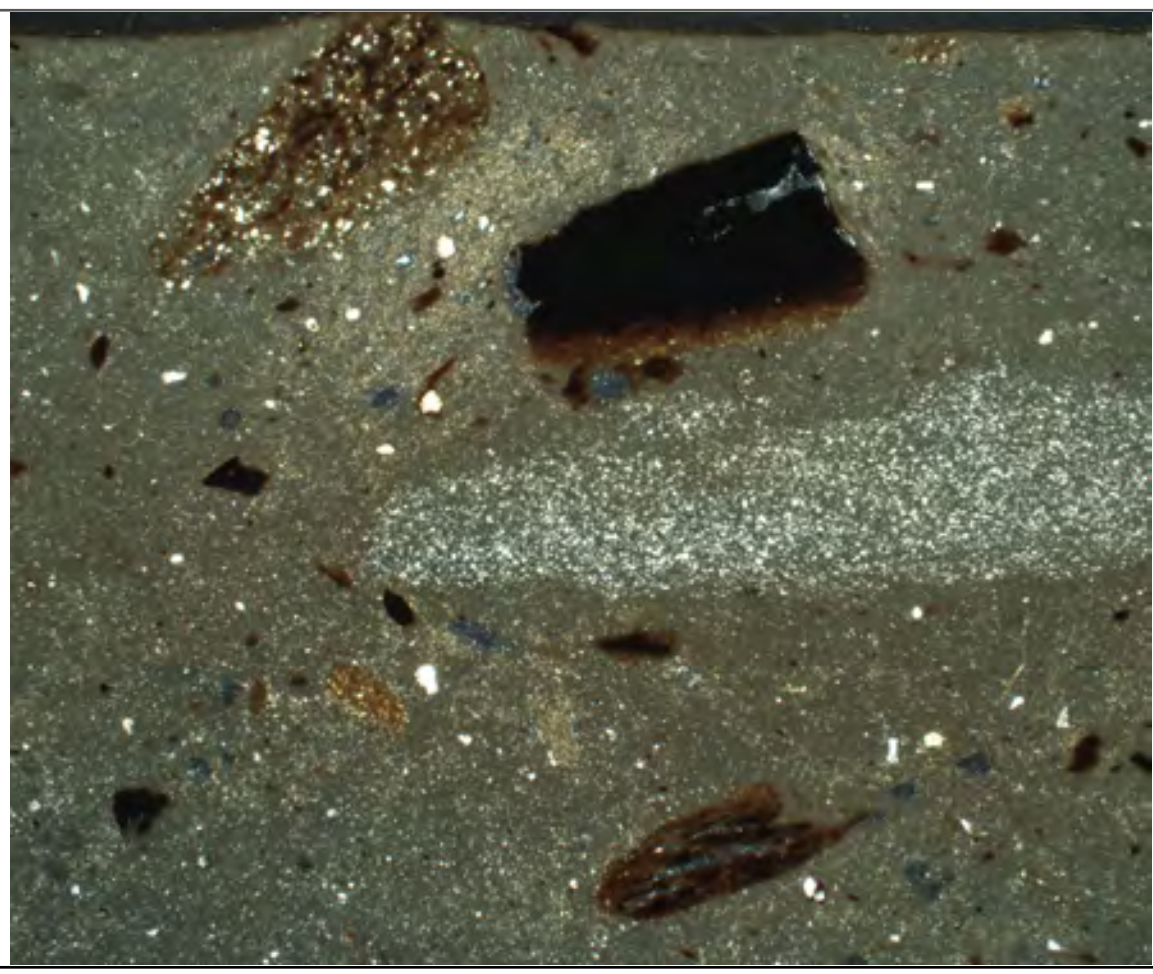
Sample **Q29**: PPL, x2.5, field of view 6.54mm	Sample **Q29**: XP, x2.5, field of view 6.54mm
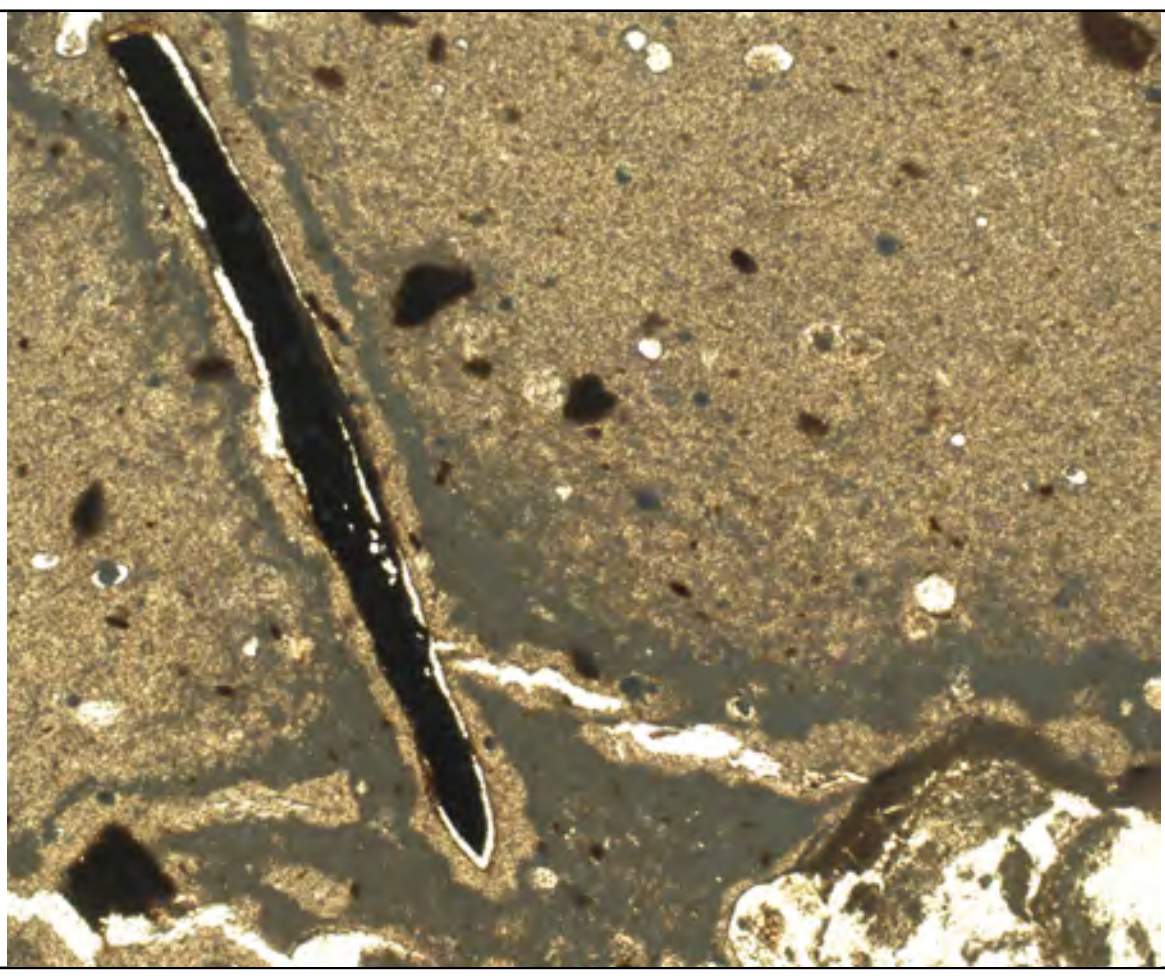	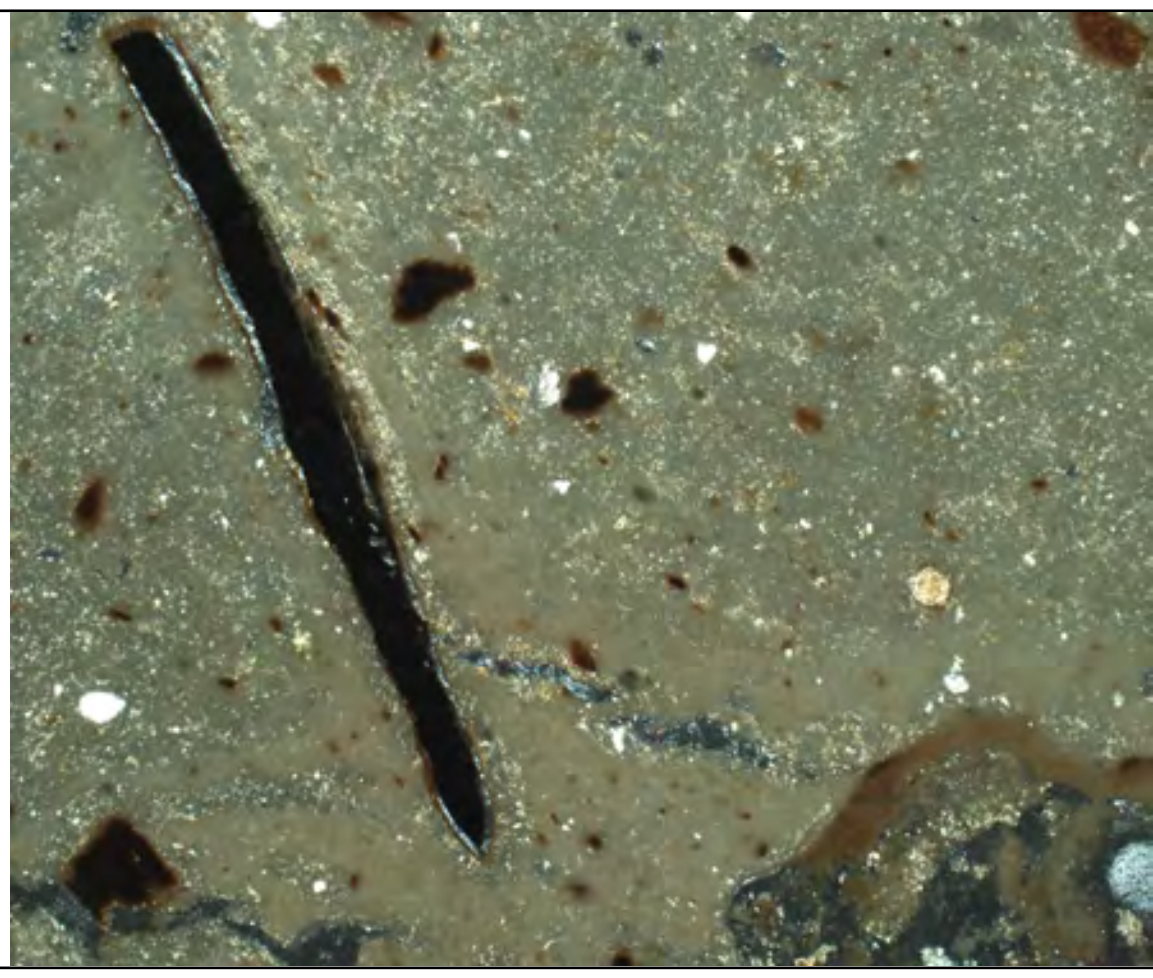
Sample **Q29**: PPL, x5, field of view 3.39mm	Sample **Q29**: XP, x5, field of view 3.39mm
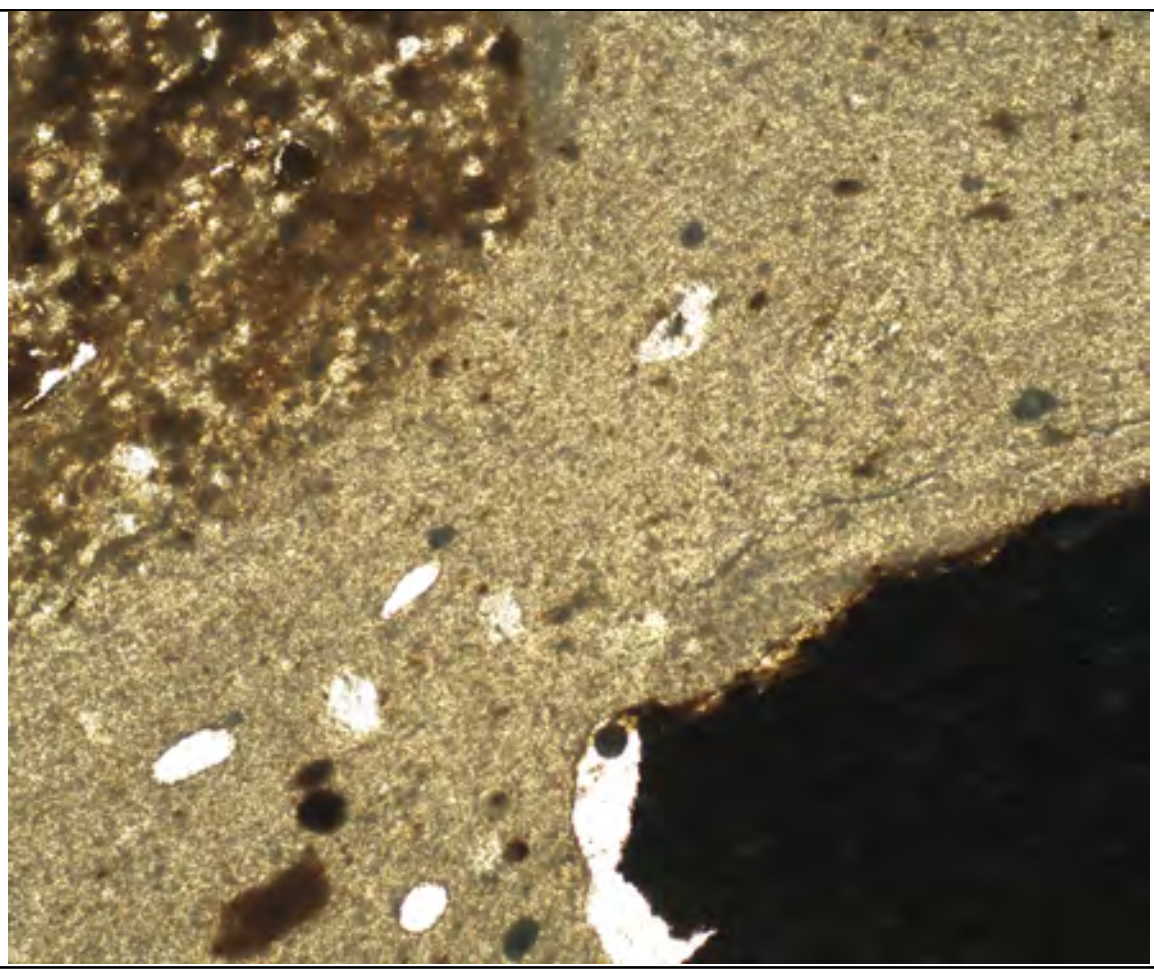	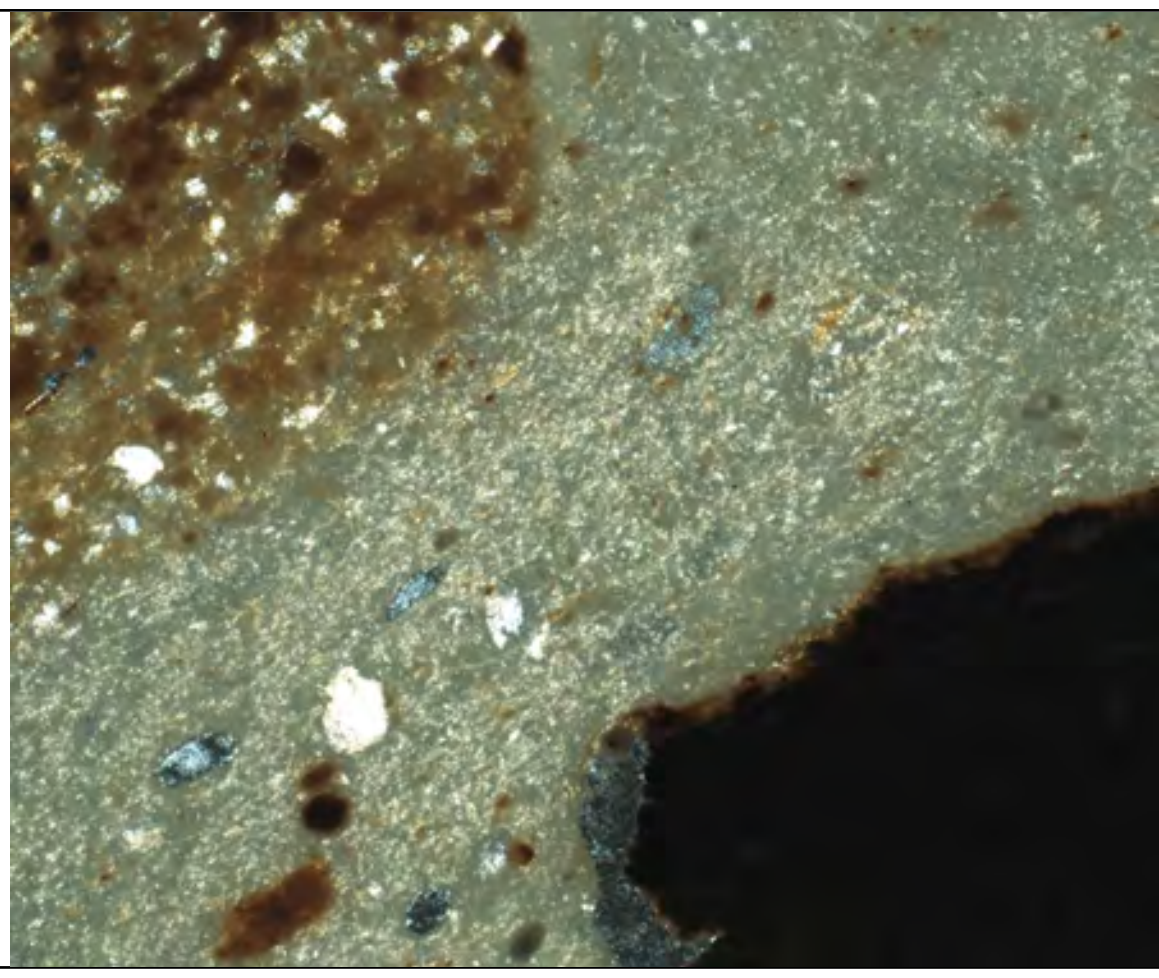
Sample **Q29**: PPL, x10, field of view 1.69mm	Sample **Q29**: XP, x10, field of view 1.69mm

Inclusions
Inclusions reach maximum frequencies and sizes of respectively 7% and 2.9mm, show elongated and angular shapes, are open-spaced, slightly to randomly oriented, and feature bimodal grain-size distributions.

Coarse Fraction
The coarse fraction represents 50% of the total inclusions and shows sizes comprised between 0.9 and 2.9mm.

Predominant	› Shales, elongate, angular, 0.9-2.9mm, mode: 0.9-1mm
Very rare to absent	› Sandstone, elongate, subangular, 1.6mm

Fine Fraction
The fine fraction accounts for 50% with sizes ranging between 0.02 and 0.9mm.

Predominant	› Mica muscovite
Frequent	› Fe-Oxides
Common	› Quartz
Rare	› Shales
Very rare to absent	› Sandstones

Matrix
The matrix accounts for 86%, is micaceous and features beige-yellowish-greenish colors both in PPL and XP. The matrix is optically highly active with grano-striated b-fabrics and exhibits heterogeneities in the form of lenses of varying colors and structures. Claypellets are also common.

Voids
The porosity ranges between 3% (Q29) and 7% (Q29) and is composed of elongated and parallel micro- to megavughs and channels.
