# Supplementary material for: Creating a brand. Genesis, transformation and diffusion of Qurayyah Painted Ware
Source: PLoS One. 2026 Aug 19;21(8):e0351296. doi: 10.1371/journal.pone.0351296 (PMC13489407; doi:10.1371/journal.pone.0351296)
Supplement: Supplementary file 7 [file pone.0351296.s007.pdf]

CREATING A BRAND.
GENESIS, TRANSFORMATION AND DIFFUSION OF QURAYYAH PAINTED WARE

Luciani M. – Fragnoli P. – Gilstrap W.D. – Sterba J.H. – Klainscek D.– Greene J.A. – Bourke S. – Donnelly P.

Corresponding author: Marta Luciani, University of Vienna, marta.luciani@univie.ac.at

…….

SUPPORTING INFORMATION 7

Detailed descriptions and micrographs of petrogroup Qurayyah 2

All drawings and photos: rights holder Marta Luciani

Petrogroups Description - QURAYYAH

Qurayyah 2a

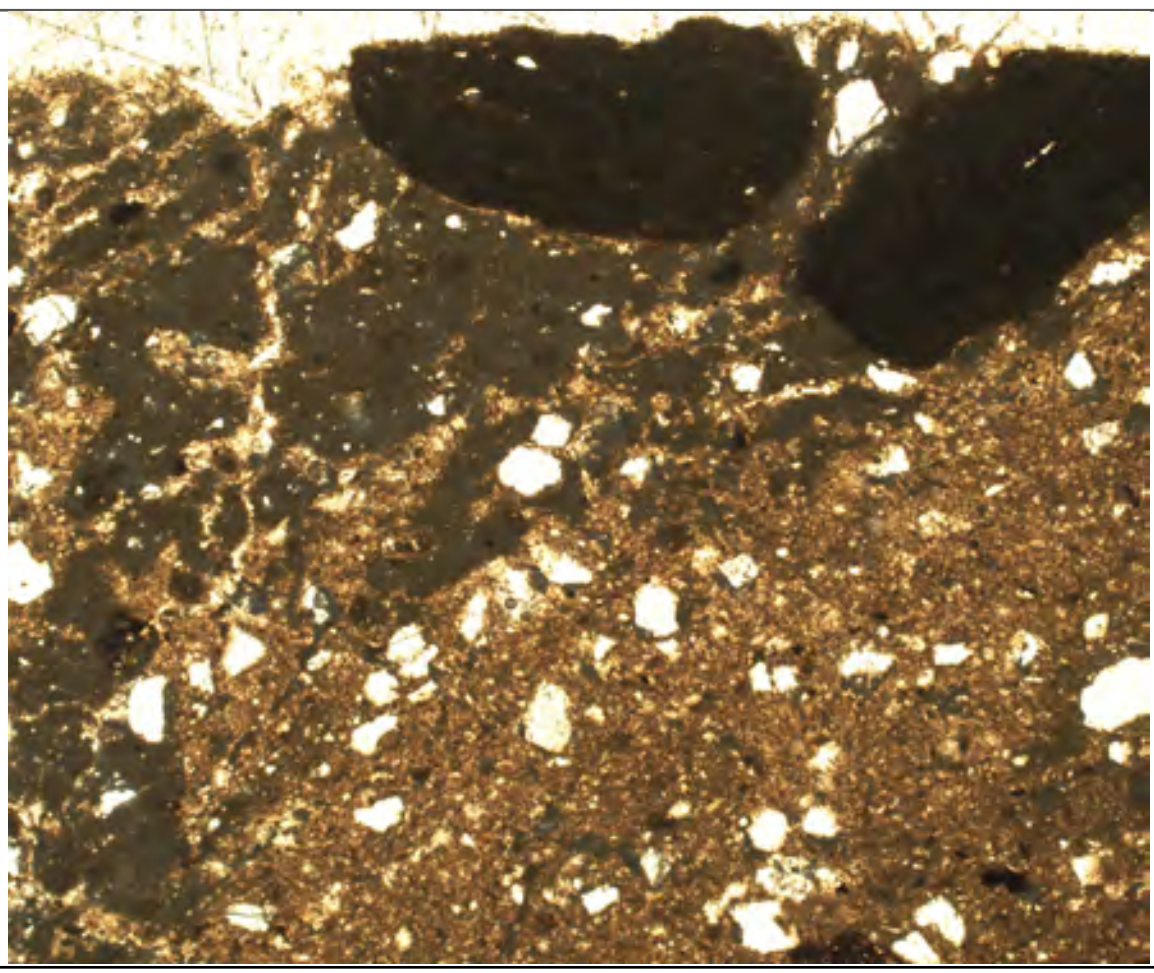	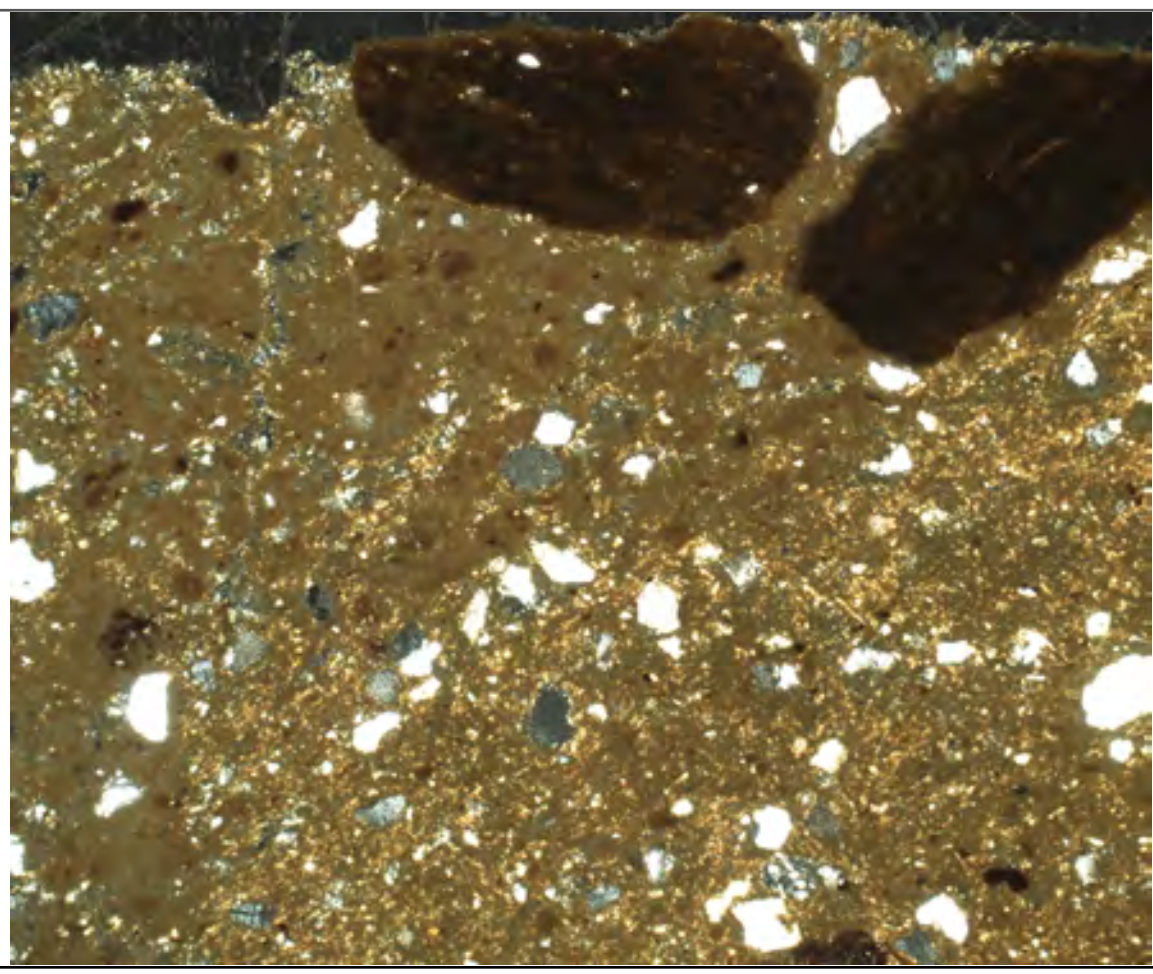
Sample **Q36**: PPL, x5, field of view 3.39mm	Sample **Q36**: XP, x5, field of view 3.39mm
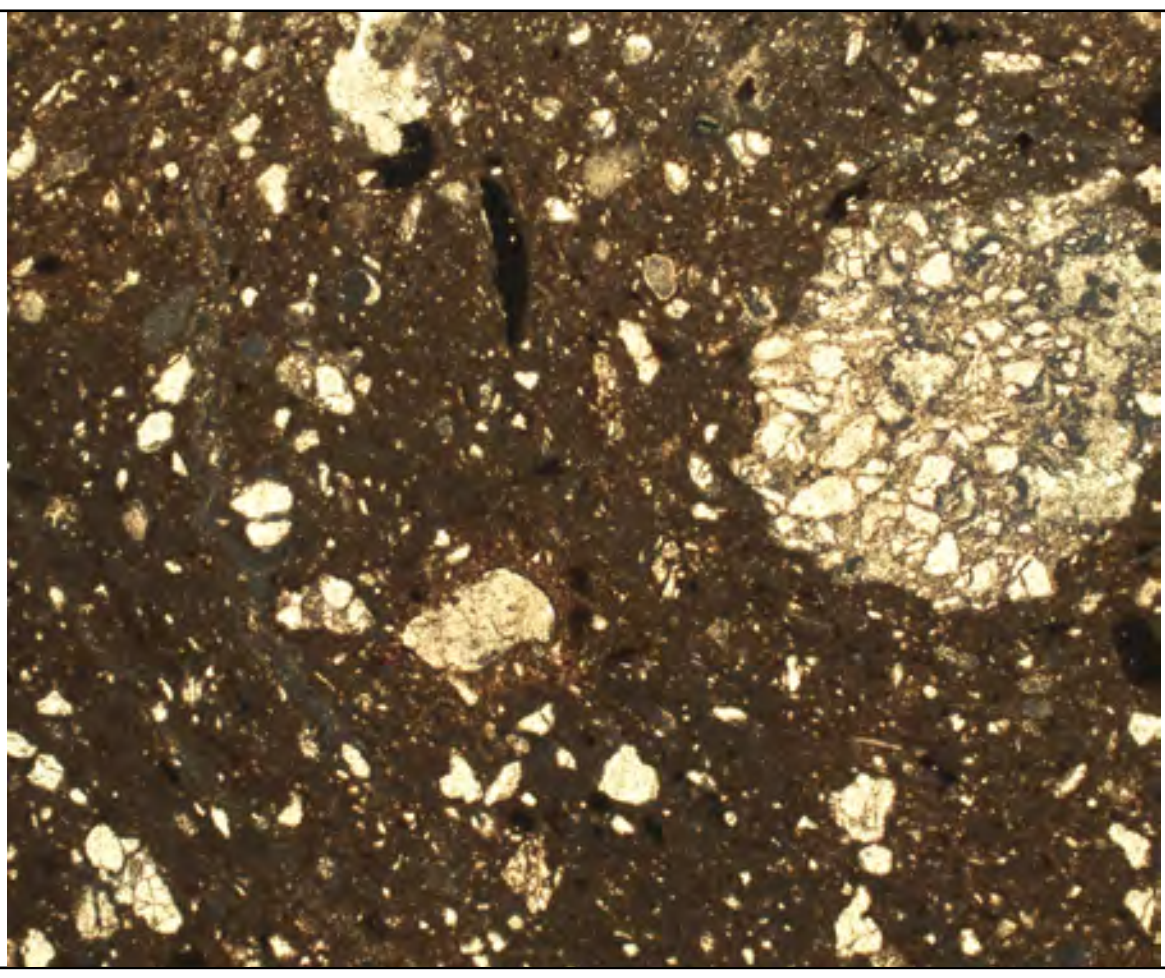	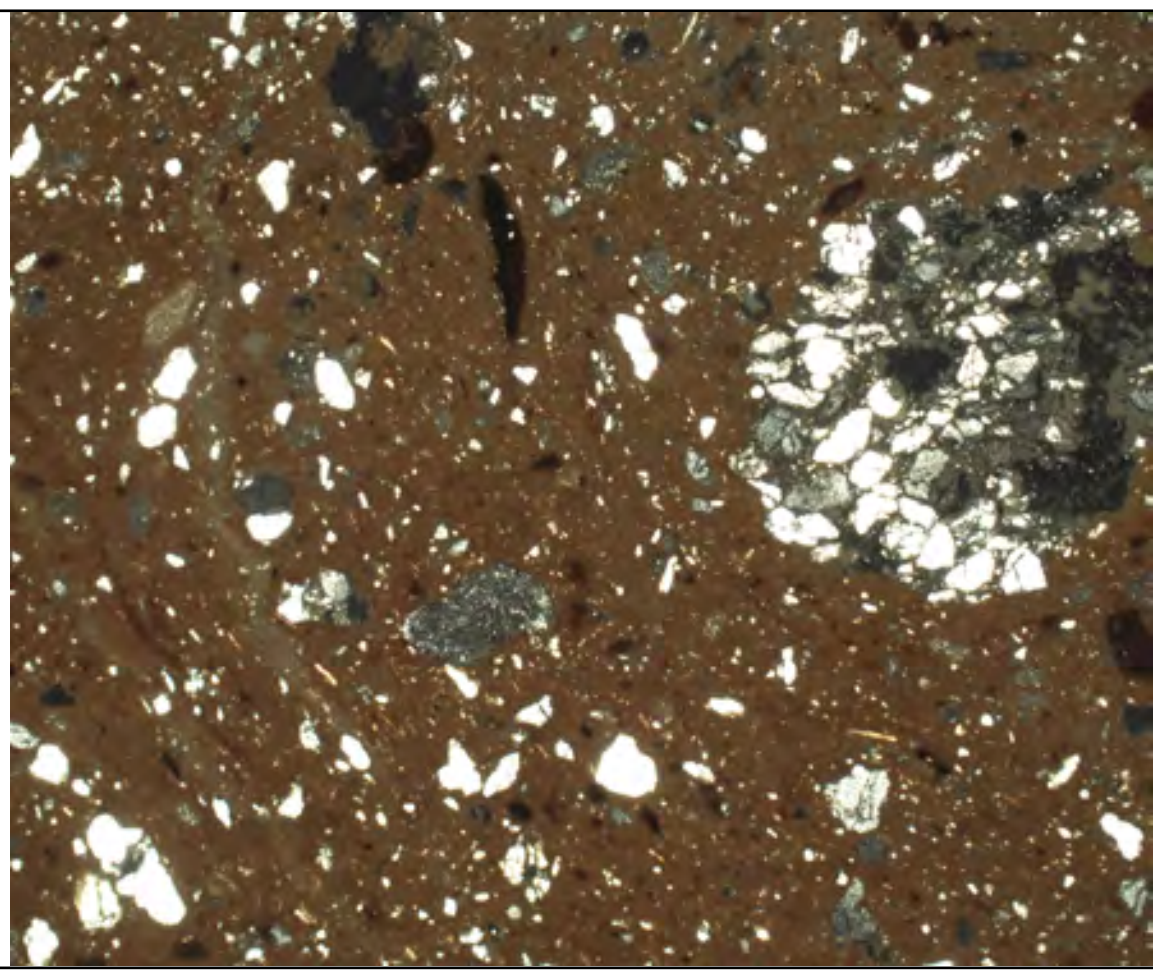
Sample **Q15**: PPL, x5, field of view 3.39mm	Sample **Q15**: XP, x5, field of view 3.39mm
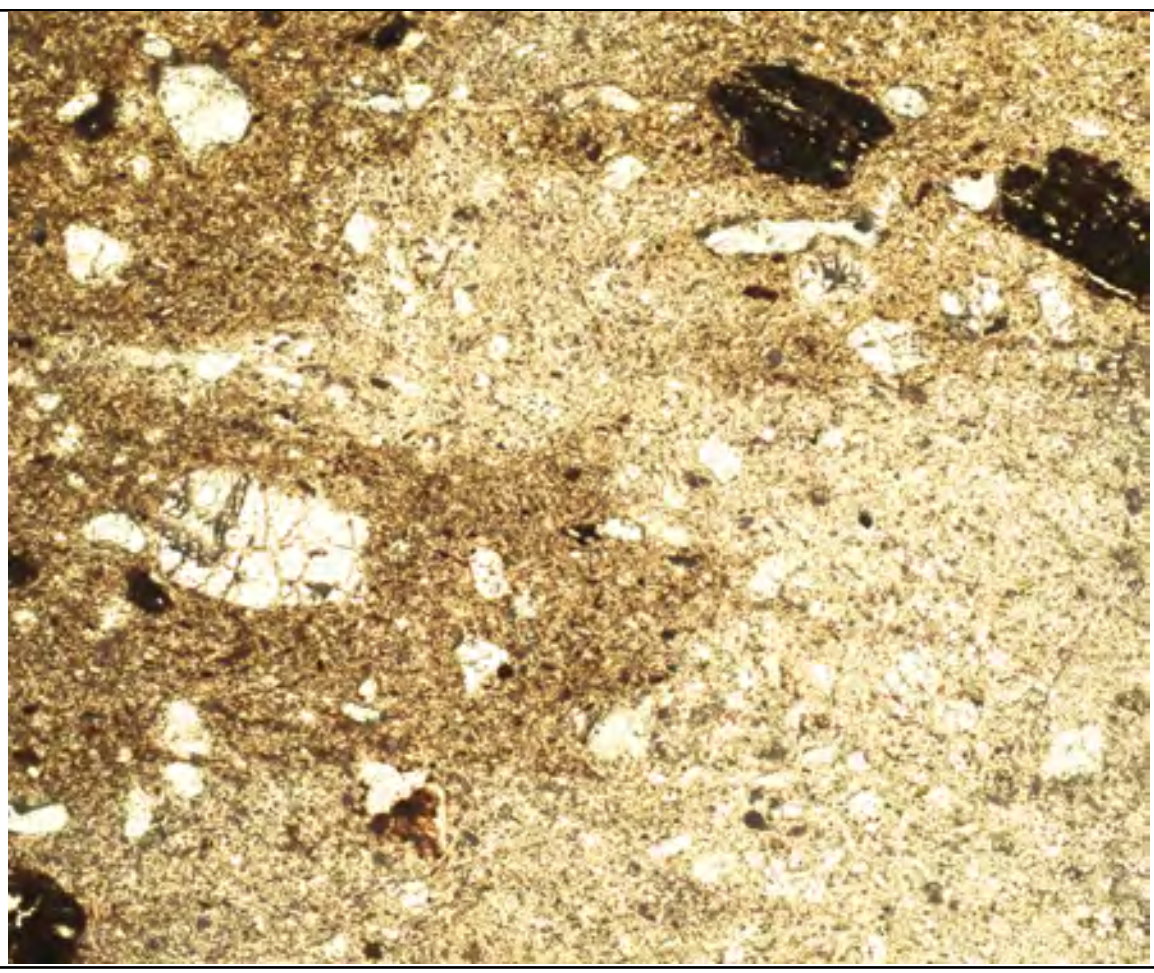	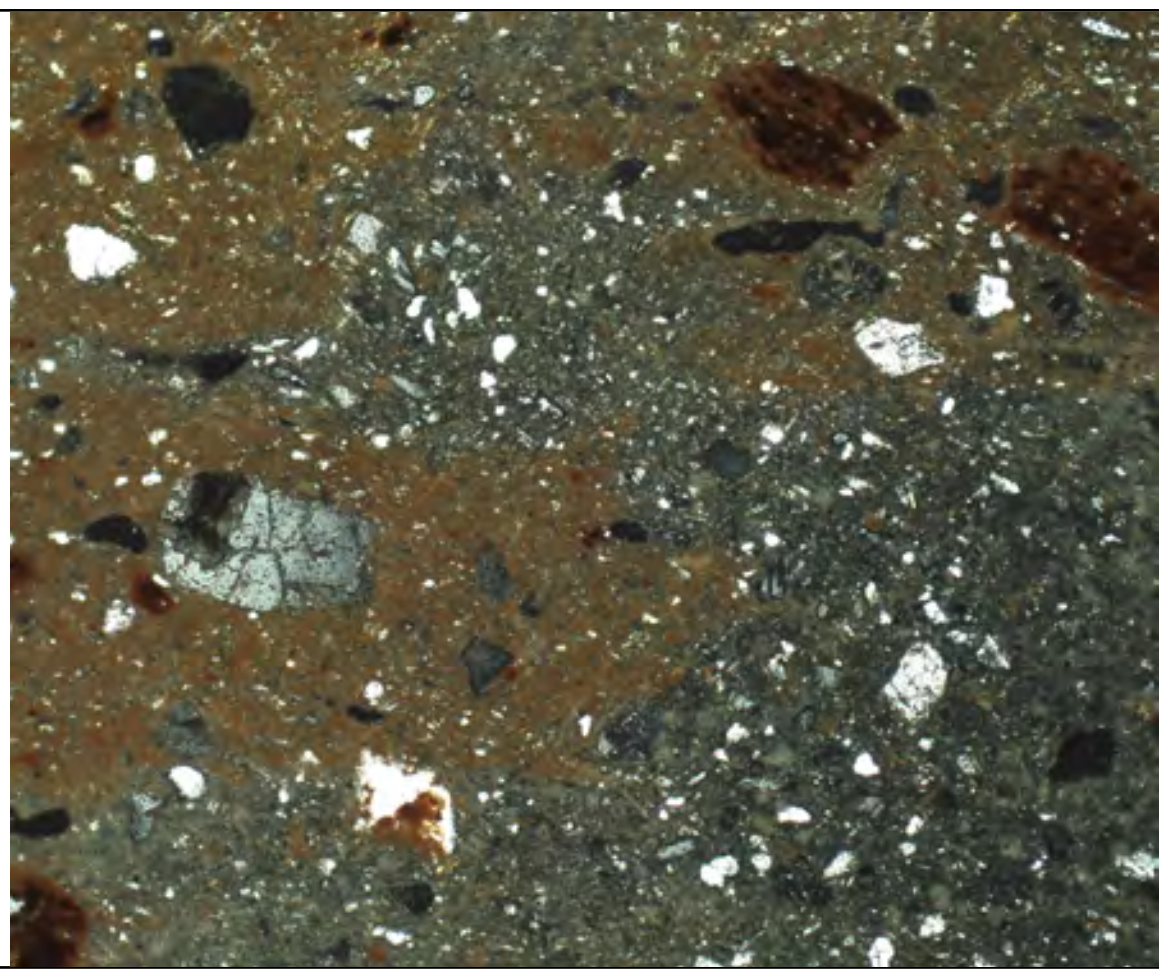
Sample **Q8**: PPL, x10, field of view 1.69mm	Sample **Q8**: XP, x10, field of view 1.69mm

Inclusions
Inclusions reach maximum frequencies and sizes of respectively 10-15% and 2.9mm, show equant and subangular to subrounded shapes, are single- to open-spaced, randomly oriented (except for Q8), and feature uni- to bimodal grain-size distributions.

Coarse Fraction
The coarse fraction represents 20% of the total inclusions and shows sizes comprised between 1.2 and 2.9mm.

Predominant	›	Sandstones, elongate, subangular, 1.2-2.9mm, mode: 1.5-2mm
Rare to absent	›	Shales, elongate, angular to subangular, mode: 1.2-2mm
	›	Siltstones, elongate, angular to subangular, mode: 1.2-2mm

Fine Fraction
The fine fraction accounts for 80% with sizes ranging between 0.02 and 0.9mm.

Predominant	›	Quartz
Dominant to frequent	›	Mica muscovite
Few to common	›	Fe-Oxides
Few	›	Sandstones
Rare to absent	›	Mica biotite
	›	K-feldspar

Matrix
The matrix accounts for 70-83%, is silty and features lenses of varying colors, grain-size distributions and optical activities. Colors in PPL include brown, yellowish-brown, light to dark reddish-brown, dark brown, light grey, beige and orange-brown. Q8 is distinguished by a bichrome distribution of colors, reddish-yellowish brown on the inner side and dark grey on the outer side (in PPL).

Voids
The porosity ranges between 7 and 15% and is composed of randomly oriented (except for Q8) meso- to megavughs and planar voids.

Petrogroups Description - QURAYYAH

Qurayyah 2b

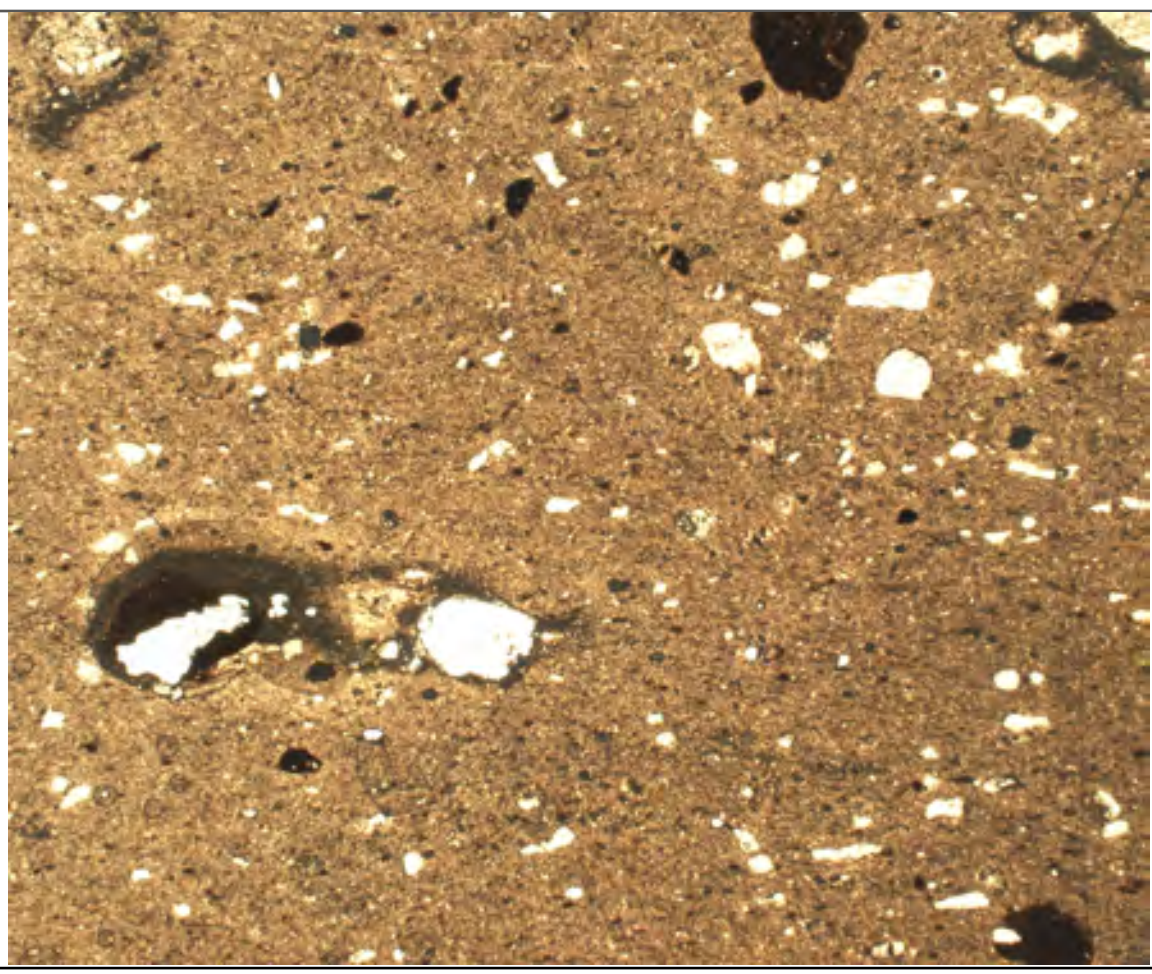

Sample **Q11**: PPL, x2.5, field of view 6.54mm

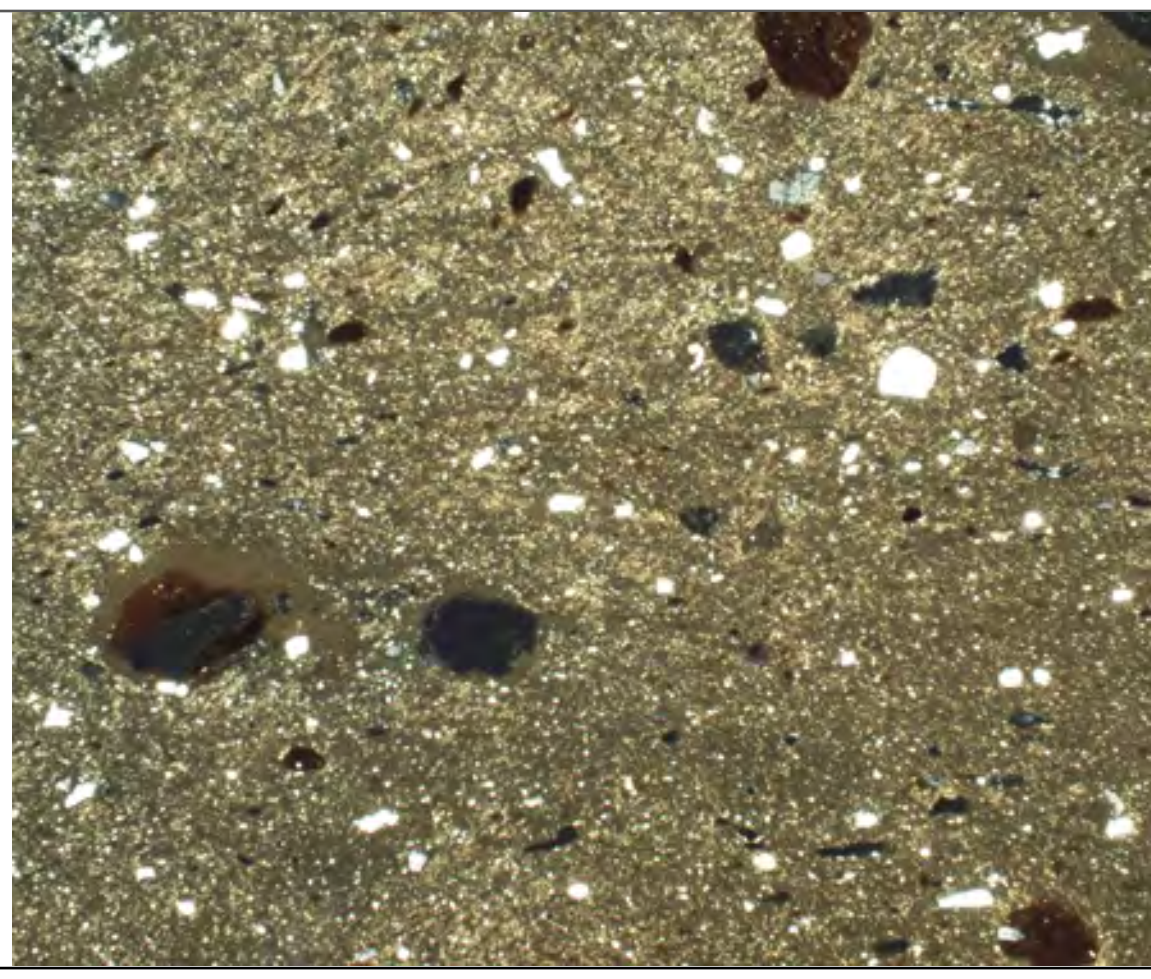

Sample **Q11**: XP, x2.5, field of view 6.54mm

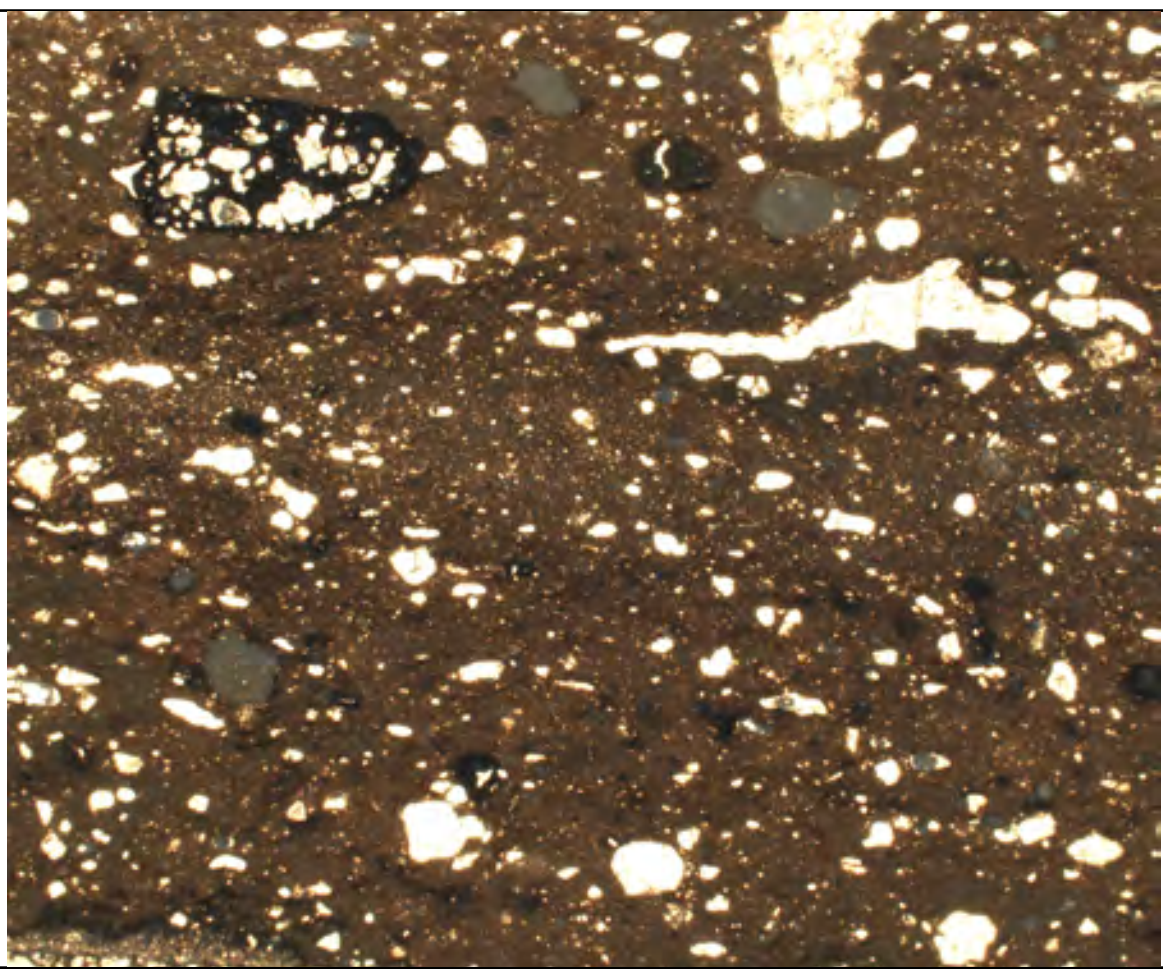

Sample **Q9**: PPL,x2.5, field of view 6.54mm

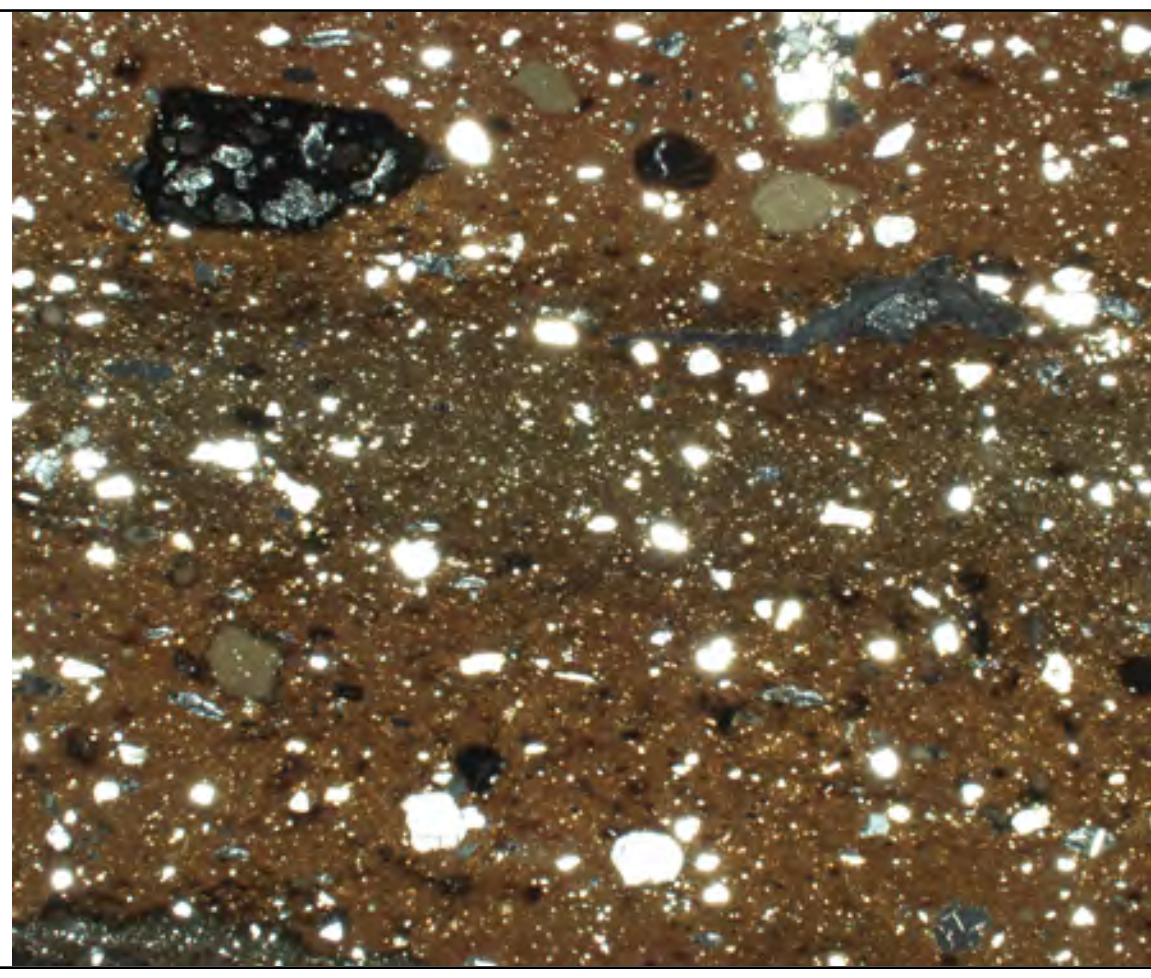

Sample **Q9**: XP, x2.5, field of view 6.54mm

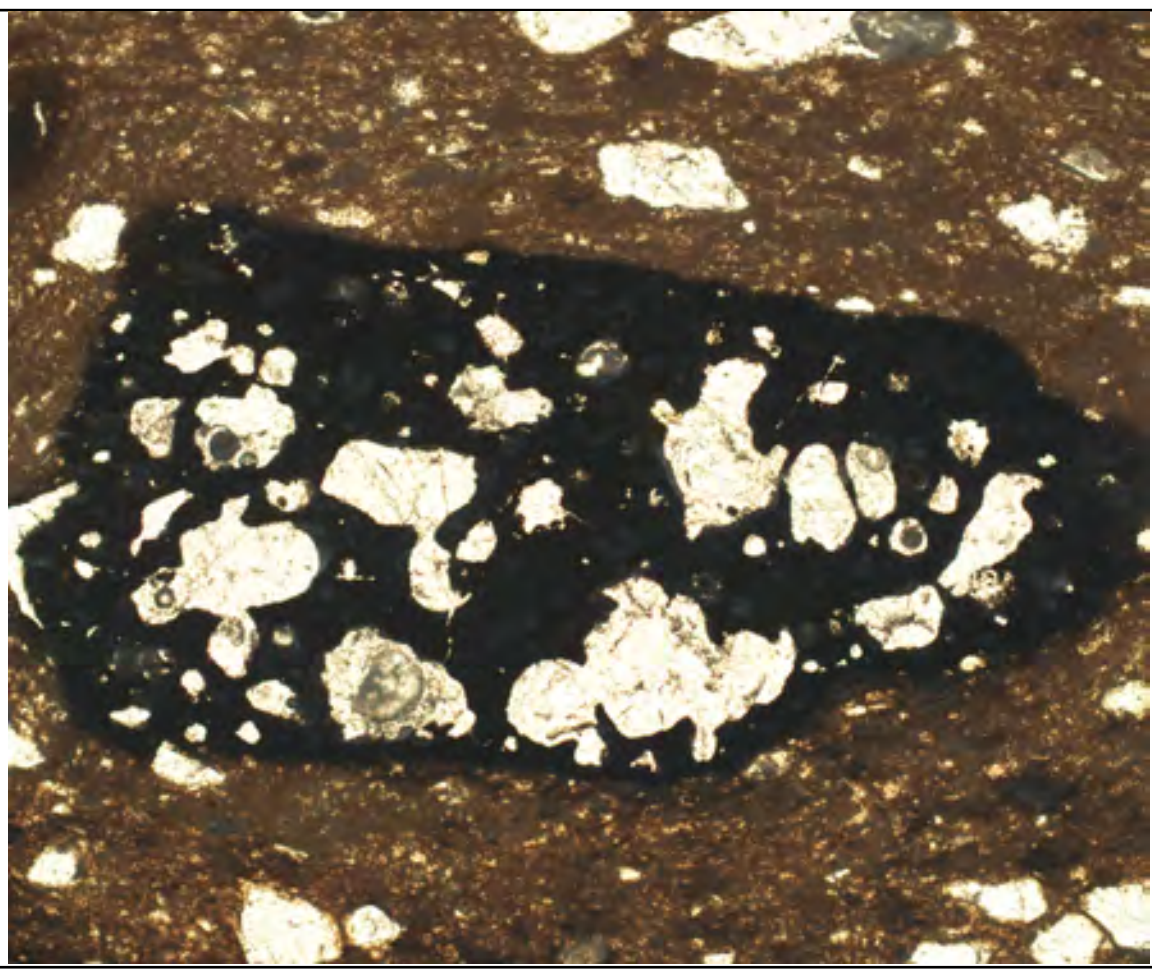

Sample **Q9**: PPL, x10, field of view 1.69mm

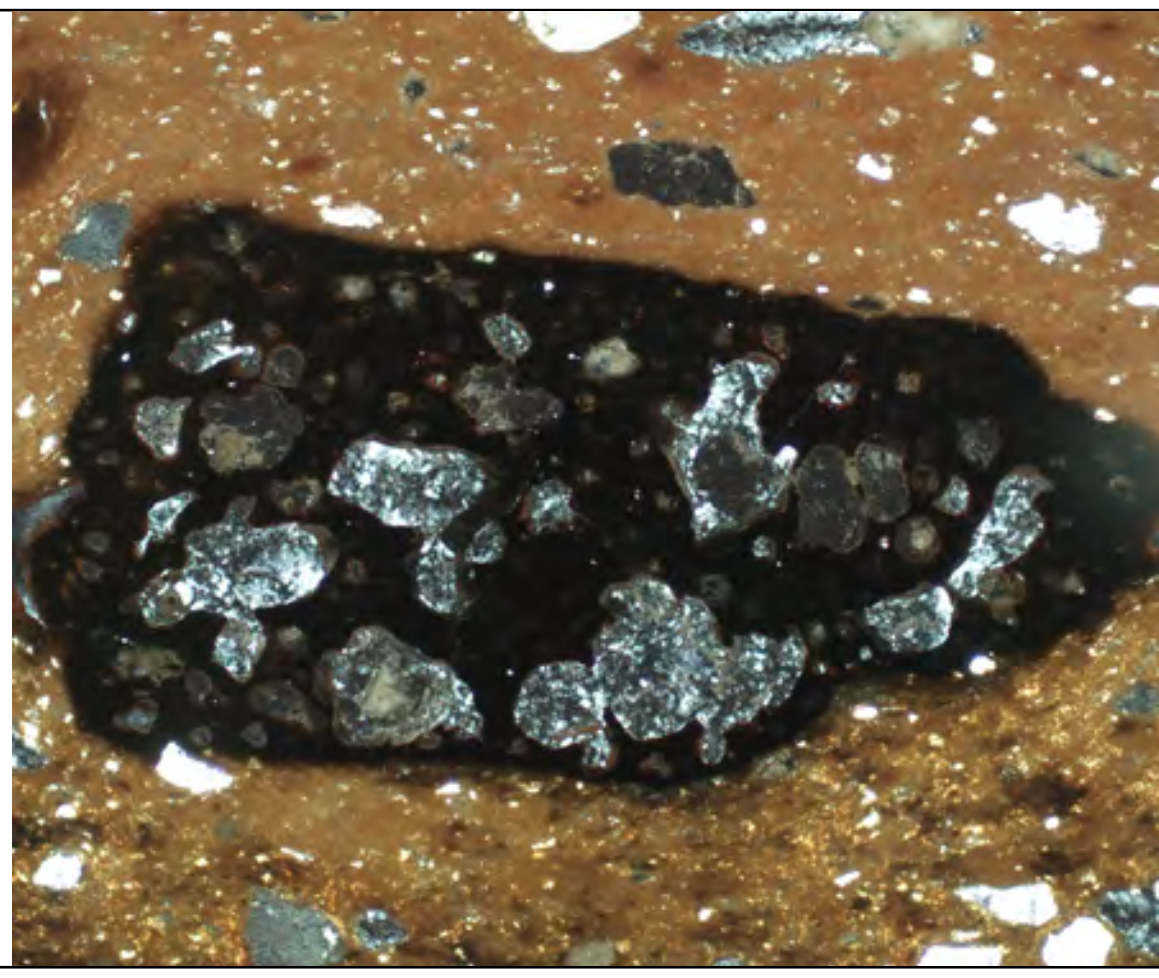

Sample **Q9**: XP, x10, field of view 1.69mm

Inclusions
Inclusions reach maximum frequencies and sizes of respectively 7-15% and 1.85mm, show elongate to equant and subangular to subrounded shapes, are closed- to open-spaced, randomly oriented (except for Q9 and Q10), and feature uni- to bimodal grain-size distributions.

Coarse Fraction
Same as Qurayyah 2a, but shales (also with degassing pores) prevail over sandstones.

Fine Fraction
Same as Qurayyah 2a.

Matrix
Same as Qurayyah 2a. Q9 shows a sandwich structure consisting of a brown-greenish core and red-brownish margins. Q31 has grey-greenish inner side and light brown-reddish outer side.

Voids
Same as Qurayyah 2a. Q9 and Q10 features an oriented porosity.
