# Supplementary material for: Creating a brand. Genesis, transformation and diffusion of Qurayyah Painted Ware
Source: PLoS One. 2026 Aug 19;21(8):e0351296. doi: 10.1371/journal.pone.0351296 (PMC13489407; doi:10.1371/journal.pone.0351296)
Supplement: Supplementary file 8 [file pone.0351296.s008.pdf]

CREATING A BRAND.
GENESIS, TRANSFORMATION AND DIFFUSION OF QURAYYAH PAINTED WARE

Luciani M. – Fragnoli P. – Gilstrap W.D. – Sterba J.H. – Klainscek D.– Greene J.A. – Bourke S. – Donnelly P.

Corresponding author: Marta Luciani, University of Vienna, marta.luciani@univie.ac.at

…….

SUPPORTING INFORMATION 8

Detailed descriptions and micrographs of petrogroup Qurayyah 3 and loners from Qurayyah

All drawings and photos: rights holder Marta Luciani

Petrogroups Description - QURAYYAH

Qurayyah 3

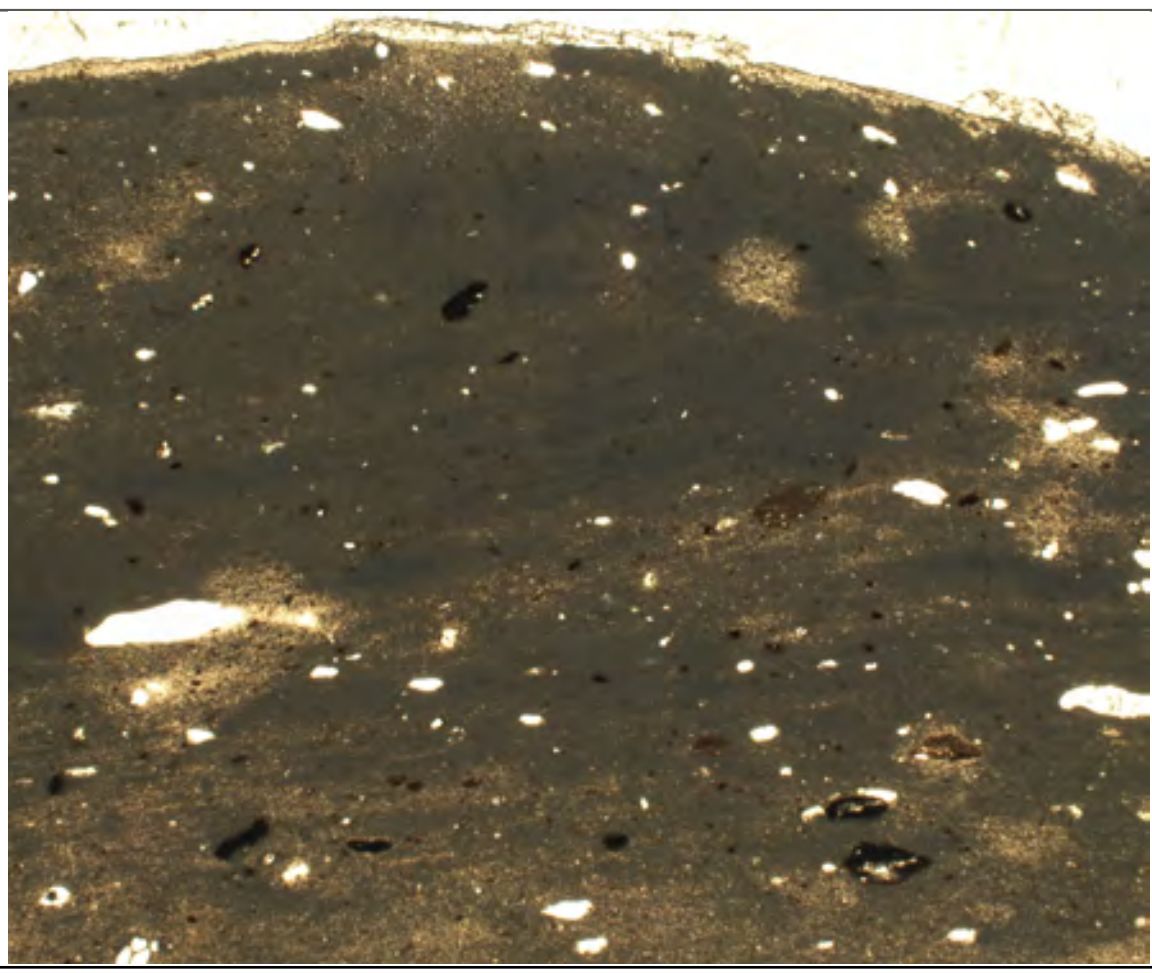

Sample **Q3**: PPL, x2.5, field of view 6.54mm

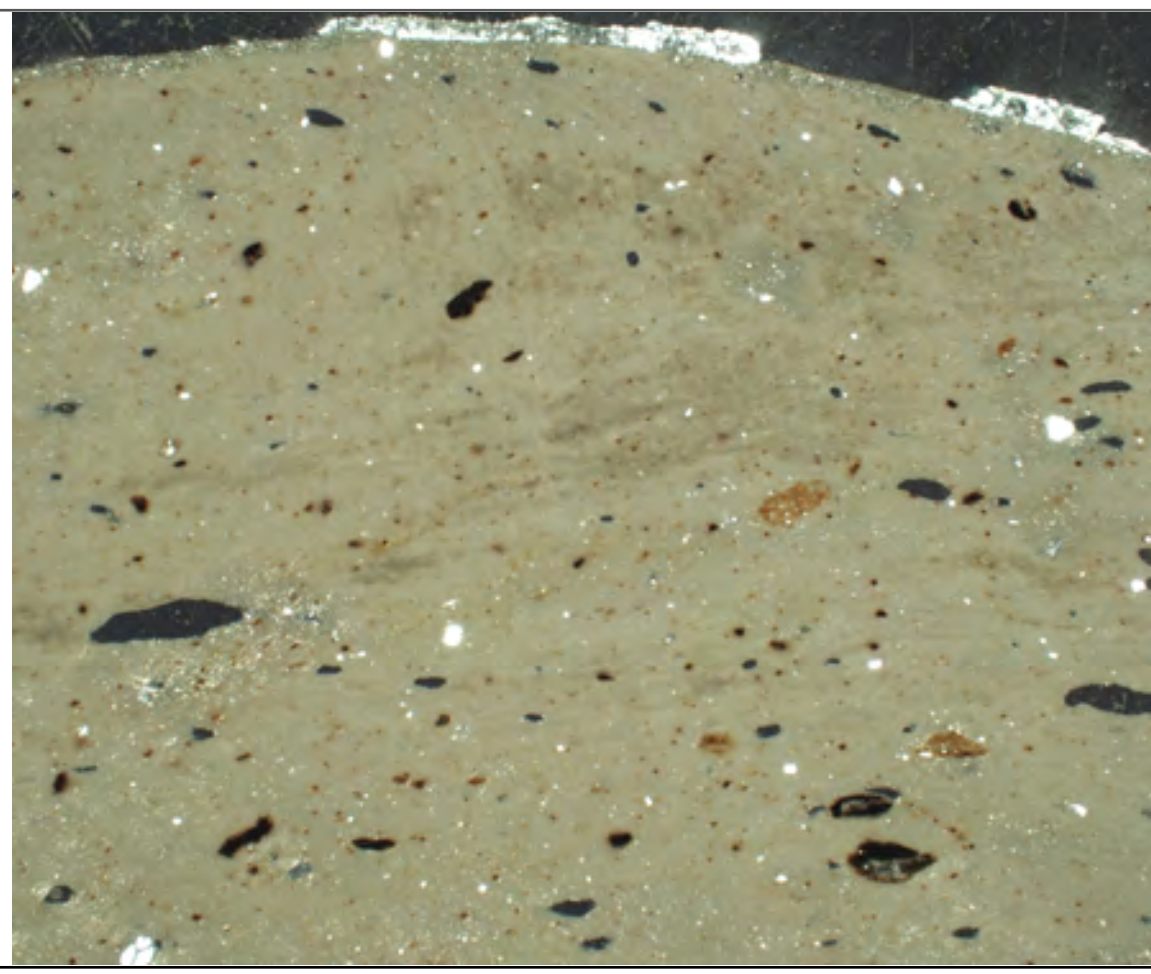

Sample **Q3**: XP, x2.5, field of view 6.54mm

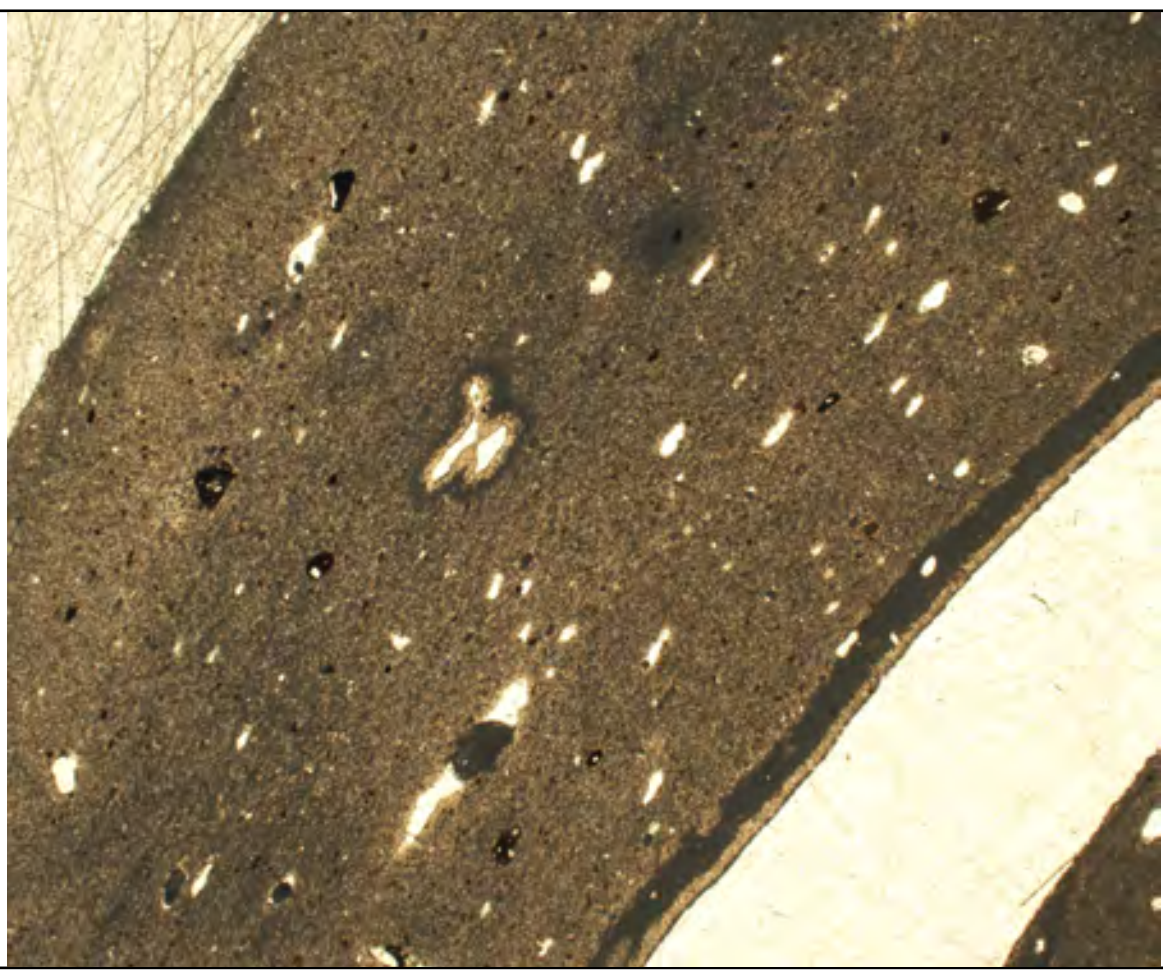

Sample **Q3**: PPL,x2.5, field of view 6.54mm

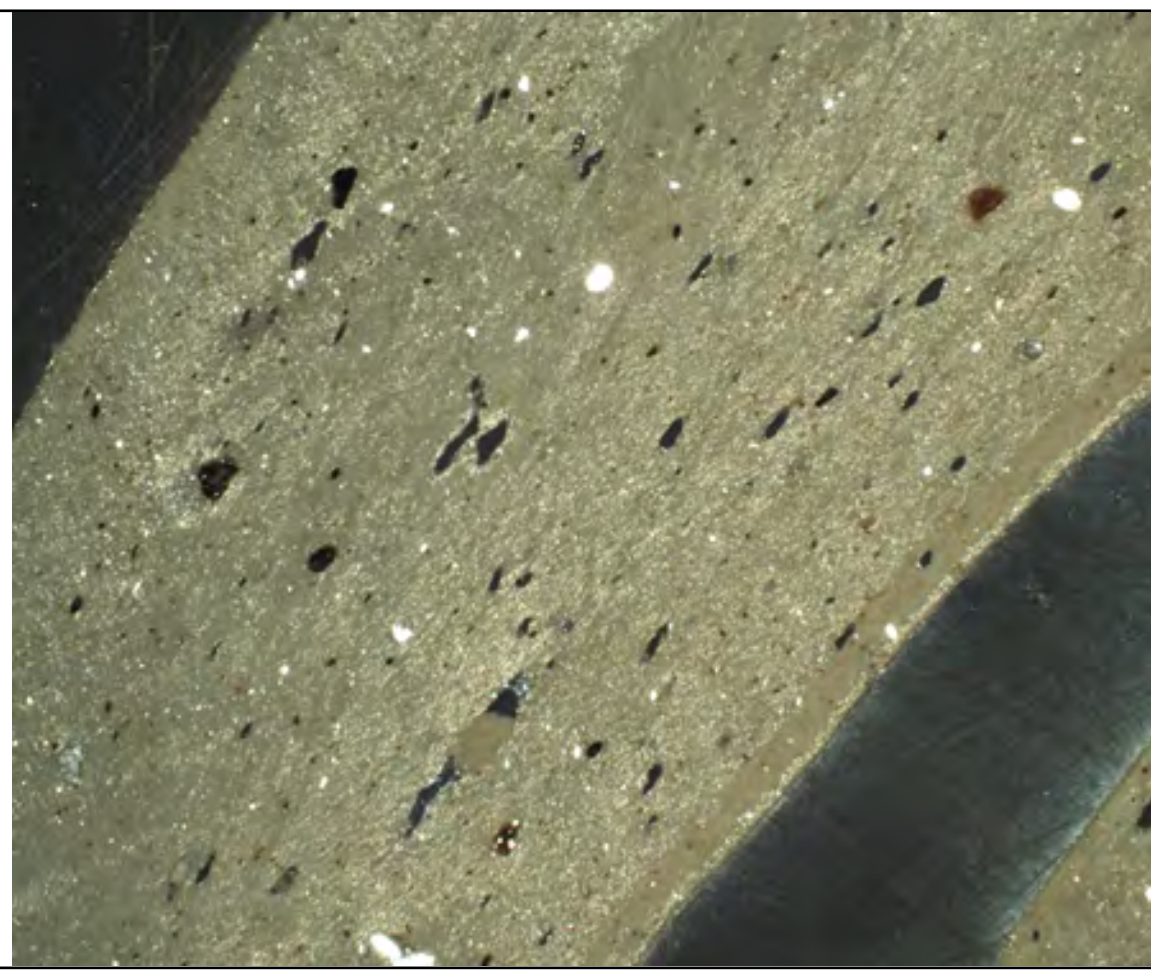

Sample **Q3**: XP, x2.5, field of view 6.54mm

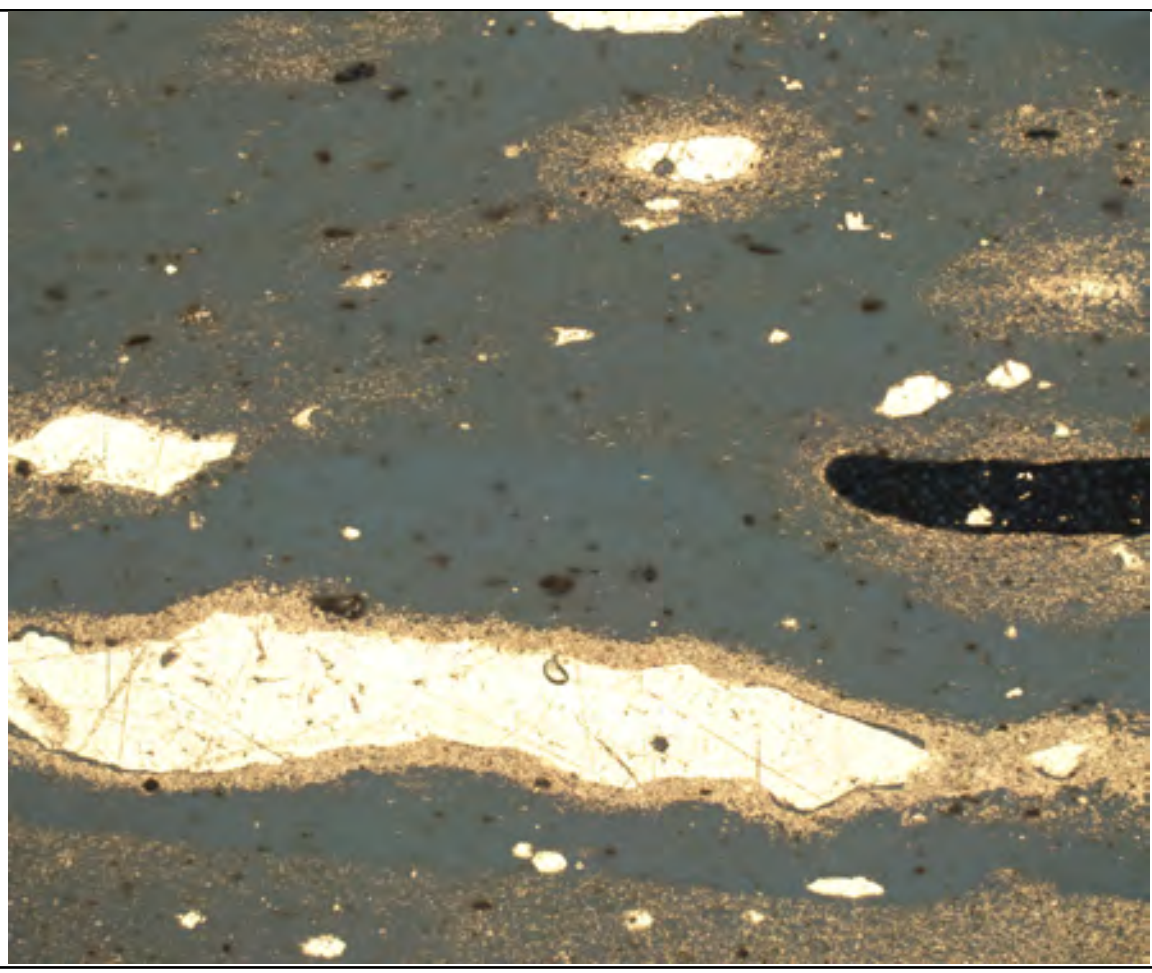

Sample **Q3**: PPL, x10, field of view 1.69mm

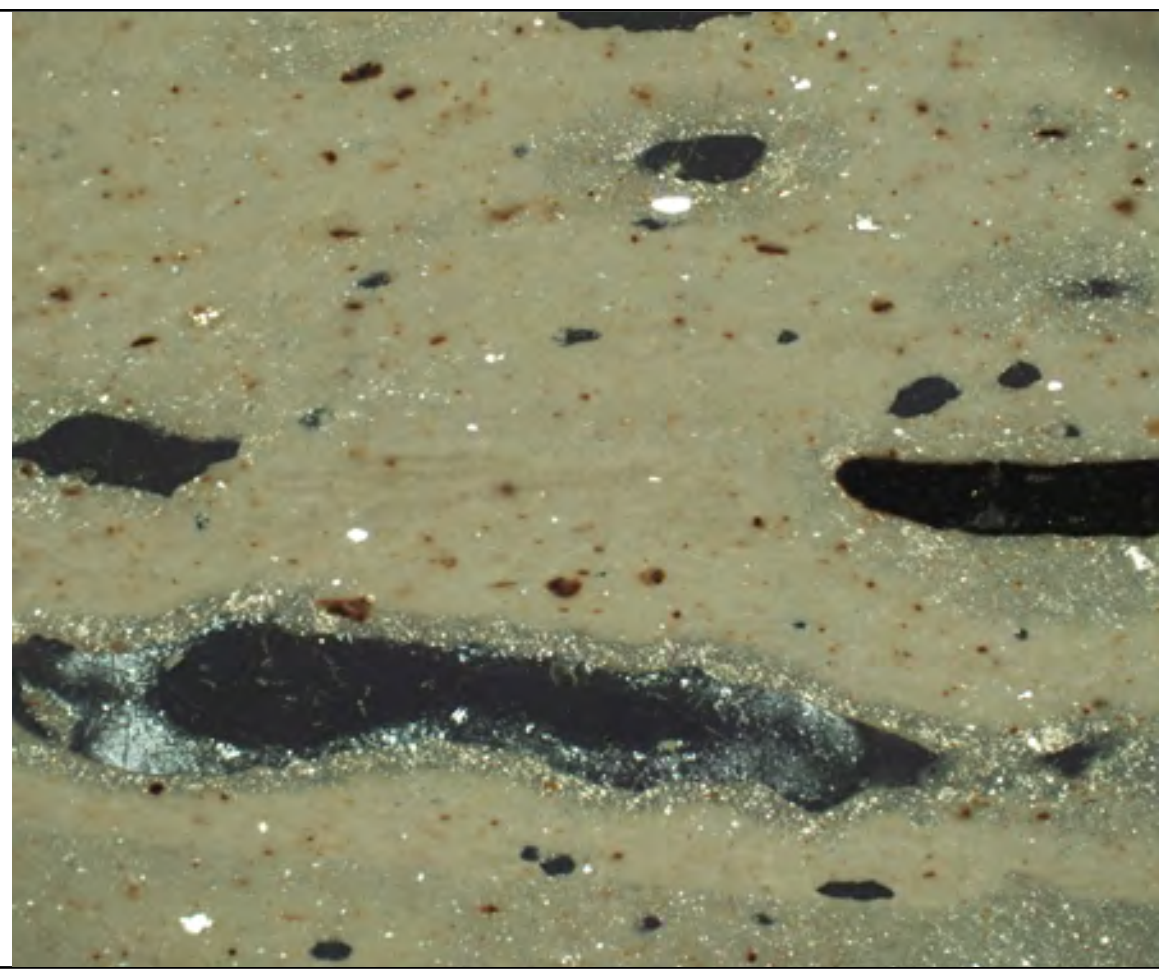

Sample **Q3**: XP, x10, field of view 1.69mm

Inclusions

Inclusions reach maximum frequencies and sizes of respectively 3-5% and 1.4mm, show equant and subrounded shapes, are open-spaced, randomly oriented, and feature a unimodal grain-size distribution, with mode: 0.25mm.

Frequent	› Quartz, equant, subrounded to subangular, 0.02-0.5mm
	› Fe-Oxide, equant, rounded, 0.02-0.4mm
Rare to very rare	› Sandstone, elongate, subrounded to subangular, 0.2-0.7mm
Rare to absent	› Shales, elongate, subangular, 0.7-1.4mm
	› Mica muscovite, elongate, angular, 0.02-0.2mm

Matrix

The matrix accounts for 88%, is micaceous and features lenses of varying colors, grain-size distributions and optical activities. In XP and PPL colors range from golden and yellowish to light brown-greenish. The optical activity ranges from inactive to highly active (grano-striated b-fabric).

Voids

The porosity ranges between 3 and 7% and is composed of parallelly oriented meso- to macrochannels, vughs and planar voids.

Petrogroups Description - QURAYYAH

Petro-loner Q37

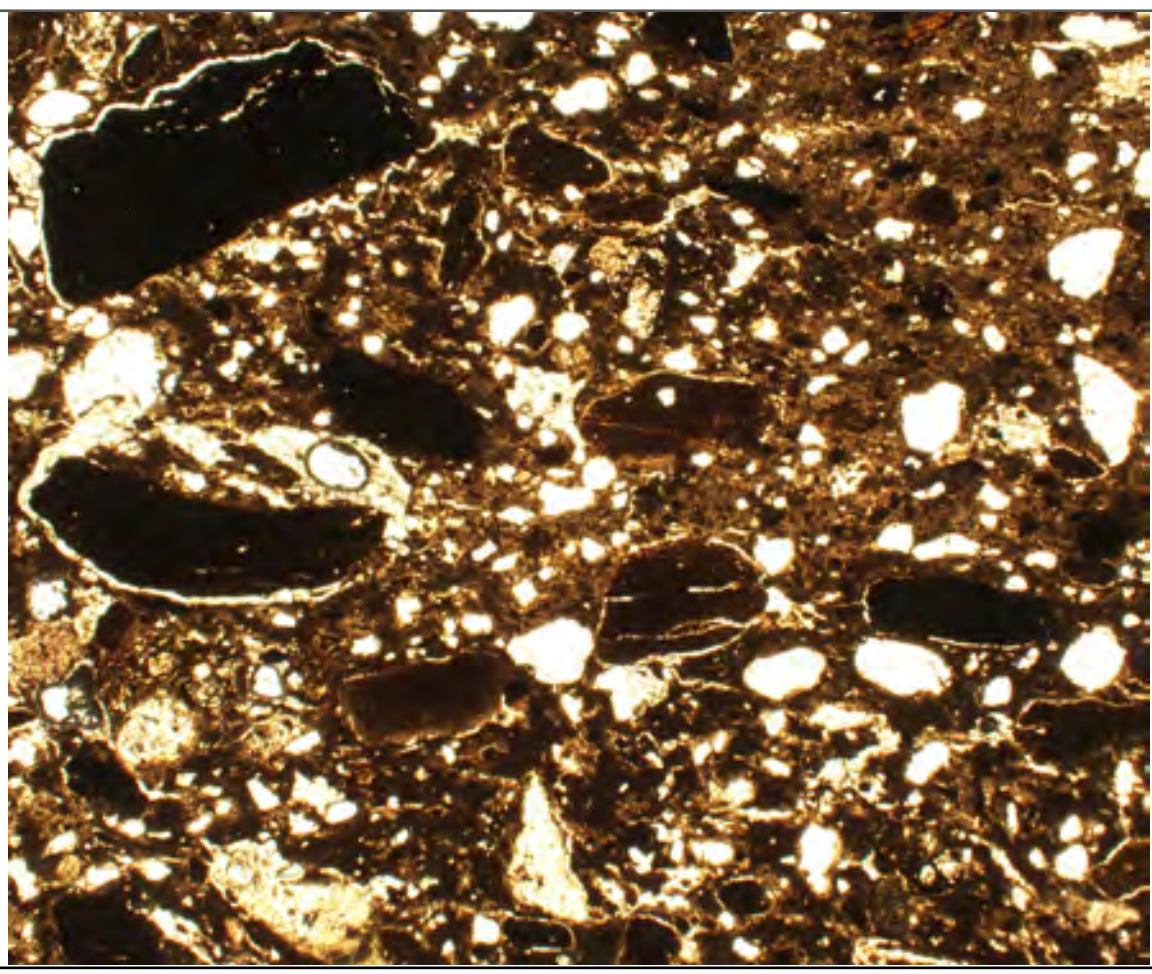

Sample **Q37**: PPL, x2.5, field of view 6.54mm

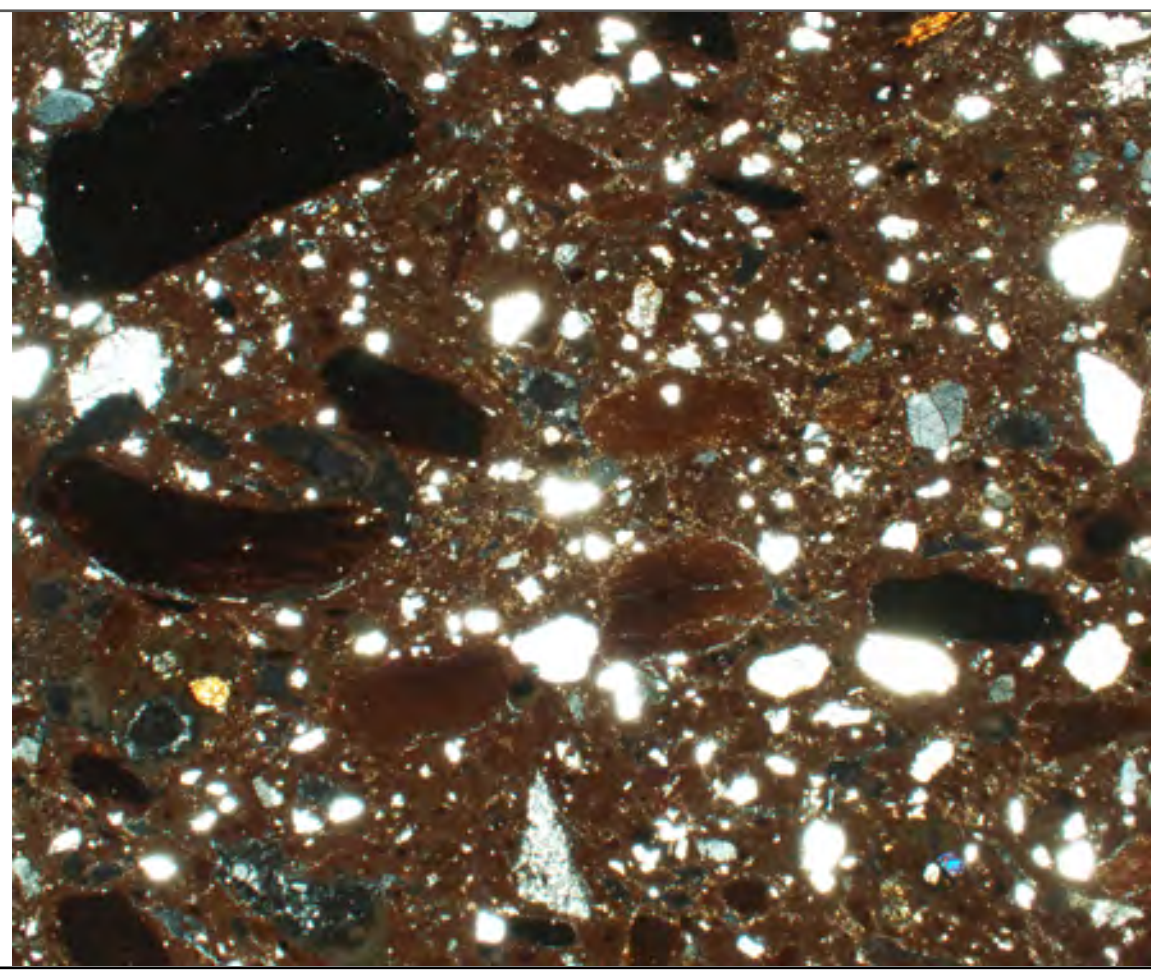

Sample **Q37**: XP, x2.5, field of view 6.54mm

Inclusions

Inclusions reach a maximum diameter and frequency of respectively 2.16mm and 15-20%, exhibit elongate to equant and subangular to subrounded shapes, are close to open-spaced, slightly to randomly oriented, and feature a bimodal grain-size distribution.

Coarse Fraction

The coarse fraction represents 50% of the total inclusions and shows sizes comprised between 0.8mm and 2.16mm.

Predominant	› Shales, elongate, angular, 1-2.16mm, mode: 1-1.5mm
Very few	› Sandstones, subangular, equant, 0.8mm
Rare	› Chert? Elongate, angular, 0.96mm

Fine Fraction

The coarse fraction represents 50% of the total inclusions and shows sizes comprised between 0.02mm and 0.8mm.

Predominant	› Quartz
Few	› Mica muscovite
Very few	› K-feldspars
	› Sandstones
	› Shales
	› Biotites
	› Olivines/Pyroxenes
Very few to rare	› Plagioclases
Rare to very rare	› Brown hornblende
	› Epidote
Very rare	› Basalt?

Matrix

The matrix accounts for 75%, is silty, homogeneous and slightly active to inactive with colors ranging from brown to reddish-brown (in PPL and XP).

Voids

The porosity accounts for 5% and is composed of randomly oriented meso- to megavughs and planar voids.

Petrogroups Description - QURAYYAH

Petro-loner Q39

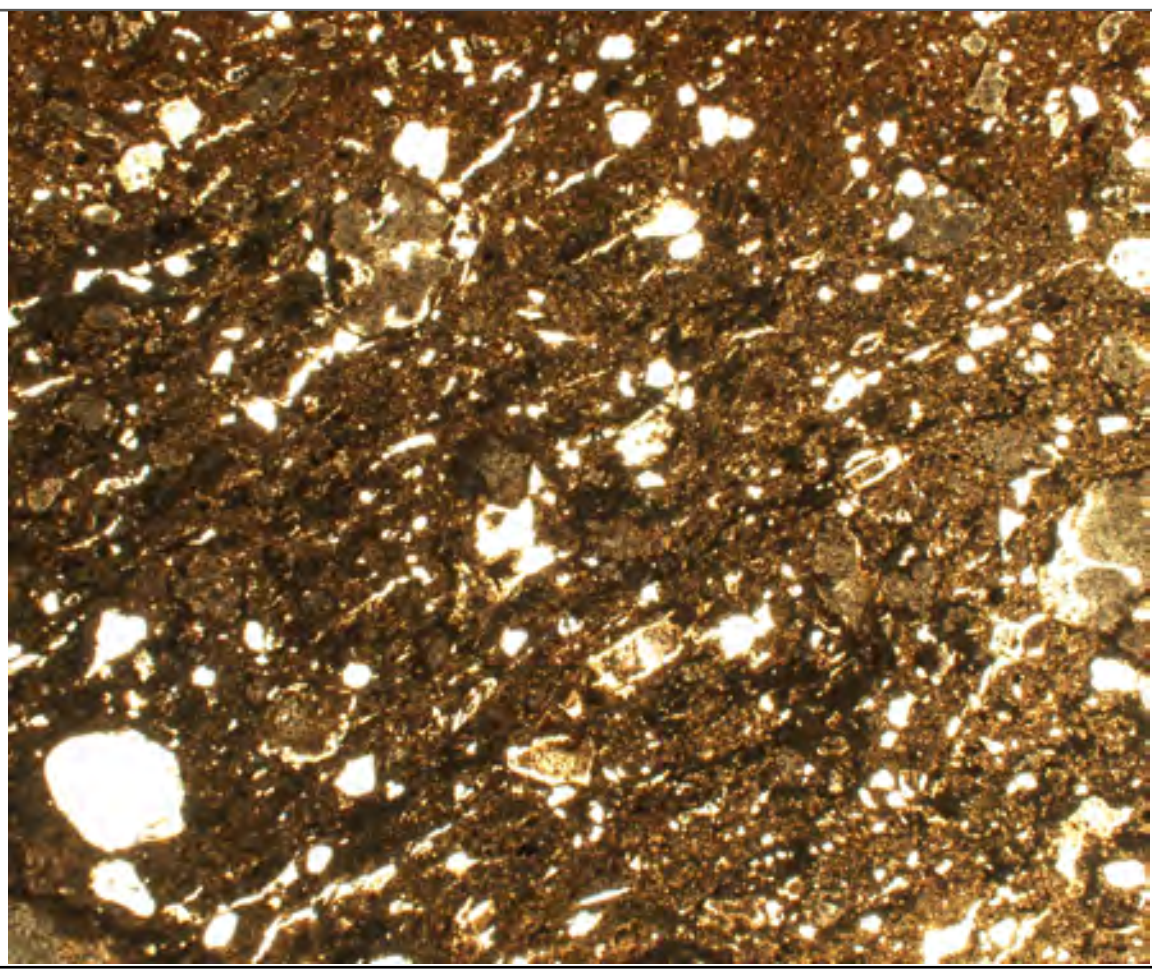

Sample **Q39**: PPL, x2.5, field of view 6.54mm

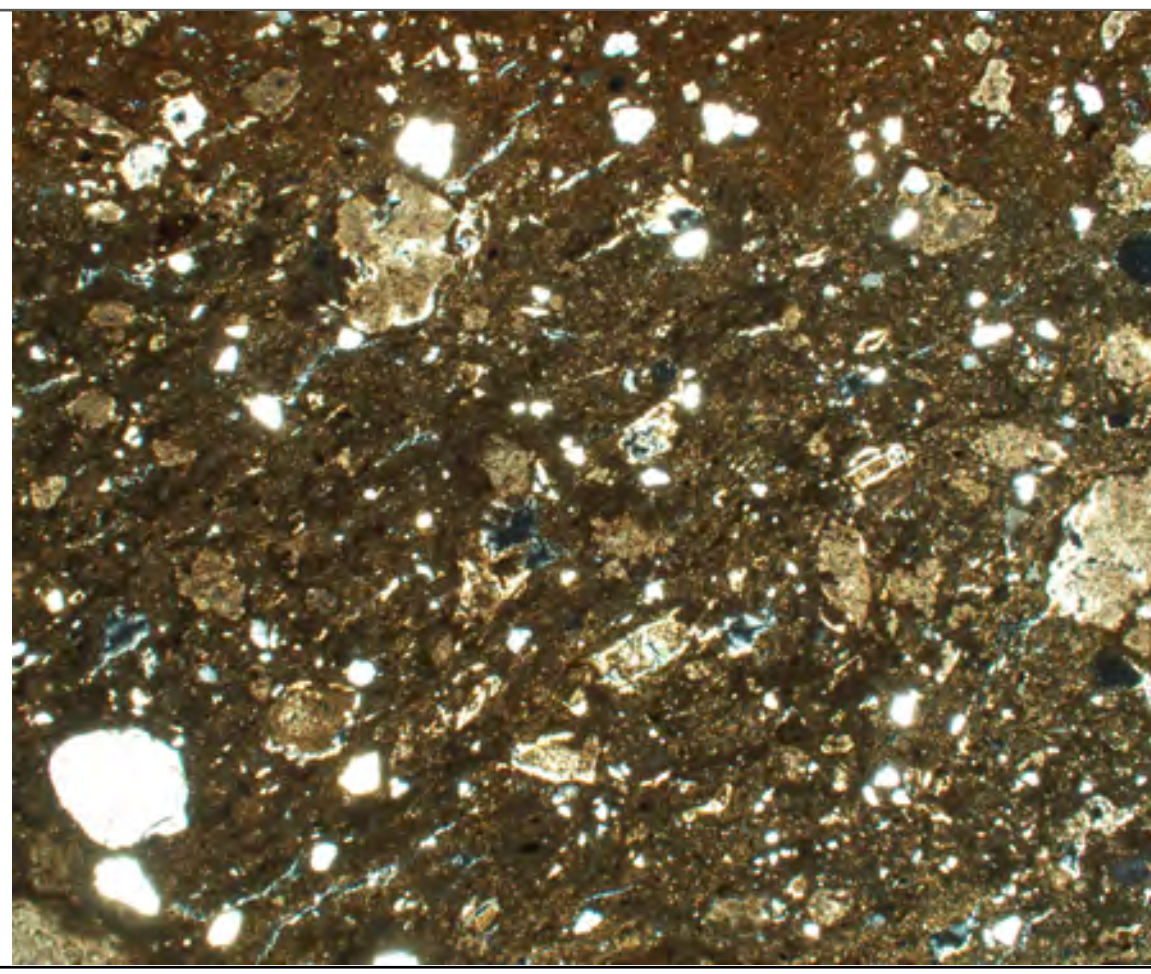

Sample **Q39**: XP, x2.5, field of view 6.54mm

Inclusions

Inclusions reach a maximum diameter and frequency of respectively 1.5mm and 10%, exhibit equant and subangular to subrounded shapes, are single to open-spaced, randomly oriented, and feature a bimodal grain-size distribution.

Coarse Fraction

The coarse fraction represents 60% of the total inclusions and shows sizes comprised between 1mm and 1.5mm.

Predominant	› Calcareous sedimentary rocks (also organogenic?), equant to elongate, subrounded to subangular, 1-1.5mm, mode: 1.3-1.4mm
Rare	› Quartz, equant, subrounded to subangular, 1mm

Fine Fraction

The coarse fraction represents 40% of the total inclusions and shows sizes comprised between 0.1mm and 0.8mm.

Predominant	› Quartz
Dominant	› Calcareous sedimentary rocks
Few	› Fe-Oxides
Very few	› Mica muscovite
Very rare	› Sandstones
	› Altered macroforaminifera?

Matrix

The matrix accounts for 85% and is slightly silty. The color is heterogeneous, band-distributed. Both in XP and PPL, reddish-brown, brown-greenish, brown and dark brown bands alternate. The matrix is slightly active.

Voids

The porosity accounts for 5% and is composed of parallelly inclined meso- to macro-planar voids.
