# Supplementary material for: Creating a brand. Genesis, transformation and diffusion of Qurayyah Painted Ware
Source: PLoS One. 2026 Aug 19;21(8):e0351296. doi: 10.1371/journal.pone.0351296 (PMC13489407; doi:10.1371/journal.pone.0351296)
Supplement: Supplementary file 9 [file pone.0351296.s009.pdf]

CREATING A BRAND.
GENESIS, TRANSFORMATION AND DIFFUSION OF QURAYYAH PAINTED WARE

Luciani M. – Fragnoli P. – Gilstrap W.D. – Sterba J.H. – Klainscek D.– Greene J.A. – Bourke S. – Donnelly P.

Corresponding author: Marta Luciani, University of Vienna, marta.luciani@univie.ac.at

……….

SUPPORTING INFORMATION 9

Detailed descriptions and micrographs of petrogroups Kheleifeh 1 and Kheleifeh 1v

All photos: rights holder William D. Gilstrap

Petrogroups Description - TELL EL-KHELEIFEH

Kheleifeh 1

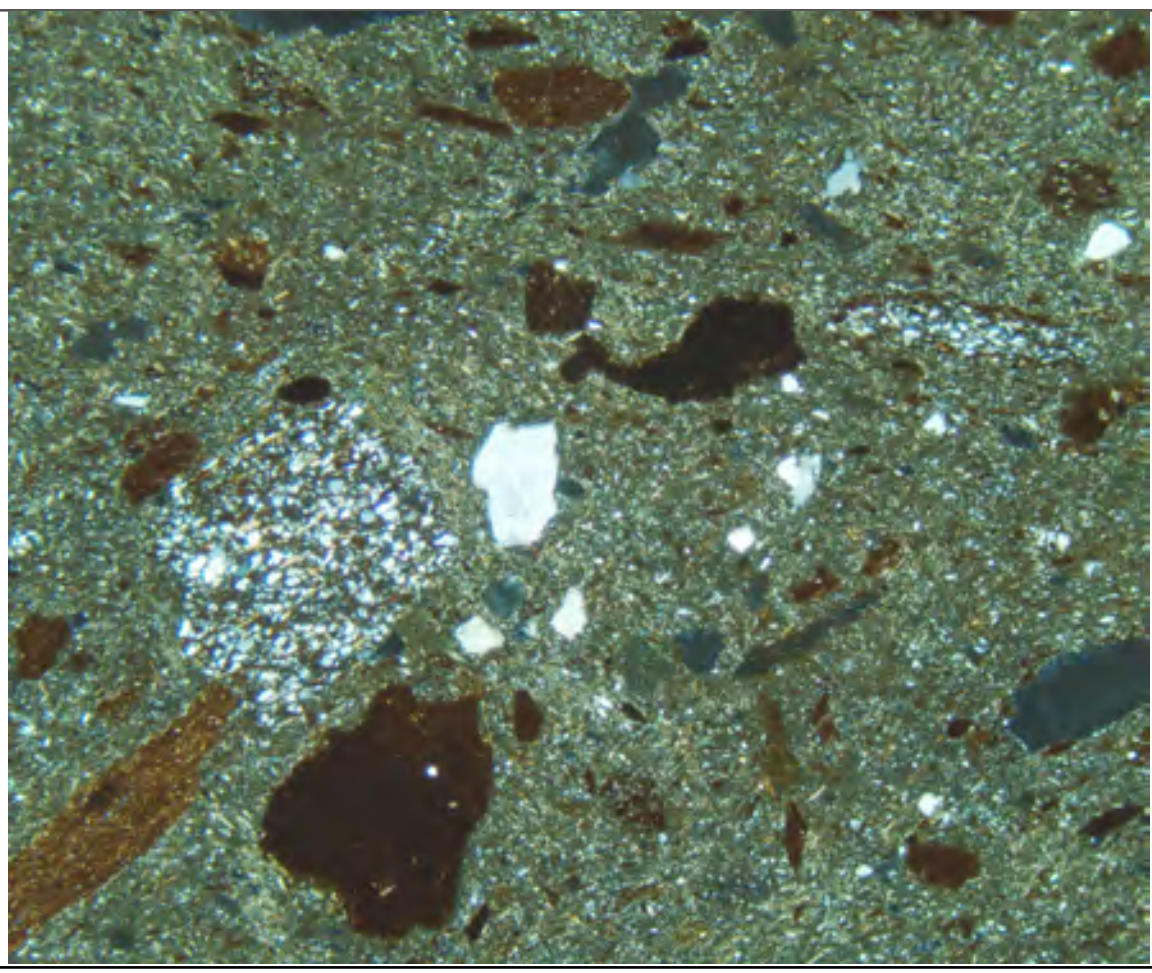

Sample **ML005**: XP, x4, field of view 4.25mm

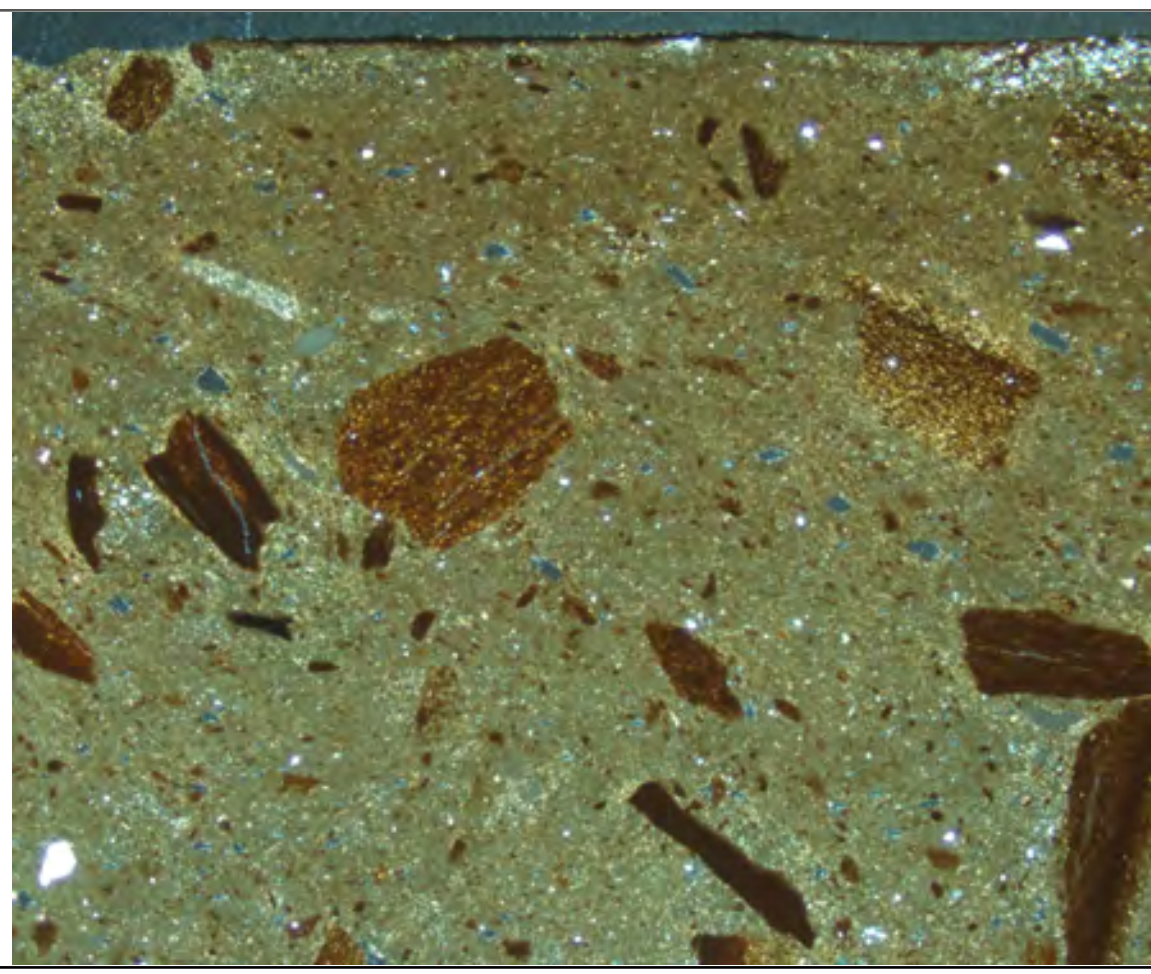

Sample **ML007**: XP, x4, field of view 4.25mm

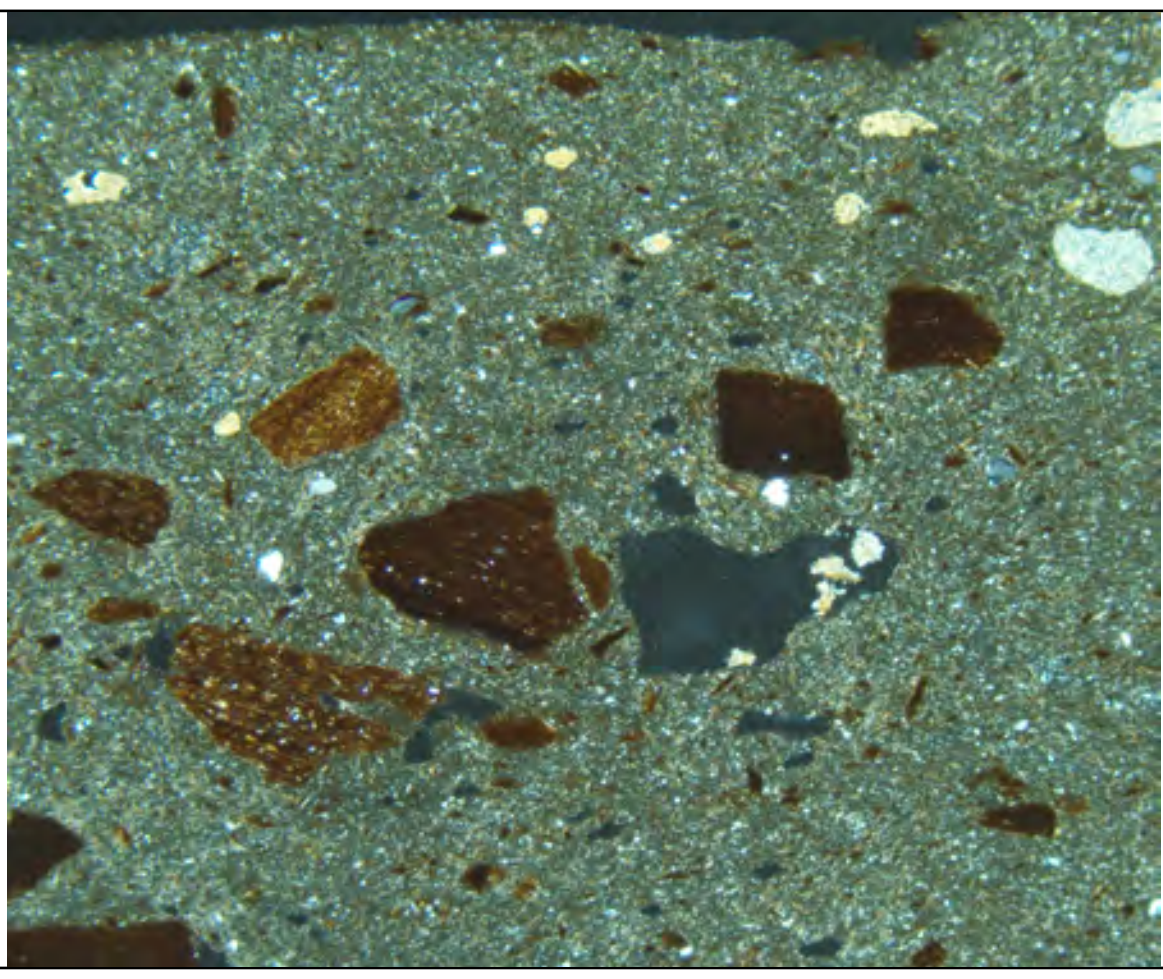

Sample **ML009**: XP, x4, field of view 4.25mm

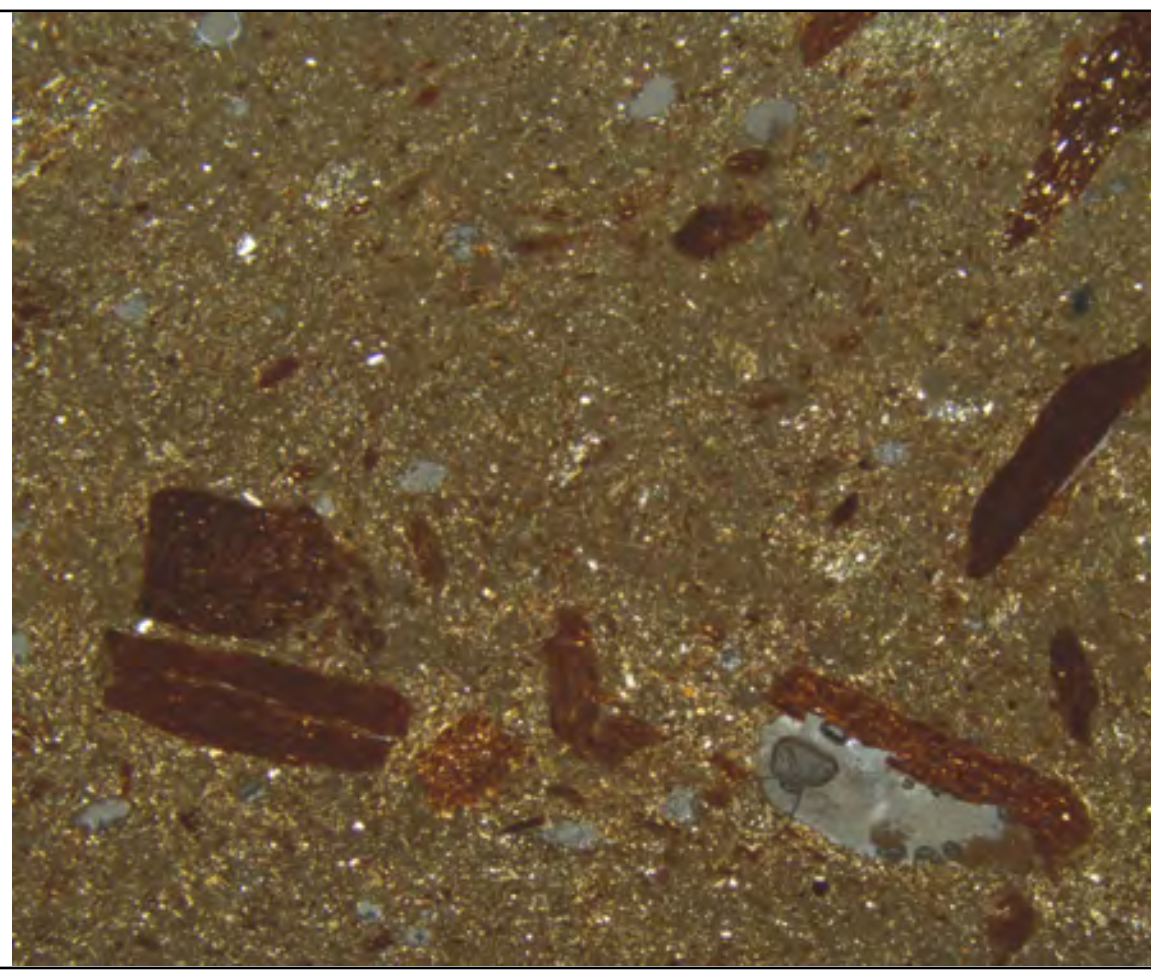

Sample **ML011**: XP, x4, field of view 4.25mm

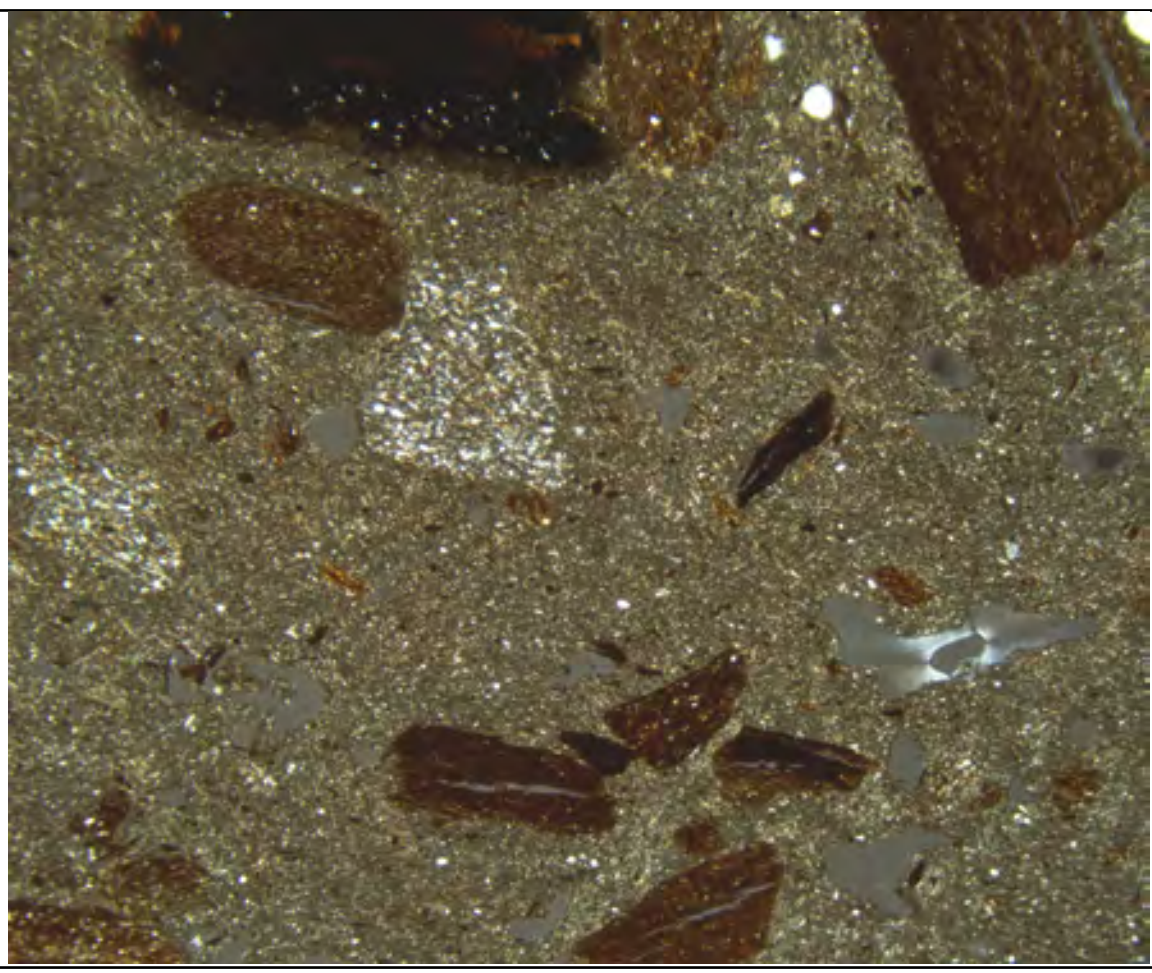

Sample **ML012**: XP, x4, field of view 4.25mm

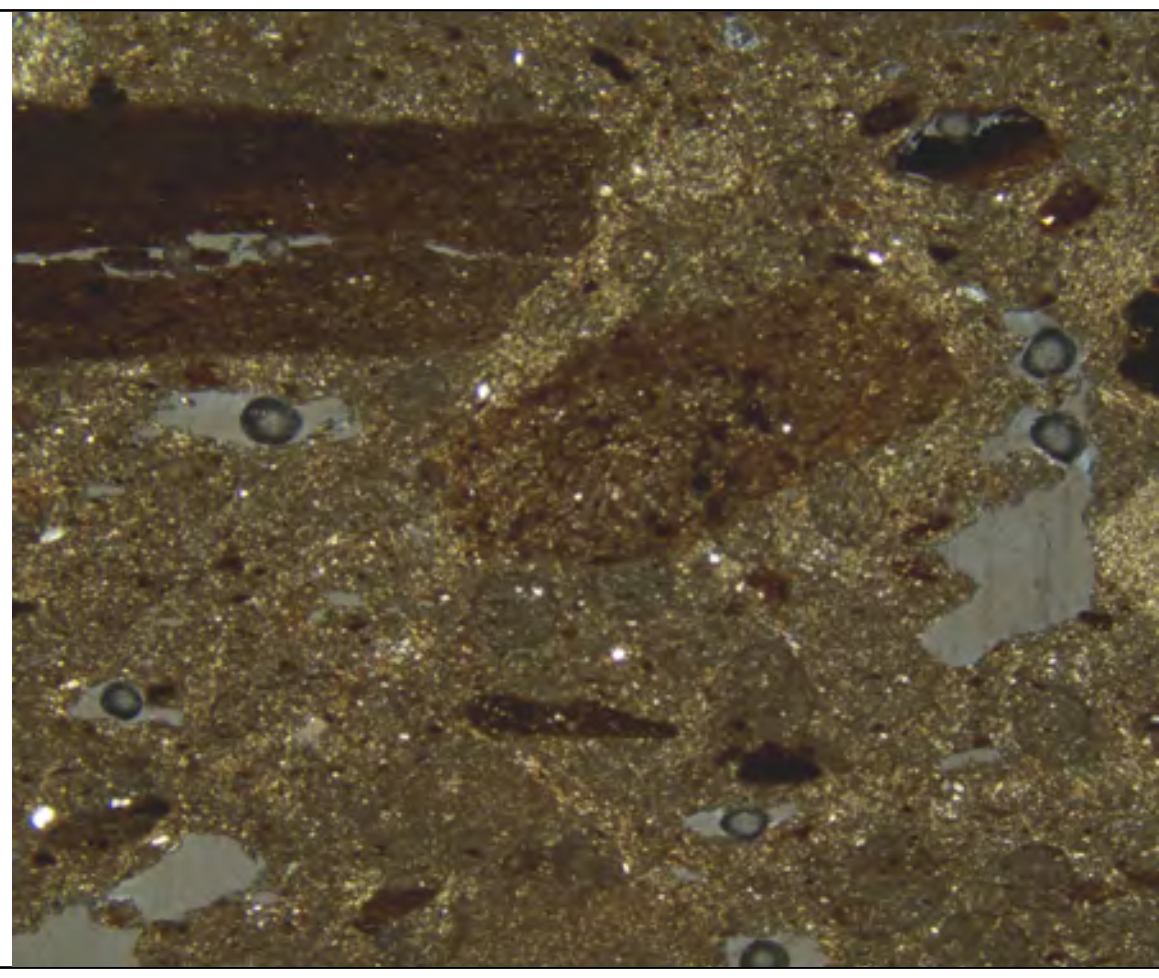

Sample **ML013**: XP, x4, field of view 4.25mm

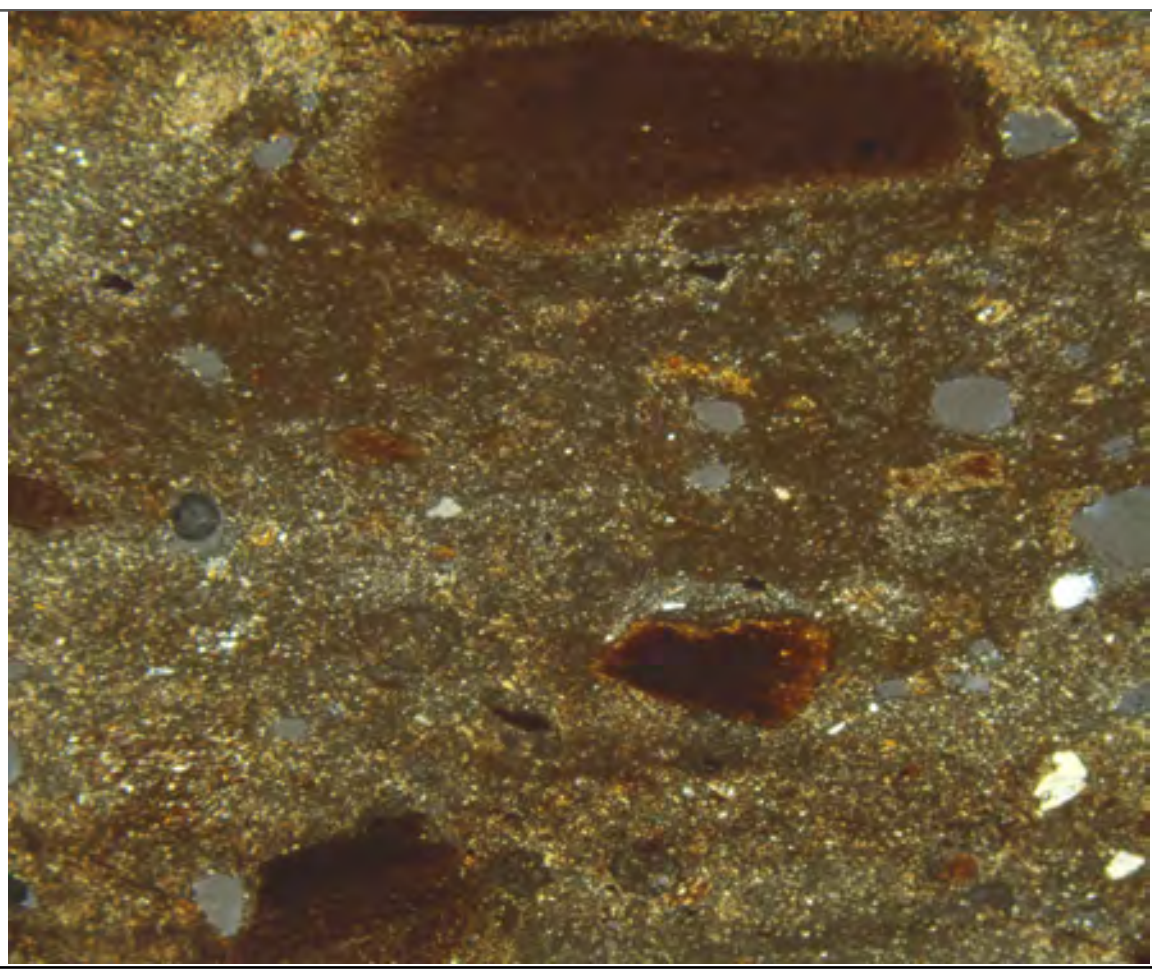
Sample **ML014**: XP, x4, field of view 4.25mm

Microstructure

Voids appear predominantly as micro- to mesovughs with common to few macrovughs. Meso-macrovughs are frequently elongated with very few demonstrating equant shape. Microvughs are predominantly equant. Voids are single to open-spaced, where inclusions are closed to open-spaced. Elongated voids and inclusions have a preferential alignment either at an oblique angle or near parallel to the vessel margins.
c:f:v = 0.025 mm 20:72:8

Groundmass

The groundmass is moderately well sorted, yet heterogeneous in texture, color and composition. The matrix is optically active, demonstrating both grano- and porostriated b-fabric texures. The domains near to the vessel margins have more concentrated optical activity suggesting an alignment of particles. Colors under cross polars (x40) vary between samples ranging from golden, yellowy brown in the domains nearest the vessel margins to pale yellow brown near the core. Under plane polars (x40) colors range from pale yellowish brown to greyish pale brown. The matrix is rich in mica (biotite? Sericite?), silicate minerals (quartz/feldspar) and brownish red oxides (hematite? Particles of Sedimentary Rock-Type 1). Micrite is rarely visible, and only as infilling of voids and very rare aggregates (ML009). The mineral composition of the groundmass is highly compatible with the sedimentary rock in both coarse and fine fractions of constituent inclusions.

Inclusions

Inclusions are frequently elongated with few equant grains. They are moderately well sorted, closed to open-spaced and show a bimodal grainsize distribution.
Coarse fraction grain size distribution: 2.9 – 0.125 mm long diameter
Fine fraction grain size distribution: 0.125 – 0.025 mm long diameter
c:f – 15:75

Coarse Fraction

Frequent	› Sedimentary rock - Type 1. 2.90 – 0.125 mm long diameter; mode: 0.25 mm long diameter; elongate, sa-a. Dark reddish brown to light brownish red under cross polars (x40), bedded shale with few to absent silicate inclusions. Rare fragments show layering with medium silt-sized grains of brown mica, quartz, plagioclase feldspar, and alkali feldspar or just quartz with fine mud between grains. Can grade into reddish opaque. Opaque matter in coarse fraction all appears to be related to this rock fragment type
Few to absent	› Sedimentary rock - Type 2. 1.225 – 0.025 mm long diameter; mode: 0.1 mm long diameter; elongate, sr. Greyish yellow siltstone comprised of micaceous layers interposed with silicate (quartz/feldspar) layers with amorphous opaque inclusions randomly dispersed throughout rock structure. Boundaries can merge into matrix
Few to very few	› Orthoclase feldspar. 0.175 – 0.025 mm long diameter; equant, sr-sa. Simple twins
Very few to rare	› Quartz. 0.15 – 0.025 mm long diameter; mode: mm long diameter; equant, sa-a. Monocrystalline. Straight extinction
Rare to absent	› Microcline feldspar. Xx – 0.025 mm long diameter; equant, sr-sa
	Micrite aggregate. 0.125 – 0.075 mm long diameter; equant, wr

Fine Fraction

Common	› Mica – Biotite/sericite
Few	› Quartz/feldspar
	› Opaques – related to rock fragments-Type 1
	› Rock fragments

Petrogroups Description - TELL EL-KHELEIFEH

Kheleifeh 1v (variant)

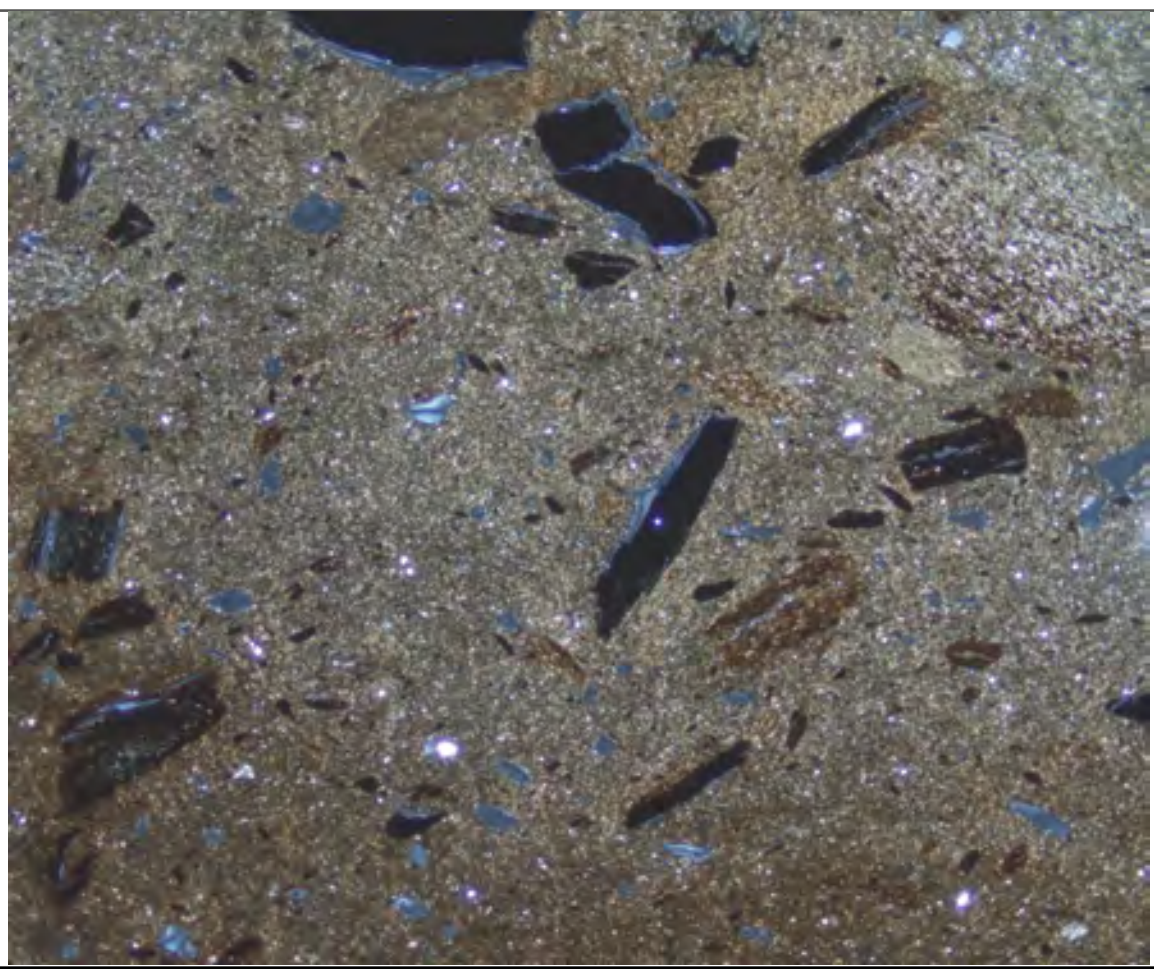	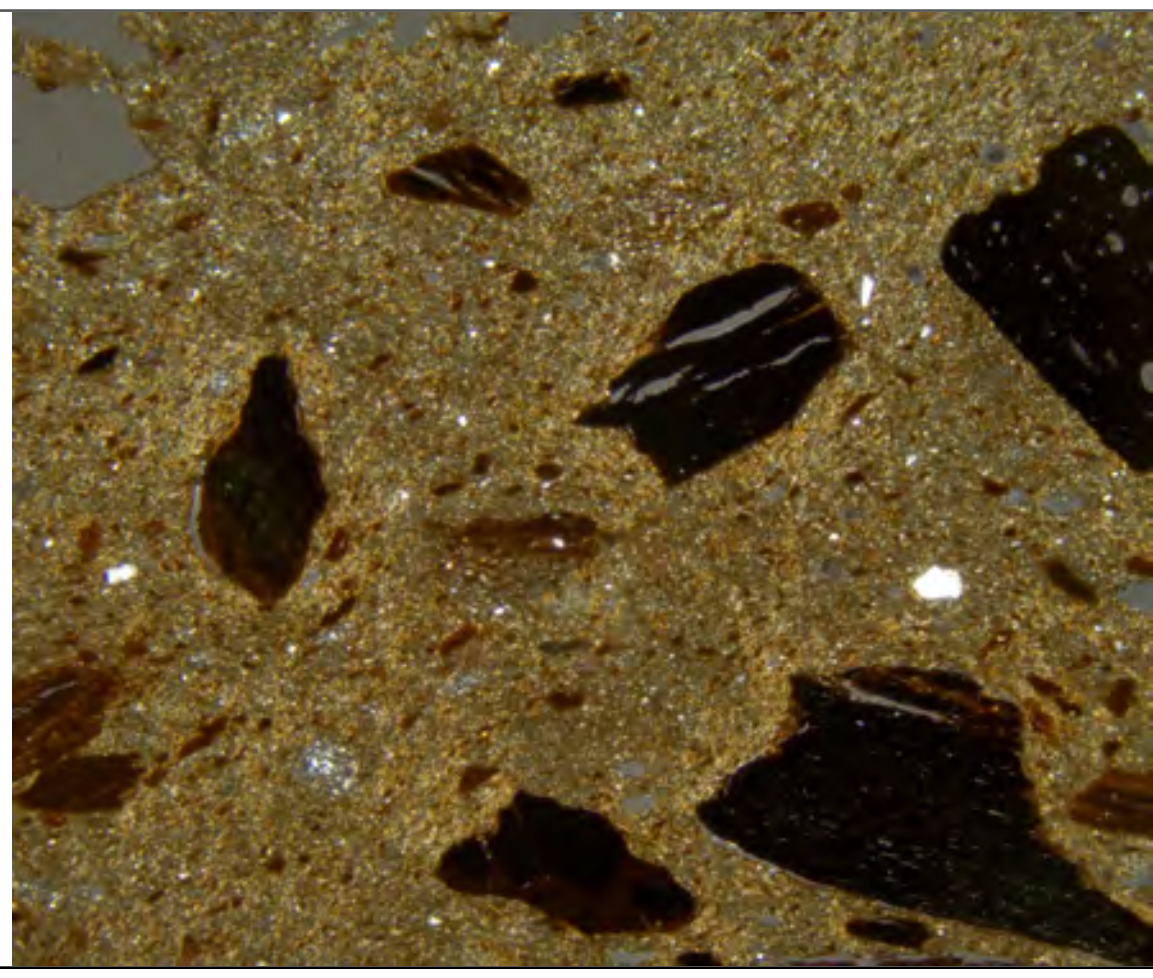
ML008: XP, x4, field of view 4.25mm	ML010: XP, x4, field of view 4.25mm

Microstructure
Same as Tell el-Kheleifeh 1

Groundmass
Same as Tell el-Kheleifeh 1 with the following exception:

- ML010 has a reduced core with oxidation evident only within approximately 0.75-1.75 mm of the domain near the vessel margins.

Inclusions
Same as Tell el-Kheleifeh 1 with the following exception:

- Rock fragment similar to Sedimentary Type 1, however it is brownish grey to black in XP (40x) and grey to black in PP (40x). Few examples show moderately fine micacious mineral inclusions. It may be a different rock type, but it is difficult to discern with so few examples.

Notes on Kheleifeh 1
The groundmass is poorly mixed with textural features that indicate the paste recipe may contain more than one clay component. High optical activity and darkening of the body core suggest that the ceramic was fired in fairly low temperatures (<650°C [Kilikoglou, V, Vekinis, G, Maniatis Y. Toughening of ceramic earthenwares by quartz inclusions: An ancient art revisited. Acta Metallurgica et Materialia. 1995; 43(8): 2959-2965.]) for a short period of time that did not allow the core to fully oxidize. Sedimentary rock fragment Type 2 has a composition concordant with the matrix and may represent the parent rock from which the clay matrix was geologically derived. In the coarse fraction, those rare fragments in which clasts can be identified contain layers of brown/biotite mica, quartz, plagioclase and alkali felspar intercalated with fine mud. The fragments indicate that the bedded rock perhaps grades into mudstone.

Notes on Kheleifeh 1v
Although similar to the inclusions of both Type 1 and Type 2 described in Kheleifeh 1, rock fragments in Kheleifeh 1v range from dark reddish brown to greyish black and contain fine-moderately fine particles of minerals, likely mica. The dark color of these inclusions may be due to the incomplete oxidation during firing of ML010, but there is no such evidence to explain reduced iron rich sedimentary rock fragments in sample ML008. Differences in raw material selection or materials processing may be a function of time as sample ML010 and possibly also ML008 appear to be from a later period (Middle Iron Age) than the rest.
