# Supplementary material for: Creating a brand. Genesis, transformation and diffusion of Qurayyah Painted Ware
Source: PLoS One. 2026 Aug 19;21(8):e0351296. doi: 10.1371/journal.pone.0351296 (PMC13489407; doi:10.1371/journal.pone.0351296)
Supplement: Supplementary file 10 [file pone.0351296.s011.pdf]

CREATING A BRAND.
GENESIS, TRANSFORMATION AND DIFFUSION OF QURAYYAH PAINTED WARE

Luciani M. – Fragnoli P. – Gilstrap W.D. – Sterba J.H. – Klainscek D.– Greene J.A. – Bourke S. – Donnelly P.

Corresponding author: Marta Luciani, University of Vienna, marta.luciani@univie.ac.at

SUPPORTING INFORMATION 11

Element by element comparison of chemical groups V032 and V033

The V033 sherds were allocated into a separate group from V032 due to discrepancies in several elements, most prominently in K, Zn, Cs, Eu, Rb, Sm, and Tb (see S11 Graph). This difference is also reflected in the macroscopic fabrics: samples belonging to group V033 contain no red shale inclusions, whereas all samples from group V032 do. As we increase the number of analyzed samples, the amount of groups and clusters is likely to increase.

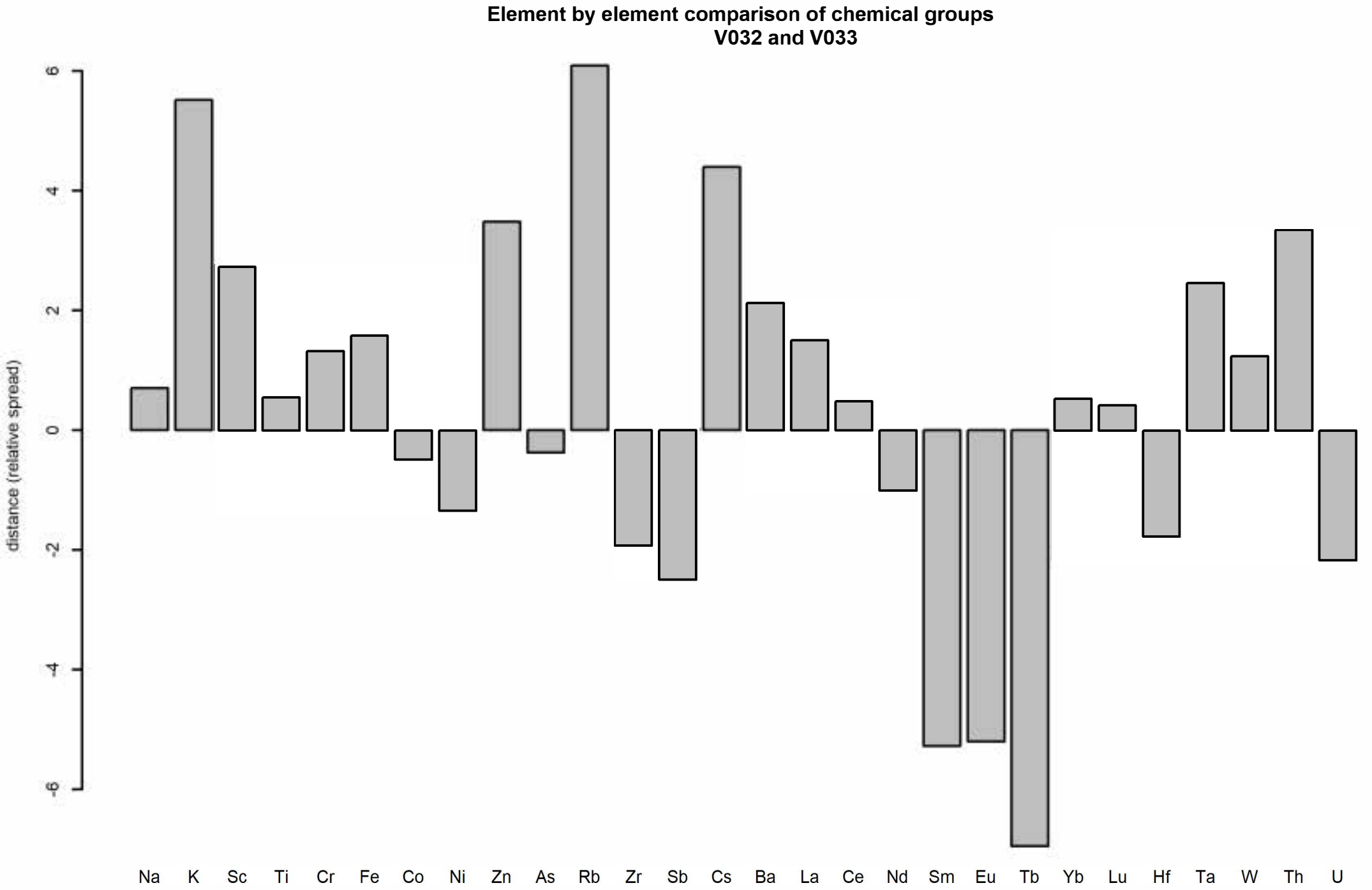
Element by element comparison of chemical groups
V032 and V033
distance (relative spread)
6
4
2
0
-2
-4
-6
Na
K
Sc
Ti
Cr
Fe
Co
Ni
Zn
As
Rb
Zr
Sb
Cs
Ba
La
Ce
Nd
Sm
Eu
Tb
Yb
Lu
Hf
Ta
W
Th
U
