# Supplementary material for: Creating a brand. Genesis, transformation and diffusion of Qurayyah Painted Ware
Source: PLoS One. 2026 Aug 19;21(8):e0351296. doi: 10.1371/journal.pone.0351296 (PMC13489407; doi:10.1371/journal.pone.0351296)
Supplement: Supplementary file 12 [file pone.0351296.s010.zip › S10_Table/S10_Table_Caption.pdf]

CREATING A BRAND.
GENESIS, TRANSFORMATION AND DIFFUSION OF QURAYYAH PAINTED WARE

Luciani M. – Fragnoli P. – Gilstrap W.D. – Sterba J.H. – Klainscek D.– Greene J.A. – Bourke S. – Donnelly P.

Corresponding author: Marta Luciani, University of Vienna, marta.luciani@univie.ac.at

....

SUPPORTING INFORMATION 10

Raw data from NAA analysis on samples from Qurayyah and Tell el-Kheleifeh.

All data are given in µg/g, including measurement errors.
"BDL" references values that were below the limit of detection.
