# Supplementary material for: Creating a brand. Genesis, transformation and diffusion of Qurayyah Painted Ware
Source: PLoS One. 2026 Aug 19;21(8):e0351296. doi: 10.1371/journal.pone.0351296 (PMC13489407; doi:10.1371/journal.pone.0351296)
Supplement: Supplementary file 13 [file pone.0351296.s012.zip › S12_Table_Caption.pdf]

CREATING A BRAND.
GENESIS, TRANSFORMATION AND DIFFUSION OF QURAYYAH PAINTED WARE

Luciani M. – Fragnoli P. – Gilstrap W.D. – Sterba J.H. – Klainscek D.– Greene J.A. – Bourke S. – Donnelly P.

Corresponding author: Marta Luciani, University of Vienna, marta.luciani@univie.ac.at

....

SUPPORTING INFORMATION 12

Raw data from NAA analysis on pottery from Pella.

NAA processed in the Becquerel Laboratory (2003) at Australia's Nuclear Science and Technology Organisation (ANSTO) in Sydney
